# Marine Oil Supplements for Arthritis Pain: A Systematic Review and Meta-Analysis of Randomized Trials

**DOI:** 10.3390/nu9010042

**Published:** 2017-01-06

**Authors:** Ninna K. Senftleber, Sabrina M. Nielsen, Jens R. Andersen, Henning Bliddal, Simon Tarp, Lotte Lauritzen, Daniel E. Furst, Maria E. Suarez-Almazor, Anne Lyddiatt, Robin Christensen

**Affiliations:** 1 Musculoskeletal Statistics Unit, The Parker Institute, Bispebjerg and Frederiksberg Hospital, 2000 Copenhagen F, Denmark; ninnaks@hotmail.com (N.K.S.); sabrina.mai.nielsen@regionh.dk (S.M.N.); henning.bliddal@regionh.dk (H.B.); simon.tarp@regionh.dk (S.T.); 2 Department of Nutrition, Exercise and Sports, University of Copenhagen, 1958 FC Copenhagen, Denmark; jra@nexs.ku.dk (J.R.A.); ll@nexs.ku.dk (L.L.); 3 Division of Rheumatology, University of California, Los Angeles, CA 90095, USA; defurst@mednet.ucla.edu; 4 The University of Texas MD Anderson Cancer Center, Houston, TX 77030, USA; msalmazor@mdanderson.org; 5 Musculoskeletal Group, Cochrane Collaboration, Ottawa, ON K1H 8L6, Canada; lyddiatt@lyddiatt.ca

**Keywords:** arthritis, marine oil, fish oil, joint pain, rheumatology, complementary medicine, meta-analysis, randomized controlled trials

## Abstract

Arthritis patients often take fish oil supplements to alleviate symptoms, but limited evidence exists regarding their efficacy. The objective was to evaluate whether marine oil supplements reduce pain and/or improve other clinical outcomes in patients with arthritis. Six databases were searched systematically (24 February 2015). We included randomized trials of oral supplements of all marine oils compared with a control in arthritis patients. The internal validity was assessed using the Cochrane Risk of Bias tool and heterogeneity was explored using restricted maximum of likelihood (REML)-based meta-regression analysis. Grading of Recommendations Assessment, Development and Evaluation (GRADE) was used to rate the overall quality of the evidence. Forty-two trials were included; 30 trials reported complete data on pain. The standardized mean difference (SMD) suggested a favorable effect (−0.24; 95% confidence interval, CI, −0.42 to −0.07; heterogeneity, *I*^2^ = 63%. A significant effect was found in patients with rheumatoid arthritis (22 trials; −0.21; 95% CI, −0.42 to −0.004) and other or mixed diagnoses (3 trials; −0.63; 95% CI, −1.20 to −0.06), but not in osteoarthritis patients (5 trials; −0.17; 95% CI, −0.57–0.24). The evidence for using marine oil to alleviate pain in arthritis patients was overall of low quality, but of moderate quality in rheumatoid arthritis patients.

## 1. Introduction

Arthritis is a musculoskeletal disorder, resulting in joint pain, swelling, stiffness and restricted movement [1,2]. In the period 2010–2012, 22.7% of American adults reported having medically-diagnosed arthritis [3]. The two most common types of arthritis are rheumatoid arthritis (RA) and osteoarthritis (OA). Osteoarthritis is a heterogeneous, degenerative disease exhibiting inflammatory components, associated with multiple risk factors [4]. In contrast, RA is an autoimmune disease involving higher levels of synovitis (joint inflammation) than OA [2,4]. The extent of synovitis has been shown to be associated with pain severity [5,6]. Arthritis patients consider pain the most hampering symptom [7], and the use of nonsteroidal anti-inflammatory drugs (NSAIDs) is substantial across arthritis diagnoses [8,9]. However, NSAIDs are known to cause serious gastrointestinal and occasionally cardiovascular adverse effects [10,11], prompting the search for alternative treatments.

Marine oil is thought to have an analgesic effect in arthritis as a likely consequence of its high content of docosahexaenoic acid (DHA; 22:6 *n*-3) and eicosapentaenoic acid (EPA; 20:5 *n*-3). Arachidonic acid (AA; 20:4 *n*-6), as well as DHA and EPA, are used in the production of lipid mediators (e.g., eicosanoids), which are involved (among other functions) in the regulation of inflammation. However, the mediators produced from DHA and EPA shift the balance toward resolution [12]. Thus, supplementation with EPA- and DHA-rich oil could exert an anti-inflammatory effect [13], making it a possible treatment for arthritis pain.

Four meta-analyses have compared fish oil or *n*-3 polyunsaturated fatty acid (PUFA) supplements with control treatment in patients with musculoskeletal complaints [14,15,16,17]. Three meta-analyses concluded that such supplements were effective [14,15,16], whereas the fourth review did not find any effect [17], likely because of different inclusion criteria. In contrast to the previous meta-analyses, our review included a comprehensive search strategy and assessment of the risk of bias and the quality of evidence. Vegetable oils were not included, as these provide α-linolenic acid (ALA; 18:3 *n*-3), from which only marginal amounts of EPA and DHA can be produced in the human body [18,19].

A previous US survey suggested that approximately 90% of arthritis patients have used, or were using, complementary therapies [20]. Therefore, consumers and health care practitioners need to know whether oral marine oil can reduce arthritis pain.

The aim of this systematic review was to evaluate the effect of marine oil across a broad range of arthritic diseases. Our primary outcome was pain. Secondary outcomes were physical function, inflammation, number of completers (tolerance), withdrawals due to adverse events, and number of serious adverse events (SAEs). Our use of the term “marine oil” refers to all oils of marine origin (e.g., oil from whole fish, seals, and mussels).

## 2. Materials

### 2.1. Protocol

Study selection, assessment of eligibility criteria, data extraction, and statistical analyses were performed based on a predefined protocol registered on PROSPERO (CRD42015016817) following the guidelines from EQUATOR [21] on systematic reviews (PRISMA statement [22]).

### 2.2. Data Sources and Searches

The search was conducted on 24 February 2015 in Medical Literature Analysis and Retrieval System Online (MEDLINE), the Cochrane Library, Excerpta Medica Database (EMBASE), ClinicalTrials.gov, and the World Health Organization International Clinical Trial Registry Platform portal (ICTRP) as recommended by the Cochrane Musculoskeletal Group [23], and in the Web of Science (search strategies are available in Table S1). Reference lists from relevant publications were screened. The initial screening of the records was done by two reviewers (N.K.S. and S.M.N.), and the subsequent assessment on full texts was done independently by the same two reviewers. Disagreements were resolved by consensus or by consulting another reviewer (R.C. or H.B.).

### 2.3. Study Selection

Eligible studies were all randomized controlled trials comparing all types of marine oil supplements (i.e., oils of marine origin such as oil from whole fish, seals, or mussels) with a control treatment applying an add-on design in patients diagnosed with any type of arthritis for a minimum duration of two weeks. No restrictions were applied on age or gender of the participants, dosage, or publication date. Reporting the outcomes of interest was not a criterion for entering the systematic review. However, trials included in the main meta-analysis for each outcome had to present complete data (i.e., quantitative data or comprehensive figures), allowing data extraction. Reports with incomplete data for the outcomes of interest were included in a sensitivity analysis using null imputations. Reports had to have at least an abstract written in English, Danish, Swedish, or Norwegian.

### 2.4. Data Extraction and Quality Assessment

The preferred time point of measurements was the last day of the intervention or as late as possible when the participants were still receiving the intervention. According to the guidance document provided by the Cochrane Musculoskeletal Group (aligned with Outcome Measures in Rheumatology [OMERACT]), there is currently no consensus on a generic core outcome measurement instrument set that would apply across all musculoskeletal conditions [23]. However, pain remains a construct of major importance to all (rheumatology) stakeholders (e.g., patients and physicians) [24]. Thus for the purpose of this review we considered pain the primary outcome [25]. For the function construct, we used the most appropriate functional tests (e.g., grip strength was preferred for measuring function in RA, and a walk test was preferred for measuring function in hip or knee OA). We preferred the most commonly used measures of inflammation (e.g., C-reactive protein, CRP, was preferred over erythrocyte sedimentation rate). In case of doubt, we used the outcome chosen by a blinded rheumatologist (H.B.). Authors were contacted in order to obtain data when it was not extractable.

Trials with low internal validity may distort the results from meta-analyses [26], so trials were assessed using the Cochrane Risk of Bias tool [27]. There is evidence that statistically significant outcomes have higher odds of being fully reported [28]; therefore, outcome-reporting bias was assessed using the tool developed by Dwan et al. [29]. Two reviewers (N.K.S. and S.M.N.) independently extracted data and assessed risk of bias. In case of disagreement, consensus was reached by discussion or by consulting a third reviewer (R.C. or S.T.).

### 2.5. Data Synthesis and Analysis

Due to different ways of measuring pain, physical function, and inflammation, treatment effect sizes for each of the studies were expressed as standardized mean differences (SMDs) by dividing the difference in mean values by the pooled standard deviation (SD) for the given outcome. A correction was applied by default by calculating Hedges’s g [30] and the variance (SE^2^) was calculated based on the SMD and number of patients in each group [31]. For trials with more than one intervention group, the number of patients in the control group was divided by the number of comparisons, hence avoiding double counting of patients and increasing the standard errors, resulting in more correct estimates. Mean differences at follow-up were used when differences in change from baseline were not available. A negative SMD for pain and inflammation indicates a beneficial (reducing) effect of the intervention, where a positive SMD for physical function indicates a beneficial (increasing) effect. Risk ratios (RRs) were calculated for tolerance, withdrawals due to adverse events, and number of SAEs [23]. The overall SMDs were transformed into a measure that is easier to interpret [23]; the SMD for pain was transformed into average improvement in percentage compared to control [32], assuming an average for pain of 60 mm on a 100-mm visual analog scale (VAS) with an SD of 20 mm based on a cohort of arthritis patients [33]. For inflammation, a baseline value of 3.8 mg/dL CRP and an SD of 5.9 mg/dL were assumed (equal to the mean baseline value and SD, respectively, of the included studies).

Heterogeneity was investigated using forest plots and the heterogeneity (*I*^2^) statistic [34,35]. Random-effects models, based on restricted maximum of likelihood (REML), were used as default option, whereas fixed-effect models were applied as sensitivity analysis.

Sensitivity analyses were conducted by including trials with high risk of outcome-reporting bias and incomplete data on the outcomes of interest by using null imputation, based on the assumption that they did not find any effect. This was done by imputing the value, 0, for the SMDs, and calculating the confidence intervals from the SE^2^ as usual, i.e., based on the SMD and number of patients in each group [36]. The risk of small-study (e.g., publication) bias across studies was assessed using funnel plots as well as a test for funnel plot asymmetry [37]. Univariate REML-based meta-regression analyses on pain were used to assess the effect of each risk of bias domain and funding source. Analyses were performed using R Software (version 3.2.0) [38].

GRADE (Grading of Recommendations Assessment, Development and Evaluation) was used to rate the overall quality of the evidence based on the assessed risk of bias, publication bias, imprecision, inconsistency, indirectness, and to some degree interpreted this with the apparent magnitude of effect as well [39]. The GRADE ratings reflect the confidence in the results.

### 2.6. Additional Analyses

In order to investigate possible sources of heterogeneity, additional analyses of pain were performed, stratifying the available trials according to trial characteristics (e.g., type of diagnosis) using univariate REML-based meta-regression. Furthermore, a limited number of post hoc analyses were carried out, stratifying for the type of marine oil for pain, and for trial characteristics and risk of bias domains for function and inflammation.

## 3. Results

### 3.1. Study Selection

**Figure 1:**
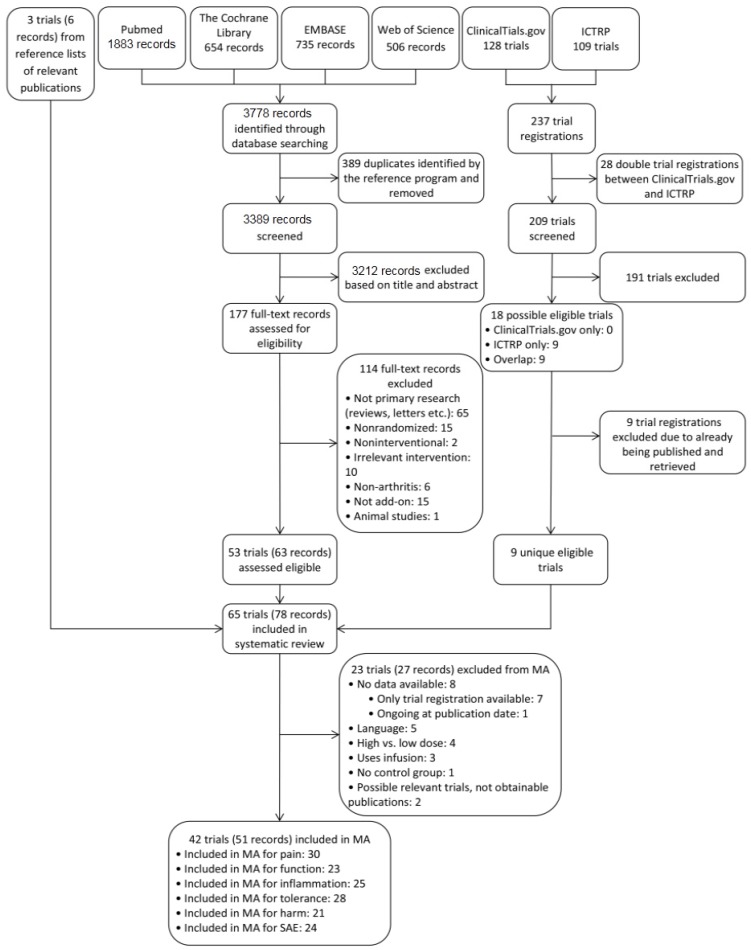
Flow diagram showing the selection of trials. MA: meta-analysis, ICTRP: World Health Organization International Clinical Trial Registry Platform portal, SAE: serious adverse event.

The search strategy identified 3389 records (Figure 1). Of these, 3212 were excluded because they did not meet the inclusion criteria, and the subsequent full-text assessment resulted in 63 records describing 53 trials eligible for the systematic review. Searching the trial registries ClinicalTrials.gov and the World Health Organization International Clinical Trial Registry Platform portal (ICTRP) contributed an additional nine unique trials for the systematic review; however, none of these trials were published, and no resulting data could be obtained from author contact so they could not be included in the meta-analysis. Review of reference lists of relevant publications identified three potentially relevant trials; one was included [40], and two could not be retrieved [41,42]. In total, 78 records describing 65 trials met the inclusion criteria for the systematic review; of these, 51 records describing 42 trials [40,43,44,45,46,47,48,49,50,51,52,53,54,55,56,57,58,59,60,61,62,63,64,65,66,67,68,69,70,71,72,73,74,75,76,77,78,79,80,81,82,83,84,85,86,87,88,89,90,91,92] were eligible for inclusion in the meta-analyses (reference lists are provided in Section S1). Reasons for exclusion are stated in Figure 1 (reference lists are provided in Sections S2 and S3). One trial was ongoing at the time of publication [93], and we received no answer after contacting the author. Authors of 20 trials were contacted for additional information; four [46,72,89,90] provided additional information and one [75] provided additional data.

### 3.2. Study Characteristics

Study characteristics of the 42 trials included in the analyses are presented in Table 1. The trials used treatment durations from 2 weeks to 18 months, with doses of EPA from 0.013 to 4.050 g/day, and doses of DHA from 0.010 to 2.700 g/day. Most trials used marine oil from whole fish, but some used cod liver oil, mussel extracts, seal oil, and krill oil. Thirty-two trials examined RA, 6 trials examined OA, and 4 trials examined other/mixed types of arthritis (juvenile arthritis, number of trials: *k* = 1; polyarticular psoriatic arthritis, *k* = 1; mixed diagnoses of RA and/or OA, *k* = 1; mixed diagnoses of RA or psoriatic arthritis, *k* = 1). The trials included 2751 patients with a mean age of 53.8 years (range of mean age of 10–68 years); mean disease duration was 9.7 years (range 2.3–19.0 years). Of the 30 trials with complete data on pain, 25 trials included a patient-reported outcome, and the remaining trials reported tender joint count (*k* = 2), Ritchie articular index (*k* = 1), and NSAID consumption (*k* = 2) (Table S2).

### 3.3. Risk of Bias in Included Studies

**Figure 2:**
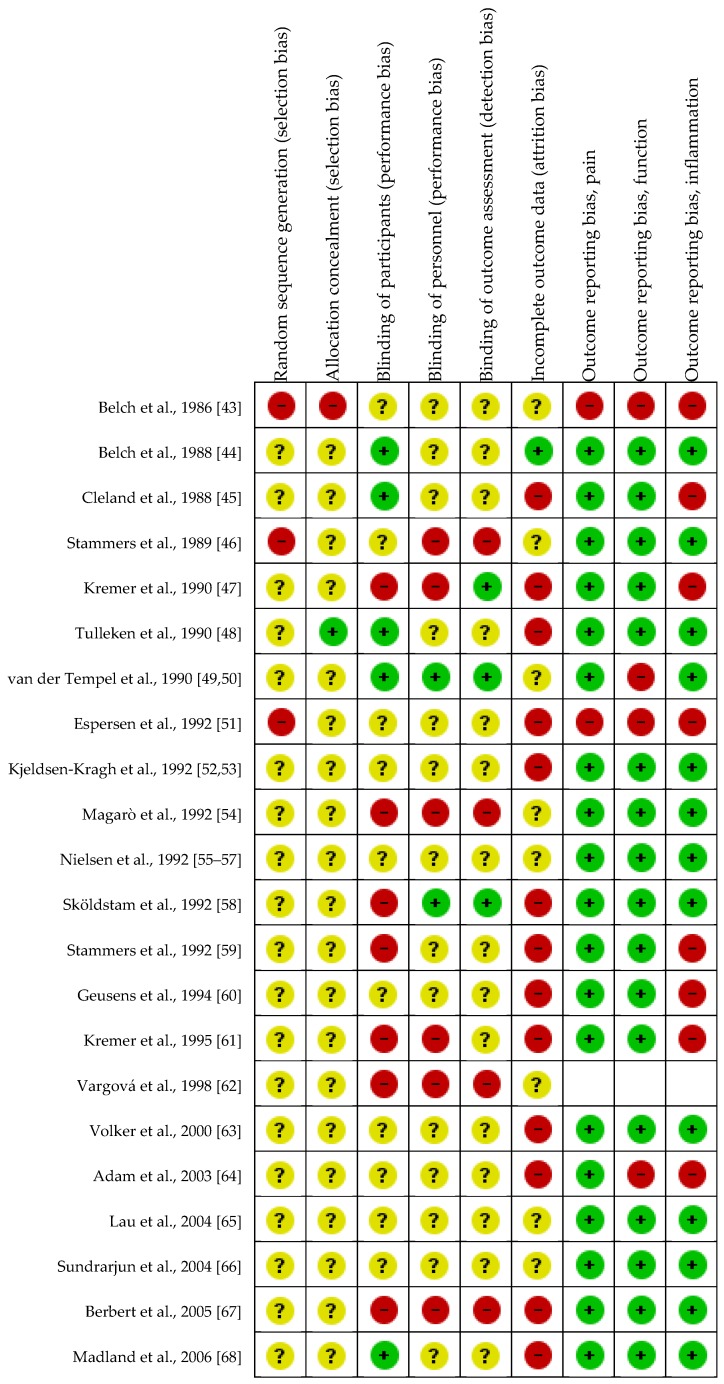

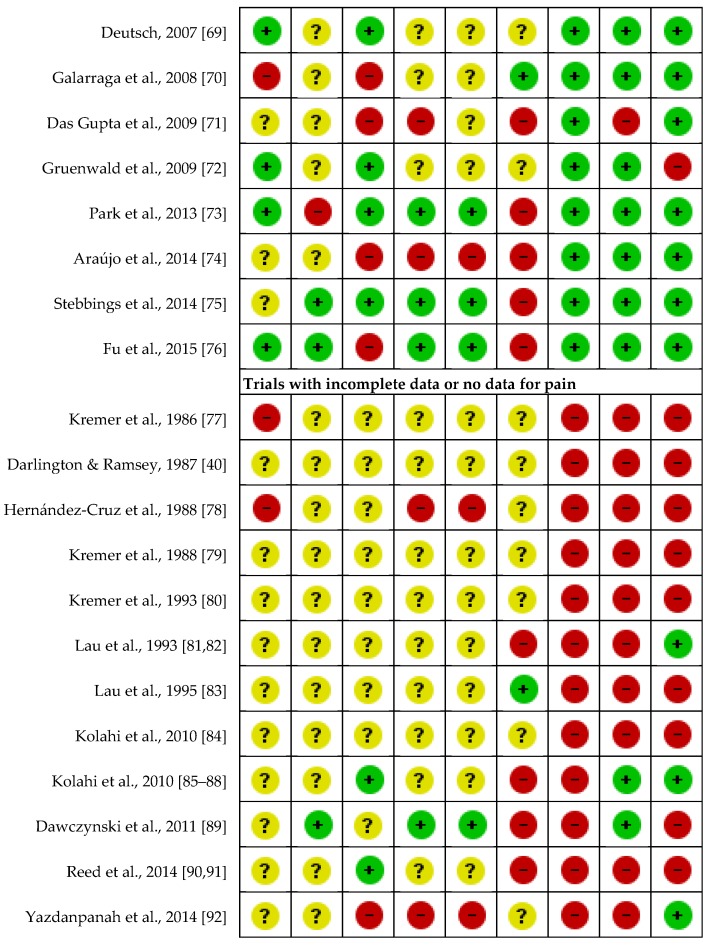
Risk of bias summary figure. 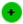: adequate methodology, 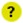: unclear methodology, 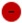: inadequate methodology. Risk of outcome reporting bias was not assessed for Vargová et al. (1998) [49], since only the abstract was available in English, and the rest of the article was not in English. Therefore, it was not considered appropriate to assess outcome reporting bias based only on the abstract of a full article.

No trials were judged as having low risk of bias in all nine domains (see Figure 2, bias assessment table, and outcome matrix in Table S3 and S4). Thirteen trials were judged as having high risk of pain outcome-reporting bias, 15 trials were judged to be at high risk of function outcome-reporting bias, and 18 trials were judged to be at high risk of inflammation outcome-reporting bias.

### 3.4. Primary Outcome: Pain

**Figure 3:**
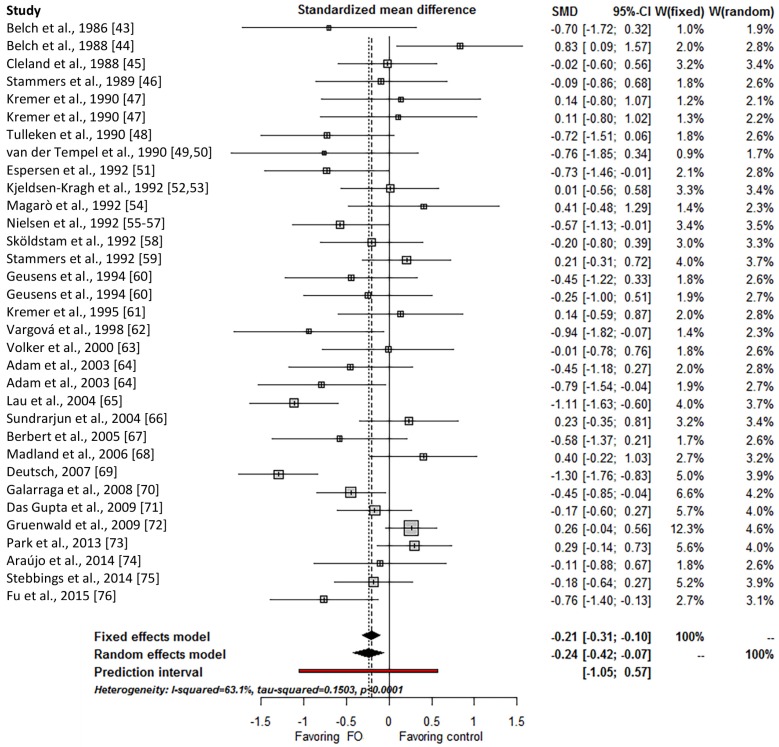
Forest plot of the effect size for marine oil interventions on pain. Weights are shown for both a random-effects model (“W(random)”) and a fixed-effects model (“W(fixed)”). 95% CI: 95% confidence interval; MO: marine oil; SMD: standardized mean differences. Note that the use of the term MO refers to all oils of marine origin (e.g., oil from whole fish and mussel oil).

Thirty trials presented complete data and were included in the meta-analysis on pain. The overall effect estimate corresponded to an SMD of −0.24 (95% confidence interval, CI, −0.42 to −0.07, *p* = 0.007), thus indicating a statistically significant pain reducing effect of marine oil (Figure 3). This result translates into an improvement of 8% on a VAS scale. However, not all studies suggested a beneficial effect of marine oil; hence, the effects were highly heterogeneous across studies (*I*^2^ = 63%) with a wide prediction interval for the overall effect estimate (−1.05–0.57).

Meta-regression analyses (Table 2), showed that a statistically significant amount of the heterogeneity could be explained by type of diagnosis (*p* = 0.024), supplementation type (*p* = 0.009), dosage of EPA plus DHA (*p* = 0.016), and ratio of EPA/DHA (*p* = 0.031), but not by type of control (*p* = 0.051) or duration (*p* = 0.074). There was a significant effect in patients with RA (22 trials; SMD −0.21; 95% CI, −0.42 to −0.00) and other/mixed diagnoses (3 trials; SMD −0.63; 95% CI, −1.20 to −0.06), but no effect in patients with OA (5 trials; SMD −0.17; 95% CI, −0.57–0.24). All bias domains, except blinding of personnel and blinding of outcome assessors, could also explain a significant amount of the heterogeneity. Post hoc meta-regression analyses for RA separately are provided in Table S5.

A significant positive association was found between SMD and total dose of EPA and DHA (slope β, 0.13 (g/day)^−1^, 95% CI, 0.04–0.22, *p* = 0.006), indicating less effect at higher dose, but there was no duration-response relationship (*p* = 0.568). Plots of the meta-regression analyses are provided in Figure S1.

A post hoc meta-regression analysis was performed exploring differences among the types of marine oil on pain, grouping types of oil as “whole fish” (*k* = 20), “mussel” (*k* = 3), “other” (*k* = 5, including cod liver oil, mixture of oil from whole fish and cod liver oil, krill oil, and seal oil), and “unspecified” (*k* = 3). The analysis showed a significant relationship between pain and type of marine oil (*p* = 0.012), where only the effect of mussel oil was statistically significant on its own, having a beneficial effect.

### 3.5. Secondary Outcomes: Inflammation and Function

A total of 23 and 25 trials provided complete data on function and inflammation, respectively. Meta-analysis showed no overall effect of marine oil on function (SMD −0.01; 95% CI, −0.19–0.18; *p* = 0.953) and moderate heterogeneity across studies (*I*^2^ = 60%). Including studies with incomplete data on function and a high risk of outcome-reporting bias, using null-imputations, yielded similar results (SMD −0.01; 95%CI, −0.13–0.10; *p* = 0.808).

The overall effect of marine oil on inflammation was significant (SMD −0.28; 95% CI, −0.51–0.06; *p* = 0.013), corresponding to an effect of –1.7 mg/dL (−3.0 to −0.4 mg/dL) CRP, with substantial heterogeneity across studies (*I*^2^ = 70%). Including studies with incomplete data on inflammation and a high risk of outcome-reporting bias yielded similar results (SMD −0.16; 95% CI, −0.29 to −0.02; *p* = 0.021). Forest plots for function and inflammation are provided in Figures S2 and S3, and meta-regression analyses for function and inflammation are provided in Tables S6 and S7.

### 3.6. Tolerance and Safety

A total of 28, 21, and 24 trials with complete data were included in the analysis of tolerance, withdrawals due to adverse events, and SAEs, respectively. For all three outcomes, there were no differences between the intervention and the control group, with an RR of 1.00 (95% CI, 0.96–1.03; *p* = 0.814), 0.82 (95% CI, 0.57–1.17; *p* = 0.279) and 0.75 (95% CI, 0.43–1.30; *p* = 0.308), respectively. Low heterogeneity was present for all three outcomes (*I*^2^ = 6.5%, 0%, and 0%, respectively).

### 3.7. Assessment of Reporting Bias

Visual inspection of the funnel plot for pain did not show any signs of asymmetry, and the result of the Egger test was nonsignificant (intercept α = −0.91; 95% CI, −2.75–0.93; *p* = 0.339). Similar results were found for function (intercept α = 0.83; 95% CI, −1.25–2.92; *p* = 0.442) and inflammation (intercept α = 0.06; 95% CI, −3.91–1.75, *p* = 0.462), but asymmetry was present for tolerance, withdrawals due to adverse events, and SAEs, likely due to outcome-reporting bias. The funnel plots are provided in Figures S4–S9.

Sensitivity analyses using fixed-effect models yielded similar results as the random-effects models for pain, function, and inflammation, indicating limited effect of potential small-study bias. Including trials with incomplete data on pain and a high risk of outcome-reporting bias yielded a slightly lower but still statistically significant effect (42 trials, SMD −0.16; 95% CI, −0.28 to −0.03; *p* = 0.012; forest plot is provided in Figure S10). The effect size was influenced by type of pain outcome, as shown by a post hoc meta-regression analysis (*p* = 0.005), with the effect size being nonsignificant for trials reporting patient-reported pain (SMD −0.18; 95% CI, −0.37–0.00; *p* = 0.051) and significant for trials reporting non-patient-reported pain (SMD −0.65; 95% CI, −0.65 to −0.16; *p* = 0.009). However, two of the latter trials were deemed low risk of outcome-reporting bias, and the effect on pain was still significant when excluding trials with both non-patient-reported pain outcome and high/unclear outcome-reporting bias (corresponding to ‘Adequate’ outcome reporting in Table 2).

The GRADE evidence profile with reasons for downgrading is provided in Table 3.

## 4. Discussion

The present systematic review and meta-analysis indicated an effect of marine oil on arthritis, although with a considerable heterogeneity across the studies. Twenty-two trials (N = 956) with complete data on pain assessed the effect of marine oil in patients with RA, for whom a favorable effect was seen, though the confidence in the estimate is considered moderate. Only five trials (N = 403) assessed the effect of marine oil in patients with OA, and only three trials (N = 150) assessed the effect in patients with other arthritis diagnoses; we rated the evidence to be of low grade for both patient groups. Hence, the evidence was not sufficiently robust to determine the effect of marine oil in patients with diagnoses other than RA. The results for the OA and other/mixed diagnosis groups were highly heterogeneous (*I*^2^ = 82% and 89%, respectively). Osteoarthritis has varying degrees of inflammation [4]; hence, in the future it might be appropriate to distinguish between OA with higher and lower degrees of inflammation.

A significant, beneficial effect on pain was found for marine oil with an EPA/DHA ratio >1.5, suggesting that EPA is more beneficial than DHA, which could be due to a more potent effect mechanism for EPA than for DHA [94]. An inverse dose-response relationship was found, suggesting less pain reduction with higher doses, which is against expectations. Previously, RA symptoms have been reduced at a total EPA and DHA dose of ≥2.6 g/day over ≥12 weeks, and the higher the doses the less time was needed to demonstrate the effect [95]. This was not supported by our findings, as a dose of ≥2.6 g/day was not significant in the meta-regression. However, the results should be interpreted with caution. This finding could be explained by a possible saturation dose-response-relationship, as different background diets provide varying amounts of EPA and DHA. One of the four trials applying the highest doses of EPA and DHA was conducted in a Norwegian population [52], which already has a high intake of fish oil compared to e.g., an American population [52], possibly making it more difficult to detect an effect. Two of the trials applying the highest doses reduced the patients’ intake of NSAIDs during the trial [47,52], which also might make it more difficult to detect an effect on pain. In addition, there is uncertainty about the actual doses ingested, since some of the trials included non-compliant patients and some did not measure compliance. The optimal type of marine oil could not be established because only few trials included marine oil from sources other than whole fish. Conclusions regarding dosage, duration, and ratio of EPA/DHA did not change by including only trials with RA patients.

Assessing types of adverse effects was beyond the scope of this systematic review, but no differences were found between intervention and control groups in terms of tolerance, withdrawals due to adverse events, and SAEs, which is in accordance with the findings of a systematic review of fish oil administration in older adults [96].

In general, the trials showed serious study limitations and a large degree of heterogeneity (i.e., “disagreement” with respect to the effect of marine oil). Heterogeneity was expected from pooling different diagnoses, types of marine oils, and measures within each outcome. The pooling, however, did increase the power of the analyses. When exploring the heterogeneity with meta-regression analyses, the majority of the heterogeneity remained unexplained for pain and inflammation, and quality of evidence for these two outcomes was therefore rated as low. When only trials with RA patients were included in a post hoc meta-analysis of pain, the heterogeneity was low (*I*^2^ = 32%), i.e., these trials generally agree on the effect. Post hoc analyses showed that heterogeneity for function was explained to a large degree by diagnosis and/or trial duration, and the quality of evidence was rated as moderate.

Poor reporting was a frequent issue in the trials, limiting their inclusion in the meta-analysis and making their methods nontransparent. Consequently, risk of bias may have been high, although it was frequently judged as unclear. Publication bias and outcome-reporting bias were also potential limitations. However, funnel plots, Egger tests, and sensitivity analyses including trials with incomplete data did not show evidence that these bias types were affecting the estimates for pain, function, or inflammation.

To our knowledge this is the first comprehensive systematic review and meta-analysis regarding the effect of marine oil on arthritis. In contrast to previous meta-analyses [14,15,16,17], we included all types of arthritis, all types of marine oil (i.e., oils of any marine origin), and did not include vegetable sources of *n*-3 PUFA (e.g., flaxseed oil). In addition, we applied extensive search strategies.

## 5. Conclusions

In conclusion, this meta-analysis suggests a small favorable effect of marine oil in reducing pain in patients with arthritis (SMD < −0.2) [97], but the evidence was of low quality. There is moderate quality evidence for an effect in RA patients. In contrast, the effect was statistically non-significant in patients with OA, but our confidence in the estimate is very low. Thus, more research is needed in order to provide evidence for firm conclusions regarding the effect of marine oil in OA and other types of arthritis, but also regarding the optimal dose, ratio of EPA and DHA, and which type of marine oil is preferable. If RA patients would like to try marine oil (i.e., oil from any marine origin), the results suggest a product with an EPA/DHA ratio >1.5, and there do not seem to be adverse effects.

## Figures and Tables

**Table 1:** Characteristics of the trials included in the meta-analyses trials presenting complete outcome data on pain are presented in the upper part of the panel.

Author, Year [Reference Number], (Trial Registry Number)	N Total	Study Design	Duration of Intervention (Weeks)	Diagnosis	Mean Age (Years)	% Females	Mean Disease Duration (Years)	No. of Patients Receiving Intervention	Intervention	Dose of EPA + DHA (g/Day)	No. of Patients Receiving Control	Control Treatment
**Trials with complete data for pain**												
Belch et al. 1986 [43]	34	Three-arm, parallel	52	RA	na	na	na	11 ^c^	MO (whole fish), and EPO (480 mg GLA)	0.24 + na	12 ^c^	Oil (unspecified) ^k^
											11 ^c^	EPO (540 mg GLA)
Belch et al. 1988 [44]	49	Three-arm, parallel	52	RA	49.0	29.3	5.0	15	MO (whole fish), and EPO (450 mg GLA)	0.24 + na	18	Paraffin ^k^
											16	EPO (540 mg GLA)
Cleland et al. 1988 [45]	60	Two-arm, parallel	12	RA	50.5	53.3	8.3	30	MO (whole fish)	3.20 + 2.00	30	Olive oil
Stammers et al. 1989 [46]	26	Two-arm, parallel	26	OA	52–84	80.8	na	na	MO (unspecified)	na	na	Oil (unspecified)
Kremer et al. 1990 [47]	64	Three-arm, parallel ^c^	24	RA	58.3	51.6	13.8	19	MO (whole fish), high dose	4.05 ^e^ + 2.70 ^e^	23	Olive oil ^j^
								22	MO (whole fish), low dose	2.03 ^e^ + 1.35 ^e^		
Tulleken et al. 1990 [48]	28	Two-arm, parallel	13	RA	55.0	85.7	18.0	14	MO (whole fish)	2.04 + 1.32	14	Coconut oil w/fish flavor
van der Tempel et al. 1990 [49,50]	16	Cross-over, no WO	12 ^a^	RA	53.0	56.3	12.0	16	MO (whole fish)	2.04 + 1.32	16	Coconut oil w/fish aroma
Espersen et al. 1992 [51]	32	Two-arm parallel	12	RA	na	na	na	18 ^c^	MO (whole fish)	2.00 + 1.20	14 ^c^	Mix (38% MUFA and 21% PUFA)
Kjeldsen-Kragh et al. 1992 [52,53]	79	Three-arm, parallel	16	RA	57.3	21.5	8.5	26	MO (whole fish), and declining NSAID	3.78 + 1.96	28	Corn oil and declining NSAID
								25	MO (whole fish), and NSAID ^k^	3.78 + 1.96		
Magarò et al. 1992 [54]	20	Two-arm, parallel	6	RA	25–45	100.0	na	10	MO (whole fish)	1.60 + 1.10	10	None
Nielsen et al. 1992 [55,56,57]	57	Two-arm, parallel	12	RA	61.0 ^b^	na	5.0 ^b^	29	MO (whole fish)	2.00 + 1.20	28	Mix of FA
Sköldstam et al. 1992 [58]	46	Two-arm, parallel	26	RA	57.0	73.9	18.0	23	MO (whole fish)	1.80 + 1.20	23	Maize oil, olive oil and peppermint oil
Stammers et al. 1992 [59]	86	Two-arm, parallel	24	OA	68.0	72.1	15.5	44	MO (cod liver oil)	0.79 + 0.70 ^g^	42	Olive oil
Geusens et al. 1994 [60]	90	Three-arm, parallel	52	RA	57.3	52.2	10.1	30	MO (whole fish), high dose	1.68 + 0.36	30	Olive oil ^j^
								30	MO (whole fish), low dose	0.84 + 0.18		
Kremer et al. 1995 [61]	66	Four-arm, parallel	26 or 30	RA	57.5	40.9	10.5	33 ^c^	MO (whole fish), diclofenac, and diclofenac placebo	4.29 ^e^ + 2.34 ^e^	33 ^c^	Corn oil, diclofenac, and diclofenac placebo
Vargová et al. 1998 [62]	23	Two-arm, parallel	21	JCA	10.1	na	na	13	“diet with increased content of omega-3 PUFA”	na	10	Unspecified
Volker et al. 2000 [63]	50	Two-arm, parallel	15	RA	57.0	n	13.5	25	MO (whole fish)	0.90 ^e,g^ +0.63 ^e,g^	25	50:50 corn oil and olive oil
Adam et al. 2003 [64]	68	Two parallel cross-over studies, 8.7 weeks. WO	13 ^a^	RA	56.8	41.2	9.6	34	MO (whole fish), and WD	0.80 ^e^ + 0.60 ^e^	34	Corn oil, and WD
					58.0	41.2	9.5	34	MO (whole fish), and AID	0.84 ^e^ + 0.63 ^e^	34	Corn oil, and AID
Lau et al. 2004 [65]	80	Two-arm, parallel	24	Knee OA	62.5	86.3	8.9	40	MO (GLM extract)	na	40	Olive oil
Sundrarjun et al. 2004 [66]	60	Three-arm, parallel	12	RA	46.9	85.0	4.4	23	MO (whole fish)	1.88 + 1.48	23	Unspecified
										na	14	None ^k^
Berbert et al. 2005 [67]	55	Three-arm, parallel	24	RA	49.0	61.8	15.3	18	MO (whole fish)	1.80 + 1.20	17	Soy oil
								20	MO (whole fish), and olive oil ^k^	1.80 + 1.20		
Madland et al. 2006 [68]	43	Two-arm, parallel	2	p.a. PsA	55.0	51.2	13.0 ^b^	22	MO (seal oil)	2.40 + 2.60	21	Soy oil (bottle)
Deutsch 2007 [69]	90	Two-arm, parallel	4	RA/CVD/OA	55.0	47.8	na	45	MO (krill oil)	0.05 + 0.03	45	Microcrystalline cellulose
Galarraga et al. 2008 [70]	97	Two-arm, parallel	36	RA	59.5	71.1	13.0	49	MO (mix of oil from cod liver and whole fish)	1.50 + 0.70	48	Air filled capsule
Das Gupta et al. 2009 [71]	100	Two-arm, parallel	12	RA	47.3	na	na	50	MO (whole fish), and indomethacin	1.80 ^g^ + 1.20 ^g^	50	Indomethacin
Gruenwald et al. 2009 [72] (EUCTR200500366918DE)	177	Two-arm, parallel	26	Knee/hip OA	62.3	63.8	na	90	MO (mix of oil from cod liver and whole fish), and glucosamine sulfate	0.60 ^i^	87	Mix of oils, and glucosamine sulfate
Park et al. 2013 [73] (NCT01618019)	109	Two-arm, parallel	16	RA	48.4	34.4	8.4	55	MO (whole fish)	2.09 + 1.17	54	Sunflower oil w/oleic acid
Araújo et al. 2014 [74]	37	Three-arm, parallel	26	RA	na	na	na	11 ^c^	MO (unspecified)	na	15 ^c^	na
								8	Mediterranean diet ^k^	na		
Stebbings et al. 2014 [75]	80	Two-arm, parallel	12	Knee or hip OA	66.4	55.0	na	39	MO (GLM)	0.01 +0.01	41	Corn oil
Fu et al. 2015 [76] (NCT02173587)	50	Two-arm, parallel	26	RA	57.5	60.0	7.6	25	50:50 MO (HMLE) and corn oil	0.07 ^f^ + 0.10 ^f^	25	Corn oil
**Trials with incomplete data or no data for pain**												
Kremer et al. 1986 [77]	36	Cross-over, 4 weeks WO	14 ^a^	RA	na	na	na	36	MO (whole fish)	2.70 + na	36	Unspecified
Darlington & Ramsey 1987 [40]	35	Two-arm, parallel	12	RA	na	na	na	(17) ^d^	MO (whole fish)	3.24 + 2.16	(18) ^d^	Olive oil
Hernández-Cruz et al. 1988 [78]	90	Two-arm, parallel	52	RA	43.2	89.0	3.4	45	MO (whole fish)	1.50 + na	45	Sunflower oil
Kremer et al. 1988 [79]	55	Three-arm, parallel	24	RA	na	na	na	(18) ^d^	MO (whole fish), high dose	4.05 ^e^ + 2.70 ^e^	(19) ^d^	Olive oil ^j^
								(18) ^d^	MO (whole fish), low dose	2.03 ^e^ + 1.35 ^e^		
Kremer et al. 1993 [80]	50	Two-arm, parallel	26 or 30	RA	na	na	na	(25) ^d^	MO (whole fish), diclofenac, and diclofenac placebo	9.75 ^e,h^	(25) ^d^	Corn oil, diclofenac, and diclofenac placebo
Lau et al. 1993 [81,82]	64	Two-arm, parallel	52	RA	51.4	70.3	4.2	32	MO (whole fish)	1.71 + 1.14	32	Air-filled capsule
Lau et al. 1995 [83]	45	Two-arm, parallel	26	RA	51.0 ^b^	71.1	2.3 ^b^	25	MO (whole fish)	1.70 + 1.10	20	Air-filled capsule
Kolahi et al. 2010 [84]	90	Three-arm, parallel	na	RA	na	Na	na	(30) ^d^	MO (whole fish), and vitamin E-placebo	na	(30) ^d^	MO placebo (unspecified) and vitamin E placebo
								(30) ^d^	MO (whole fish), and vitamin E ^k^			
Kolahi et al. 2010 [85,86,87,88] (IRCT138902073812N1)	90	Two-arm, parallel	13	RA	50.0 ^b^	92.2	4.6 ^b^	45	MO (whole fish)	0.18 + 0.12	45	Paraffin
Dawczynski et al. 2011 [89] (NCT01179971)	60	Four-arm, parallel	12	RA (or PsA)	56.2	71.7	na	15	MO (unspecified)	3.00 ^l^	15	Olive oil
								15	MO (unspecified), and GLA (1800 mg)	1.58 ^l^	15	GLA (3150 mg)
Reed et al. 2014 [90,91] (NCT00072982)	150	Three-arm, parallel	78	RA	59.2	81.3	8.6 ^b^	53	MO (whole fish), and borage seed oil	2.10 + 1.40	52	Borage seed oil, and sunflower seed oil
								45	MO (whole fish), and sunflower seed oil ^k^	2.10 + 1.40		
Yazdanpanah et al. 2014 [92] (IRCT2012102610799N2)	114	Six-arm, parallel	6	Knee OA	na	na	na	19	Omega-3, and acetaminophen and naproxen	1.00 ^h^	19	Acetaminophen and naproxen
								19	Omega-3 and acetaminophen	1.00 ^h^	19	Acetaminophen
								19	Omega-3 and naproxen	1.00 ^h^	19	Naproxen

AID: anti-inflammatory diet; CVD: cardiovascular disease; DHA: docosahexaenoic acid; EPA: eicosapentaenoic acid; EPO: evening primrose oil; FA: fatty acid; GLA: gamma-linolenic acid; GLM: green-lipped mussel; HMLE: hard-shelled mussel lipid extract; JCA: juvenile chronic arthritis; MO: marine oil; MUFA: monounsaturated fatty acid; N: number; na: not available; NSAID: nonsteroidal anti-inflammatory drugs, OA: osteoarthritis; p.a. PSA: polyarticular psoriatic arthritis; PUFA: polyunsaturated fatty acid; RA: rheumatoid arthritis; WD: western diet; WO: wash out; Note that the use of the term MO refers to all oils of marine origin (e.g., oil from whole fish, seal, or mussel); ^a^ Duration of first period of cross-over study; ^b^ Median; ^c^ The numbers of participants receiving the interventions are not clearly stated in the publication, but other information suggests the numbers reported; ^d^ No group sample sizes reported. Assumed equal distribution among groups; ^e^ Based on mean body weight of participants or, if not available, on 75 kg; ^f^ Mean dose given over 26 weeks; ^g^ Estimated from information about EPA/DHA ratio or content in similar products; ^h^ Dose of omega-3; ^i^ Dose of fish oil; ^j^ Placebo group used twice in meta-analysis for two comparisons. Number of patients in the control group divided by two in the analysis; ^k^ Group excluded from the meta-analysis.

**Table 2:** Results of meta-regression analyses. Analyzed using a random-effects restricted maximum of likelihood (REML)-based meta-regression model.

Variable	Total Trials, *k*	SMD for Pain	95% CI	τ^2^	*I*^2^	*p*-Value for Interaction
**All trials**	30	−0.24	(−0.42 to −0.07)	0.150	63%	n.a.
**Diagnoses**				0.147	60%	0.024 ^a^
RA	22	−0.21	(−0.42 to −0.00) ^a^			
OA	5	−0.16	(−0.57 to 0.24)			
Other	3	−0.63	(−1.20 to −0.06) ^a^			
**Supplementation type**				0.137	60%	0.009 ^a^
Capsule	23	−0.25	(−0.44 to −0.06) ^a^			
Bottle	3	0.19	(−0.37 to 0.75)			
Unclear	4	−0.61	(−1.16 to −0.05) ^a^			
**Type of control**				0.148	60%	0.051
PUFA w/o EPA and DHA	11	−0.12	(−0.41 to 0.17)			
Non-PUFA oils	10	−0.30	(−0.59 to −0.01) ^a^			
Non-oils	3	−0.63	(−1.13 to −0.12) ^a^			
Unclear	3	−0.01	(−0.59 to 0.56)			
None	3	−0.21	(−0.87 to 0.44)			
**Duration**				0.165	63%	0.074
<12 weeks	3	−0.30	(−0.89 to 0.30)			
≥12 weeks and <24 weeks	12	−0.25	(−0.53 to 0.03)			
≥24 weeks	15	−0.23	(−0.48 to 0.03)			
Unspecified	0					
**Ratio of EPA/DHA**				0.153	61%	0.031 ^a^
Ratio of EPA/DHA ≤1.5	12	−0.12	(−0.40 to 0.15)			
Ratio of EPA/DHA of >1.5	11	−0.38	(−0.67 to −0.10) ^a^			
Unspecified	7	−0.23	(−0.62 to 0.17)			
**Dosage of EPA plus DHA**				0.138	58%	0.016 ^a^
<2.6 g/day	8	−0.45	(−0.75 to −0.15) ^a^			
≥2.6 g/day and <3.6 g/day	11	−0.21	(−0.51 to 0.09)			
≥3.6 g/day	4	0.13	(−0.32 to 0.57)			
Unspecified	7	−0.22	(−0.60 to 0.16)			
**Source of marine oil**				0.130	57%	0.011 ^a^
Whole fish	19	−0.17	(−0.38 to 0.05)			
Mussel	2	−0.95	(−1.60 to −0.31) ^a^			
Other	6	−0.19	(−1.53 to 0.16)			
Unspecified	3	−0.35	(−0.97 to 0.26)			
**Bias domains**						
**Random sequence generation (selection bias)**				0.160	61%	0.043 ^a^
Adequate	4	−0.33	(−0.78 to 0.13)			
Unclear	22	−0.19	(−0.40 to 0.02)			
Inadequate	4	−0.48	(−1.00 to 0.05)			
**Allocation concealment (selection bias)**				0.155	61%	0.039 ^a^
Adequate	3	−0.52	(−1.09 to 0.05)			
Unclear	25	−0.22	(−0.42 to −0.03) ^a^			
Inadequate	2	−0.03	(−0.76 to 0.70)			
**Blinding of participants (performance bias)**				0.149	61%	0.026 ^a^
Adequate	9	−0.10	(−0.42 to 0.22)			
Unclear	10	−0.41	(−0.70 to −0.11) ^a^			
Inadequate	11	−0.20	(−0.49 to 0.10)			
**Blinding of personnel (performance bias)**				0.163	63%	0.060
Adequate	5	−0.25	(−0.70 to 0.20)			
Unclear	17	−0.29	(−0.52 to −0.05) ^a^			
Inadequate	8	−0.13	(−0.50 to 0.24)			
**Incomplete outcome data (attrition bias)**				0.152	60%	0.020 ^a^
Adequate	2	0.06	(−0.61 to 0.73)			
Unclear	10	−0.44	(−0.77 to −0.12) ^a^			
Inadequate	18	−0.18	(−0.40 to 0.04)			
**Outcome reporting (outcome reporting bias for pain)**				0.145	61%	0.016 ^a^
Adequate	27	−0.20	(−0.38 to −0.02) ^a^			
Unclear	1	−0.94	(−2.10 to 0.21)			
Inadequate	2	−0.72	(−1.52 to 0.08)			
**Funding source**				0.149	61%	0.031 ^a^
Industry only	8	−0.10	(−0.41 to 0.21)			
Mixed	8	−0.10	(−0.44 to 0.25)			
Nonprofit only	4	−0.41	(−0.87 to 0.05)			
Not reported	7	−0.31	(−0.70 to 0.08)			
Unclear	3	−0.80	(−1.42 to −0.16) ^a^			

95% CI: 95% confidence interval, *I*^2^: heterogeneity, SMD: standardized mean difference, k: number of trials, τ^2^: estimated between-study variance; ^a^ *p* < 0.05.

**Table 3:** GRADE evidence profile.

Quality Assessment						No. of Patients		Effect		Quality
No. of Trials	Risk of Bias	Inconsistency	Indirectness	Imprecision	Other Considerations	MO	Non-MO	Relative (95% CI)	Absolute (95% CI)	
Pain										
30	Serious (−1) ^a^	Serious (−1) ^b^	Not serious	Not serious	None	781	728	-	SMD 0.24 lower (0.42 lower to 0.07 lower)	⨁⨁◯◯LOW
RA 22	Serious (−1) ^a^	Not serious	Not serious	Not serious	None	499	457	-	SMD 0.21 lower (0.42 lower to −0.004 lower)	⨁⨁⨁◯MODERATE
OA 5	Serious (−1) ^a^	Serious (−1) ^b^	Not serious	Serious (−1) ^d^	None	205	198	-	SMD 0.16 lower (0.57 lower to 0.24 higher)	⨁◯◯◯VERY LOW
Other 3	Serious (−1) ^a^	Serious (−1) ^b^	Serious (−1) ^c^	Serious (−1) ^d^	None	77	73	-	SMD 0.63 lower (1.20 lower to −0.06 lower) ^†^	⨁◯◯◯VERY LOW
Function (assessed with functional tests)										
23	Serious (−1) ^a^	Not serious	Not serious	Not serious	None	666	611	-	SMD 0.01 lower (0.19 lower to 0.18 higher)	⨁⨁⨁◯MODERATE
Inflammation										
25	Serious (−1) ^a^	Serious (−1) ^b^	Not serious	Not serious	None	581	573	-	SMD 0.28 lower (0.51 lower to 0.06 lower)	⨁⨁◯◯LOW
Tolerance										
28	Serious (−1) ^a^	Not serious	Not serious	Not serious	None	951	899	**RR 1.00** (0.96 to 1.03)	3 fewer per 1000 (from 27 fewer to 22 more) ^e^	⨁⨁⨁◯MODERATE
Number of withdrawals due to adverse events										
21	Serious (−1) ^a^	Not serious	Not serious	Not serious	None	751	691	**RR 0.82** (0.57 to 1.17)	16 fewer per 1000 (from 36 fewer to 15 more) ^e^	⨁⨁⨁◯MODERATE
Serious adverse events										
24	Serious (−1) ^a^	Not serious	Not serious	Serious (−1) ^d^	None	890	839	**RR 0.75** (0.43 to 1.30)	8 fewer per 1000 (from 19 fewer to 10 more) ^e^	⨁⨁◯◯LOW

Table made with GRADEpro, (Computer program on www.gradepro.org), version July 8, 2015. McMaster University, 2014; 95% CI: 95% confidence interval, MO: marine oil, OA: osteoarthritis, RA: rheumatoid arthritis, RR: relative risk, SMD: standardized mean difference. Note that the use of the term MO refers to all oils of marine origin (e.g., oil from whole fish, seal, or mussel); ^a^ The major study limitations were not using intention-to-treat, and unclear random sequence generation, allocation concealment, blinding of personnel, blinding of outcome assessment in most of the trials. None of the trials were rated adequate for all bias domains, with the maximum being five out of seven for pain, function and inflammation. For the “other” group, the maximum was three out of seven. For tolerance, number of withdrawals due to adverse events, and serious adverse events, the maximum was four out of six; ^b^ Unexplained heterogeneity and wide prediction intervals. For pain, a maximum of 13% of the heterogeneity could be explained by the meta-regression analyses. For inflammation, a maximum of 29% of the heterogeneity could be explained. In contrast, a maximum of 86% of the heterogeneity could be explained for function. When doing separate analyses according to type of arthritis, the heterogeneity was 32%, 82%, and 89% for RA, OA and “other”, respectively. For the two latter, too few trials were available to investigate the heterogeneity; ^c^ Indirect evidence, from including an intervention of “diet with increased content of omega-3 PUFA” [62]; ^d^ Wide 95% confidence interval, i.e., including both harmful and beneficial effects. Trials including patients with OA and “other” included only 403 and 150 patients, respectively, which is below the calculated optimal information size (OIS) of 786 patients; ^e^ Calculated by 1000 ACR∙(1 − RR), where ACR is the assumed control risk, calculated by (number of events in control group)/(size of control group).

## Supplementary Materials

The following are available online at http://www.mdpi.com/2072-6643/9/1/42/s1, Section S1: Reference Lists of Included Trials in the Meta-Analysis, Section S2: Reference Lists of Excluded Trials prior to the Systematic Review, Section S3: Reference Lists of Excluded Trials prior to the Meta-Analysis, Table S1: Search Strategies for all Databases, Table S2: Outcome Matrix, Table S3: Bias Assessment Table, Table S4: Outcome reporting bias Assessment, Table S5: Meta-Regression Analysis on Pain for Rheumatoid Arthritis Patients, Table S6: Meta-Regression Analysis on Function, Table S7: Meta-Regression Analysis on Inflammation, Figure S1: Meta-regression analysis (SMDs plotted against EPA plus DHA and duration of treatment), Figure S2: Forest Plot for Function, Figure S3: Forest Plot for Inflammation, Figure S4: Funnel Plot for Pain, Figure S5: Funnel Plot for Function, Figure S6: Funnel Plot for Inflammation, Figure S7: Funnel Plot for Tolerance, Figure S8: Funnel Plot for Withdrawals due to Adverse Events, Figure S9: Funnel Plot for Serious Adverse Events, Figure S10: Forest Plot for Pain Including Trials with Incomplete Data, Figure S11: Forest Plot for Function Including Trials with Incomplete Data, Figure S12: Forest Plot for Inflammation Including Trials with Incomplete Data.
