# Supplementary material for: Marine Oil Supplements for Arthritis Pain: A Systematic Review and Meta-Analysis of Randomized Trials
Source: Nutrients. 2017 Jan 6;9(1):42. doi: 10.3390/nu9010042 (PMC5295086; doi:10.3390/nu9010042)
Supplement: Supplementary file 1 [file nutrients-09-00042-s001.docx]

Supplementary Materials: Marine Oil Supplements for Arthritis Pain: A Systematic Review and
Meta-Analysis of Randomized Trials

Ninna K. Senftleber, Sabrina M. Nielsen, Jens R. Andersen, Henning Bliddal, Simon Tarp,
Lotte Lauritzen, Daniel E. Furst, Maria E. Suarez-Almazor, Anne Lyddiatt and Robin Christensen

[Section S1. Reference lists of included trials in the meta-analysis](#_Toc467939584) 2

[Section S2. Reference lists of excluded trials prior to the systematic review](#_Toc467939585) 4

[Section S3. Reference lists of excluded trials prior to the meta-analysis](#_Toc467939586) 9

[Table S1. Search strategies for all databases 1](#_Toc467939587)1

[Table S2. Outcome matrix](#_Toc467939588) 19

[Table S3. Bias assessment table 2](#_Toc467939589)9

[Table S4. Outcome reporting bias assessment 4](#_Toc467939590)9

[Table S5. Meta-regression analysis on pain for rheumatoid arthritis patients 5](#_Toc467939591)5

[Table S6. Meta-regression analysis on function 5](#_Toc467939592)6

[Table S7. Meta-regression analysis on inflammation 5](#_Toc467939593)7

[Figure S1. Meta-regression analysis (SMDs plotted against EPA plus DHA and duration of treatment) 5](#_Toc467939594)8

[Figure S2. Forest plot for function 5](#_Toc467939595)9

[Figure S3. Forest plot for inflammation](#_Toc467939596) 60

[Figure S4. Funnel plot for pain](#_Toc467939597) 60

[Figure S5. Funnel plot for function](#_Toc467939598) 61

[Figure S6. Funnel plot for inflammation 6](#_Toc467939599)1

[Figure S7. Funnel plot for tolerance 6](#_Toc467939600)1

[Figure S8. Funnel plot for withdrawals due to adverse events 6](#_Toc467939601)2

[Figure S9. Funnel plot for serious adverse events 6](#_Toc467939602)2

[Figure S10. Forest plot for pain including trials with incomplete data 6](#_Toc467939603)3

[Figure S11. Forest plot for function including trials with incomplete data 6](#_Toc467939604)4

[Figure S12. Forest plot for inflammation including trials with incomplete data 6](#_Toc467939605)5

S1. Reference Lists of Included Trials in the Meta-Analysis (42 Trials, 51 Records)

S1.1. Trials with Complete Data or No Data for Pain

1. Belch, J.J.F.; Ansell, D.; Madhok, R.; Sturrock, R.D. The effect of evening primrose oil epo and epo-fish oil combination on rheumatoid arthritis a double blind study. *Br. J. Rheumatol*. **1986**, *25*, 75.
2. Belch, J.J.; Ansell, D.; Madhok, R.; O’Dowd, A.; Sturrock, R.D. Effects of altering dietary essential fatty acids on requirements for non-steroidal anti-inflammatory drugs in patients with rheumatoid arthritis: a double blind placebo controlled study. *Ann. Rheum. Dis*. **1988**, *47*, 96–104.
3. Cleland, L.G.; French, J.K.; Betts, W.H.; Murphy, G.A.; Elliott, M.J. Clinical and biochemical effects of dietary fish oil supplements in rheumatoid arthritis. *J. Rheumatol*. **1988**, *15*, 1471–1475.
4. Stammers, T.; Sibbald, B.; Freeling, P. Fish oil in osteoarthritis. *Lancet* **1989**, *2*, 503.
5. Kremer, J.M.; Lawrence, D.A.; Jubiz, W.; DiGiacomo, R.; Rynes, R.; Bartholomew, L.E.; Sherman, M. Dietary fish oil and olive oil supplementation in patients with rheumatoid arthritis. Clinical and immunologic effects. *Arthritis Rheum*. **1990**, *33*, 810–820.
6. Tulleken, J.E.; Limburg, P.C.; Muskiet, F.A.; van Rijswijk, M.H. Vitamin E status during dietary fish oil supplementation in rheumatoid arthritis. *Arthritis Rheum*. **1990**, *33*, 1416–1419.
7. Van der Tempel, H.; Tulleken, J.E.; Limburg, P.C.; Muskiet, F.A.; van Rijswijk, M.H. Effects of fish oil supplementation in rheumatoid arthritis. *Ann. Rheum. Dis*. **1990**, *49*, 76–80.
8. Tulleken, J.E.; Limburg, P.; Vanrijswijk, M.H. Fish oil and plasma-fibrinogen. *BMJ* **1988**, *297*, 615–615.
9. Espersen, G.T.; Grunnet, N.; Lervang, H.H.; Nielsen, G.L.; Thomsen, B.S.; Faarvang, K.L.; Dyerberg, J.; Ernst, E. Decreased interleukin-1 beta levels in plasma from rheumatoid arthritis patients after dietary supplementation with *n*-3 polyunsaturated fatty acids. *Clin. Rheumatol*. **1992**, *11*, 393–395.
10. Kjeldsen-Kragh, J.; Lund, J.A.; Riise, T.; Finnanger, B.; Haaland, K.; Finstad, R.; Mikkelsen, K.; Førre, O. Dietary omega-3 fatty acid supplementation and naproxen treatment in patients with rheumatoid arthritis. *J. Rheumatol*. **1992**, *19*, 1531–1536.
11. Kjeldsen-Kragh, J. Dietary treatment of rheumatoid arthritis. *Scand. J. Rheumatol*. **1996**, *25*, 63–63.
12. Magaro, M.; Zoli, A.; Altomonte, L.; De Sole, P.; Di Mario, G.; De Leo, E. Effect of fish oil on neutrophil chemiluminescence induced by different stimuli in patients with rheumatoid arthritis. *Ann. Rheum. Dis*. **1992**, *51*, 877–880.
13. Nielsen, G.L.; Faarvang, K.L.; Thomsen, B.S.; Teglbjærg, K.L.; Jensen, L.T.; Hansen, T.M.; Lervang, H.H.; Schmidt, E.B.; Dyerberg, J.; Ernst, E. The effects of dietary supplementation with *n*-3 polyunsaturated fatty acids in patients with rheumatoid arthritis: a randomized, double blind trial. *Eur. J. Clin. Investig.* **1992**, *22*, 687–691.
14. Faarvang, K.L.; Nielsen, G.L.; Thomsen, B.S.; Teglbjaerg, K.L.; Hansen, T.M.; Lervang, H.H.; Schmidt, E.B.; Dyerberg, J.; Ernst, E. [Fish oils and rheumatoid arthritis. A randomized and double-blind study]. *Ugeskr Laeger*. **1994**, *156*, 3495–3498.
15. Nielsen, G.L.; Faarvang, K.L.; Thomsen, B.S.; Teglibjaerg, K.L.; Ernst, E. Effects of supplementation with *n*-3 fatty acids on clinical disease variables in patients with rheumatoid arthritis. *Eur. J. Clin. Investig*. **1991**, *21*, 67.
16. Sköldstam, L.; Börjesson, O.; Kjällman, A.; Seiving, B.; Åkesson, B. Effect of six months of fish oil supplementation in stable rheumatoid arthritis. A double-blind, controlled study. *Scand. J. Rheumatol*. **1992**, *21*, 178–185.
17. Stammers, T.; Sibbald, B.; Freeling, P. Efficacy of cod liver oil as an adjunct to non-steroidal anti-inflammatory drug treatment in the management of osteoarthritis in general practice. *Ann. Rheum. Dis*. **1992**, *51*, 128–129.
18. Geusens, P.; Wouters, C.; Nijs, J.; Jiang, Y.; Dequeker, J. Long-term effect of omega-3 fatty acid supplementation in active rheumatoid arthritis. A 12-month, double-blind, controlled study. *Arthritis Rheum*. **1994**, *37*, 824–829.
19. Kremer, J.M.; Lawrence, D.A.; Petrillo, G.F.; Litts, L.L.; Mullaly, P.M.; Rynes, R.I.; Stocker, R.P.; Parhami, N.; Greenstein, N.S.; Fuchs, B.R.; et al. Effects of high-dose fish oil on rheumatoid arthritis after stopping nonsteroidal antiinflammatory drugs. Clinical and immune correlates. *Arthritis Rheum*. **1995**, *38*, 1107–1114.
20. Vargová, V.; Vesely, R.; Sasinka, M.; Torok, C. [Will administration of omega-3 unsaturated fatty acids reduce the use of nonsteroidal antirheumatic agents in children with chronic juvenile arthritis?] *Cas Lek Cesk.* **1998**, *137*, 651–653.
21. Volker, D.; Fitzgerald, P.; Major, G.; Garg, M. Efficacy of fish oil concentrate in the treatment of rheumatoid arthritis. *J. Rheumatol*. **2000**, *27*, 2343–2346.
22. Adam, O.; Beringer, C.; Kless, T.; Lemmen, C.; Adam, A.; Wiseman, M.; Adam, P.; Klimmek, R.; Forth, W. Anti-inflammatory effects of a low arachidonic acid diet and fish oil in patients with rheumatoid arthritis. *Rheumatol. Int*. **2003**, *23*, 27–36.
23. Lau, C.S.; Chiu, P.K.Y.; Chu, E.M.Y.; Cheng, I.Y.W.; Tang, W.M.; Man, R.Y.K.; Halpern, G.M. Treatment of knee osteoarthritis with Lyprinol, lipid extract of the green-lipped mussel—A double-blind placebo-controlled study. *Progress Nutr.* **2004**, *6*, 17–31.
24. Sundrarjun, T.; Komindr, S.; Archararit, N.; Dahlan, W.; Puchaiwatananon, O.; Angthararak, S.; Udomsuppayakul, U.; Chuncharunee, S. Effects of *n*-3 fatty acids on serum interleukin-6, tumour necrosis factor-alpha and soluble tumour necrosis factor receptor p55 in active rheumatoid arthritis. *J. Int. Med. Res*. **2004**, *32*, 443–454.
25. Berbert, A.A.; Kondo, C.R.; Almendra, C.L.; Matsuo, T.; Dichi, I. Supplementation of fish oil and olive oil in patients with rheumatoid arthritis. *Nutrition* **2005**, *21*, 131–136.
26. Madland, T.M.; Bjorkkjaer, T.; Brunborg, L.A.; Froyland, L.; Berstad, A.; Brun, J.G. Subjective improvement in patients with psoriatic arthritis after short-term oral treatment with seal oil. A pilot study with double blind comparison to soy oil. *J. Rheumatol*. **2006**, *33*, 307–310.
27. Deutsch, L. Evaluation of the effect of Neptune Krill Oil on chronic inflammation and arthritic symptoms. *J. Am. Coll. Nutr*. **2007**, *26*, 39–48.
28. Galarraga, B.; Ho, M.; Youssef, H.M.; Hill, A.; McMahon, H.; Hall, C.; Ogston, S.; Nuki, G.; Belch, J.J. Cod liver oil (*n*-3 fatty acids) as an non-steroidal anti-inflammatory drug sparing agent in rheumatoid arthritis. *Rheumatology* **2008**, *47*, 665–669.
29. Das Gupta, A.B.; Hossain, A.K.; Islam, M.H.; Dey, S.R.; Khan, A.L. Role of omega-3 fatty acid supplementation with indomethacin in suppression of disease activity in rheumatoid arthritis. *Bangladesh Med. Res. Counc. Bull.* **2009**, *35*, 63–68.
30. Gruenwald, J.; Petzold, E.; Busch, R.; Petzold, H.P.; Graubaum, H.J. Effect of glucosamine sulfate with or without omega-3 fatty acids in patients with osteoarthritis. *Adv. Ther*. **2009**, *26*, 858–871.
31. Park, Y.; Lee, A.; Shim, S.C.; Lee, J.H.; Choe, J.Y.; Ahn, H.; Choi, CB.; Sung, YK.; Bae, SC. Effect of *n*-3 polyunsaturated fatty acid supplementation in patients with rheumatoid arthritis: A 16-week randomized, double-blind, placebo-controlled, parallel-design multicenter study in Korea. *J. Nutr. Biochem*. **2013**, *24*, 1367–1372.
32. Araújo, C.A.; Moraes-Fontes, M.F.; Santos, L.; Riso, N. Omega-3 fatty acids and Mediterranean diet as complimentary therapies for rheumatoid arthritis. *Arthritis Rheumatol*. **2014**, *66*, S1050.
33. Stebbings, S.; Gray, A.; Schneiders, A.; Sansom, A. SAT0446 A novel green-lipped mussel lipid extract (biolex) for the treatment of pain in osteoarthritis of the hip and knee: A randomized double-blind, placebo controlled trial. *Ann Rheum Dis*. **2014**, *73*, doi:10.1136/annrheumdis-2014-eular.3398.
34. Fu, Y.; Li, G.; Zhang, X.; Fu, Y.; Li, G.; Zhang, X.; Xing, G.; Hu, X.; Yang, L.; Li, D. Lipid Extract from hard-shelled mussel (mytilus coruscus) improves clinical conditions of patients with rheumatoid arthritis: A randomized controlled trial. *Nutrients* **2015**, *7*, 625–645.

S1.2. Trials with incomplete data or no data for pain

1. Kremer, J.M.; Rynes, R.I.; Bartholomew, L.E.; Michalek, A.; Jubiz, W. A double-blinded placebo controlled crossover study of eicosapentaenoic-acid supplementation in active rheumatoid arthritis. *Arthritis Rheum*. **1986**, *29*, S11.
2. Darlington, L.G.; Ramsey, N.W. Olive oil for rheumatoid patients? *Br. J. Rheumatol*. **1987**, *26*, 129.
3. Hernández-Cruz, B.; Alcocer-Varela, J.; Cardiel, M.H. Omega-3 fatty acids supplementation in Mexican patients with rheumatoid arthritis with standard treatment. A blinded, randomized, placebo controlled, one year, clinical trial. *Arthritis Rheum*. **1998**, *41*, S155.
4. Kremer, J.; Larence, D.; Jubiz, W.; Digarcomo, R.; Rynes, R.; Bartholomew, L. Different doses of fish-oil fatty acid supplementation in rheumatoid arthritis ra a prospective double-blinded randomized study. *Arthritis Rheum.* **1988**, *31*, S303.
5. Kremer, J.M.; Lawrence, D.A.; Stocker, R.S.; Rynes, R.I.; Parhami, N.; Greenstein, N.S.; Petrillo, G.F.; Simon, L.S.; Robinson, D.W. Clinical, immunological and nsaid sparing effects of fish-oil (FO) in patients with active rheumatoid-arthritis (RA)—A long-term prospective randomized double-blind-study. *Arthritis Rheum*. **1993**, *36*, S56.
6. Lau, C.S.; Morley, K.D.; Belch, J.J.F. Effects of fish-oil supplementation on nonsteroidal antiinflammatory drug requirement in patients with mild rheumatoid-arthritis—A double-blind placebo-controlled study. *Br. J. Rheumatol*. **1993**, *32*, 982–989.
7. Lau, C.S.; McMahon, H.; Morley, K.D.; Belch, J.J.F. Effects of Maxepa on non-steroidal anti-inflammatory drug usage in patients with mild rheumatoid arthritis. *Br. J. Rheumatol*. **1991**, *30*, 137.
8. Lau, C.S.; McLaren, M.; Belch, J.J. Effects of fish oil on plasma fibrinolysis in patients with mild rheumatoid arthritis. *Clin. Exp. Rheumatol*. **1995**, *13*, 87–90.
9. Kolahi, S.O.; Hejasi, J.A.; Mohtadinia, A. The effect of fish oil and vitamin E supplement over lipid peroxidation and antioxidant levels of plasma in patient with rheumatoid arthritis. *Int. J. Rheum Dis*. **2010**, *13*, 108.
10. Kolahi, S.; Ghorbanihaghjo, A.; Alizadeh, S.; Rashtchizadeh, N.; Argani, H.; Khabazzi, A.R.; Hajialilo, M.; Bahreini, E. Fish oil supplementation decreases serum soluble receptor activator of nuclear factor-kappa B ligand/osteoprotegerin ratio in female patients with rheumatoid arthritis. *Clin. Biochem*. **2010**, *43*, 576–580.
11. Ghorbanihaghjo, A.; Kolahi, S.; Seifirad, S.; Rashtchizadeh, N.; Argani, H.; Hajialilo, M.; Khabazi, A.; Alizadeh, S.; Bahreini, E. Effect of fish oil supplements on serum paraoxonase activity in female patients with rheumatoid arthritis: a double-blind randomized controlled trial. *Arch Iran Med*. **2012**, *15*, 549–552.
12. Kolahi, S.; Haghjoo, A.G. Effect of Fish Oil supplements on serum paraoxonase activity in patients with rheumatoid arthritis. *Rheumatology* **2012**, *51*, i31.
13. Alizadeh, S.; Ghorbanihaghjo, A.; Kolahi, S.; Bahreini, E. Effect of omega-3 fatty acids supplementation on circulating osteoprotegerin ligand to osteoprotegerin ratio in female patients with rheumatoid arthritis. *Clin. Biochem*. **2011**, *1*, S142.
14. Dawczynski, C.; Hackermeier, U.; Viehweger, M.; Stange, R.; Springer, M.; Jahreis, G. Incorporation of *n*-3 PUFA and gamma-linolenic acid in blood lipids and red blood cell lipids together with their influence on disease activity in patients with chronic inflammatory arthritis—A randomized controlled human intervention trial. *Lipids Health Dis*. **2011**, *10*, 130.
15. Reed, G.W.; Leung, K.; Rossetti, R.G.; Vanbuskirk, S.; Sharp, J.T.; Zurier, R.B. Treatment of rheumatoid arthritis with marine and botanical oils: an 18-month, randomized, and double-blind trial. *Evid. Based Complement. Alternat. Med*. **2014**, *2014*, 857456.
16. Olendzki, B.C.; Leung, K.; Van Buskirk, S.; Reed, G.; Zurier, R.B. Treatment of rheumatoid arthritis with marine and botanical oils: Influence on serum lipids. *Evid. Based Complement. Alternat. Med*. **2011**, *2011*, 827286.
17. Yazdanpanah, P.; Mohamadi, H.; Sadeghi, H.; Ghaffarian Shirazi, H.; Arjmand, A. Survey of omega-3 effects on morning stiffness in osteoarthritis of the knee. *Pain Pract*. **2014**, *14*, 126.

S2. Reference Lists of Trials Excluded Prior to the Systematic Review

S2.1. Not Primary Research (Reviews, Letters etc.)

1. Bell, R.F.; Borzan, J.; Kalso, E.; Simonnet, G. Food, pain, and drugs: Does it matter what pain patients eat? *Pain* **2012**, *153*, 1993–1996.

2. Uhlig, T. [Omega-3 fatty acids (fish oil) in the clinical use]. *Deutsch. Med. Wochenschr.* **1995**, *120*, 1262–1263.

3. Schmidt, K.L. Treatment of rheumatoid-arthritis in the year 2000. *Internist* **1993**, *34*, 831–840.

4. Robin, J.L. [Potential value of eicosapentaenoic acid]. *Allergy Immunol.* **1987**, *19* (Suppl. 8), 12–13.

5. Hornstra, G. Fish oil supplements. [Dutch]. *Geneesmiddelenbulletin* **1999**, *33*, 37–42.

6. Baumann, A.; Hagenlocher, Y.; Lorentz, A. Nutrition and Immunology. *Ernahrungs Umsch.* **2013**, *60*, M706–M716.

7. Ballabio, C.B.; Fantini, F. [Inflammatory aspects and therapeutic possibilities of rheumatic diseases. Introduction]. *Minerva Med.* **1973**, *64*, 2385–2394.

8. Halpern, G.M. [An anti-inflammatory from the South Seas: Lyprinol]. *Allergy Immunol.* **2000**, *32*, 259–260.

9. Niklas, H. [Prostaglandins and therapy for rheumatism]. *Med. Monatsschr. Pharm.* **1979**, *2*, 368–369.

10. Podell, R.N. Nutritional treatment of rheumatoid arthritis. Can alterations in fat intake affect disease course? *Postgrad. Med.* **1985**, *77*, 68–69.

11. Reinhold, U. Specific application of single nutrients as a basic treatment in immunostimulating therapy. *Med. Hypotheses* **1987**, *22*, 159–169.

12. Neame, R.L.; Doherty, M. Managing osteoarthritis. *Practitioner* **2003**, *247*, 768–770.

13. Miller, C.A. Newer and safer options for osteoarthritis. *Geriatr. Nurs.* **2001**, *22*, 165–166.

14. Robinson, D.R. Alleviation of autoimmune-disease by dietary lipids containing omega-3-fatty-acids. *Rheum. Dis. Clin. N. Am.* **1991**, *17*, 213–222.

15. Calder, P.C. Marine omega-3 polyunsaturated fatty acids and rheumatoid arthritis. In *Bioactive Food as Dietary Interventions for Arthritis and Related Inflammatory Diseases: Bioactive Food in Chronic Disease States*, Academic Press: San Diego, CA, USA, 2013.

16. Proudman, S.; Cleland, L.; James, M. Omega-3 fatty acids for early rheumatoid arthritis. *Med. Today* **2007**, *8*, 57–59.

17. Merry, P.; Kidd, B.; Blake, D. Modification of rheumatic symptoms by diet and drugs. *Proc. Nutr. Soc.* **1989**, *48*, 363–369.

18. Watson, J.; Madhok, R.; Wijelath, E.; Capell, H.A.; Gillespie, J.; Smith, J.; Byars, M.L. Mechanism of action of polyunsaturated fatty acids in rheumatoid arthritis. *Biochem. Soc. Trans.* **1990**, *18*, 284–285.

19. Suvarna, B.S. Secrets of omega-3 oil. *Kathmandu Univ. Med. J.* **2008**, *6*, 406–411.

20. Schwarzenfeld, C.; Haindl, P.M.; Rintelen, B.; Leeb, B.F. Polyunsaturated fatty acids and rheumatoid arthritis. *Curr. Top. Nutraceutical Res.* **2009**, *7*, 45–49.

21. Schoenherr, W.D.; Jewell, D.E. Nutritional modification of inflammatory diseases. *Semin. Vet. Med. Surg. Small Anim.* **1997**, *12*, 212–222.

22. Sanders, T.A.B. Marine oils—Metabolic effects and role in human-nutrition. *Proc. Nutr. Soc.* **1993**, *52*, 457–472.

23. Robinson, D.R.; Xu, L.L.; Knoell, C.T.; Tateno, S.; Guo, M.; Colvin, R.B.; Olesiak, W.; Urakaze, M.; Sugiyama, E.; Auron, P.E. Alleviation of autoimmune disease by omega 3 fatty acids. *World Rev. Nutr. Diet.* **1994**, *76*, 95–102.

24. Ramsbottom, H.; Lockwood, G.B. Nutraceuticals for healthy joints. *Pharm. J.* **2006**, *277*, 740–746.

25. Pascoe, P.J. Alternative methods for the control of pain. *J. Am. Vet. Med. Assoc.* **2002**, *221*, 222–229.

26. Panush, R.S. Nutritional therapy for rheumatic diseases. *Ann. Intern. Med.* **1987**, *106*, 619–621.

27. McCann, K. Nutrition and rheumatoid arthritis. *Explore* **2007**, *3*, 616–618.

28. March, L.M.; Stenmark, J. Non-pharmacological approaches to managing arthritis. *Med. J. Aust.* **2001**, *175*, S102–S107.

29. Gibson, R.A. The effect of diets containing fish and fish oils on disease risk-factors in humans. *Aust. N. Z. J. Med.* **1988**, *18*, 713–722.

30. Cleland, L.G.; James, M.J. Osteoarthritis. Omega-3 fatty acids and synovitis in osteoarthritic knees. *Nat. Rev. Rheumatol.* **2012**, *8*, 314–315.

31. Belluzzi, A. *N*-3 and *n*-6 fatty acids for the treatment of autoimmune diseases. *Eur. J. Lipid Sci. Technol.* **2001**, *103*, 399–407.

32. Belch, J.J.F.; Muir, A. *N*-6 and *n*-3 essential fatty acids in rheumatoid arthritis and other rheumatic conditions. *Proc. Nutr. Soc.* **1998**, *57*, 563–569.

33. Anonymous. Fish oil. *Altern. Med. Rev.* **2000**, *5*, 576–580.

34. Bhatnagar, D.; Hussain, F. Omega-3 fatty acid ethyl esters (Omacor (R)) for the treatment of hypertriglyceridemia. *Future Lipidol.* **2007**, *2*, 263–270.

35. Kjeldsen-Kragh, J. Mediterranean diet intervention in rheumatoid arthritis. *Ann. Rheum. Dis.* **2003**, *62*, 193–195.

36. Glickman-Simon, R.; Geller, J. Tai chi, myofascial release, tetanic electrical stimulation, omega-3 fatty acid supplementation, antioxidant supplements. *Explore* **2012**, *8*, 252–255.

37. Daviglus, M.; Sheeshka, J.; Murkin, E. Health benefits from eating fish. *Comments Toxicol.* **2002**, *8*, 345–374.

38. Choi, H.K. Diet and rheumatoid arthritis: Red meat and beyond. *Arthritis Rheum.* **2004**, *50*, 3745–3747.

39. Brzezinski, A. Review. *Gastroenterol. Hepatol.* **2007**, *3*, 787–788.

40. Bengmark, S. Nutritional modulation of acute- and ‘chronic’-phase responses. *Nutrition* **2001**, *17*, 489–495.

41. Anonymous. Drugs for osteoarthritis. *Med. Lett. Drugs Ther.* **2014**, *56*, 80–84.

42. Hagen, K.B.; Byfuglien, M.; Olsen, S.; Smedslund, G. Dietary interventions for rheumatoid arthritis. *Cochrane Database of Syst. Rev.* **2007**, *1*, doi:10.1002/14651858.CD006400.pub2.

43. Anonymous. Wanted: fewer side effects. *Health News* **2004**, *10*, 7.

44. Scherak, O.; Kolarz, G. Vitamin E and rheumatoid arthritis. *Arthritis Rheum.* **1991**, *34*, 1205–1206.

45. Zimmerman, K.F.; Schlesinger, P.A.; Bloss, T.J.; Stillman, M.T. Fish-oil supplementation and rheumatoid arthritis. *Ann. Intern. Med.* **1987**, *107*, 262–263.

46. Harrison, R.A.; Harrison, B.J. Fish oils are beneficial to patients with established rheumatoid arthritis. *The J. Rheumatol.* **2001**, *28*, 2563–2565.

47. Murray, F.E. Fish oils in rheumatoid arthritis. *Lancet* **1987**, *2*, 1157–1158.

48. Machtey, I. Vitamin E in arthritis. *Arthritis Rheum.* **1991**, *34*, 1205–1206.

49. Kyriakopoulos, A.A. Rheumatoid arthritis and fish oil. *Ann. Intern. Med.* **1987**, *107*, 941.

50. Hart, F.D.; Karmali, R.A.; Birtwistle, S.; McEwen, L.M. Dietary fatty acids and rheumatoid arthritis. *Lancet* **1985**, *1*, 699–700.

51. Kremer, J.M. Omega-3 fatty acids in rheumatoid arthritis. *Del. Med. J.* **1988**, *60*, 679–681.

52. Lau, C.S. Collateral benefits of fish oil therapy for rheumatoid arthritis. *J. Rheumatol.* **2006**, *33*, 1931–1933.

53. Rollins, C.J. Effect of intravenous omega-3 fatty acids on clinical symptoms of rheumatoid arthritis. *JPEN. J. Parenter. Ente. Nutr.* **2010**, *34*, 169–170.

54. Pike, M.C. Antiinflammatory effects of dietary lipid modification. *J. Rheumatol.* **1989**, *16*, 718–720.

55. Cleland, L.G.; James, M.J. Fish oil and rheumatoid arthritis: Antiinflammatory and collateral health benefits. *J. Rheumatol.* **2000**, *27*, 2305–2307.

56. Cleland, L.G.; James, M.J. Rheumatoid arthritis and the balance of dietary *n*-6 and *n*-3 essential fatty acids. *Br. J. Rheumatol.* **1997**, *36*, 513–514.

57. Jacobs, J.W.G.; Rasker, J.J.; Bijlsma, J.W.J. Alternative medicine in rheumatology: Threat or challenge? *Clin. Exp. Rheumatol.* **2001**, *19*, 117–119.

58. Tulleken, J.E.; van Rijswijk, M.H. [Fish oil in the treatment of patients with rheumatoid arthritis]. *Nederlands Tijdschr. Geneeskd.* **1988**, *132*, 1875–1877.

59. Quesenberry, P.J.; Butera, J.N. An interesting fishing expedition. *Cancer Biol. Ther.* **2009**, *8*, 338–339.

60. Munk, P.L. Musculoskeletal intervention: Thinking outside the box. *J. Vasc. Interv. Radiol* **2013**, *24*, 793–794.

61. Vasey, F.B.; Carter, J.D. Intestinal flora and psoriatic arthritis. *J. Rheumatol.* **2006**, *33*, 2099.

62. Keysser, G. Nutrition in Disease of rheumatic Morphogenetic Region. *Ernahrungs Umschau* **2014**, *61*, M610–M617.

63. Lerman, R.H. Essential fatty acids. *Altern. Ther. Health Med.* **2006**, *12*, 20–29.

64. Leak, A.M. Advances in the treatment of rheumatic diseases. *Practitioner* **1983**, *227*, 1139–1145.

65. DeLuca, P.; Rothman, D.; Zurier, R.B. Marine and botanical lipids as immunomodulatory and therapeutic agents in the treatment of rheumatoid arthritis. *Rheum. Dis. Clin. N. Am.* **1995**, *21*, 759–777.

S2.2. Non-Randomized

1. Srivastava, P. Therapeutic application of cyanobacterium spirulina in the treatment of rheumatoid arthritis. *Phycologia* **2009**, *48*, 124–124.

2. Honzlova, M.; Peliskova, Z.; Trnavsky, K. [Initial experience with the treatment of rheumatoid arthritis by dietary manipulation]. *Casopís Lékar̆ů c̆Eských* **1989**, *128*, 149–152.

3. Gruenwald, J.; Graubaum, H.J.; Harde, A. Effect of cod liver oil on symptoms of rheumatoid arthritis. *Adv. Ther.* **2002**, *19*, 101–107.

4. Cleland, L.G.; Gibson, R.A.; Neumann, M.; French, J.K. The effect of dietary fish oil supplement upon the content of dihomo-gammalinolenic acid in human plasma phospholipids. *Prostaglandins Leukot. Essent. Fat. Acids* **1990**, *40*, 9–12.

5. Cho, S.H.; Jung, Y.B.; Seong, S.C.; Park, H.B.; Byun, K.Y.; Lee, D.C.; Song, E.K.; Son, J.H. Clinical efficacy and safety of Lyprinol, a patented extract from New Zealand green-lipped mussel (Perna Canaliculus) in patients with osteoarthritis of the hip and knee: A multicenter 2-month clinical trial. *Eur. Ann. Allergy Clin. Immunol.* **2003**, *35*, 212–216.

6. Gheita, T.; Kamel, S.; Helmy, N.; El-Laithy, N.; Monir, A. Omega-3 fatty acids in juvenile idiopathic arthritis: effect on cytokines (IL-1 and TNF-alpha), disease activity and response criteria. *Clin. Rheumatol.* **2012**, *31*, 363–366.

7. Proudman, S.M.; Keen, H.I.; Stamp, L.K.; Lee, A.T.; Goldblatt, F.; Ayres, O.C.; Rischmueller, M.; James, M.J.; Hill, C.L.; Caughey, G.E.; Cleland, L.G. Response-driven combination therapy with conventional disease-modifying antirheumatic drugs can achieve high response rates in early rheumatoid arthritis with minimal glucocorticoid and nonsteroidal anti-inflammatory drug use. *Semin. Arthritis Rheum.* **2007**, *37*, 99–111.

8. Lassus, A.; Dahlgren, A.L.; Halpern, M.J.; Santalahti, J.; Happonen, H.P. Effects of dietary supplementation with polyunsaturated ethyl ester lipids (Angiosan) in patients with psoriasis and psoriatic arthritis. *J. Int. Med. Res.* **1990**, *18*, 68–73.

9. Kumar Gupta, V.; Khan Zafer, Z.Y.; Ahmad, M. The concomitant consumption of cod liver oil causes a reduction in the daily Diclofenac Sodium usage in rheumatoid arthritis patients: A pilot study. *J. Clin. Diagn. Res.* **2013**, *7*, 1347–1351.

10. Gruenwald, J.; Graubaum, H.J.; Hansen, K.; Grube, B. Efficacy and tolerability of a combination of Lyprinol and high concentrations of EPA and DHA in inflammatory rheumatoid disorders. *Adv. Ther.* **2004**, *21*, 197–201.

11. Caughey, G.E.; James, M.J.; Proudman, S.M.; Cleland, L.G. Fish oil supplementation increases the cyclooxygenase inhibitory activity of paracetamol in rheumatoid arthritis patients. *Complement. Ther. Med.* **2010**, *18*, 171–174.

12. Cleland, L.G.; Caughey, G.E.; James, M.J.; Proudman, S.M. Reduction of cardiovascular risk factors with longterm fish oil treatment in early rheumatoid arthritis. *J. Rheumatol.* **2006**, *33*, 1973–1979.

13. Kremer, J.M.; Jubiz, W.; Michalek, A. Fish-oil fatty acid supplementation in active rheumatoid arthritis. A double-blinded, controlled, crossover study. *Ann. Intern. Med.* **1987**, *106*, 497–503.

14. Villard, G.; Delacretaz, J. [Reiter’s disease in the female]. *Schweizerische Med. Wochenschr.* **1974**, *104*, 1228–1231.

15. Gibson, S.L. The effect of a lipid extract of the New Zealand green-lipped mussel in three cases of arthritis. *J. Altern. Complement. Med.* **2000**, *6*, 351–354.

S2.3. Noninterventional

1. Pritchett, J.W. Statins and dietary fish oils improve lipid composition in bone marrow and joints. *Clin. Orthop. Relat. Res.* **2007**, *456*, 233–237.

2. Lau, C.; Gallacher, C.; Ross, P.; Belch, J. Rheumatoid arthritis: fish oil? Or snake oil? *Br. J. Rheumatol.* **1991**, *30*, 72–73.

S2.4. Irrelevant Intervention

1. Zhou, X.; Zhou, Z.; Jin, M. [Clinical and experimental study on effect of Shuguan granule on mid-late rheumatoid arthritis]. *Chin. J. Integr. Tradit. West. Med.* **1999**, *19*, 80–83.

2. Antoniuk, M.V.; Magalish, T.L. [Efficacy of sodium gumate electrophoresis in rheumatoid arthritis]. *Vopr. Kurortol. Fizioter. Lech. Fiz. Kult.* **2005**, *4*, 20–22.

3. Kadir, A.A.; Ab Wahab, S.Z.; Zulkifli, M.M.; Noor, N.M.; Baie, S.B.B.; Haron, J. The therapeutic effect of oral Channa striatus extract on primary knee osteoarthritis patients. *Agro Food Ind. Hi-Tech* **2014**, *25*, 44–48.

4. Skoldstam, L.; Berglund, U.; Eriksson, A.; Akesson, B. [A diet rich in polyunsaturated fatty acids is of no help in patients with rheumatoid arthritis]. *Läkartidningen* **1988**, *85*, 4411–4415.

5. Berezhnyts’kyi, M.M.; Virstiuk, N.H.; Makarchuk, O.S. [Changes in the unsaturated fatty acids and lipid peroxidation during the treatment of patients with rheumatoid arthritis]. *Likars’ka Sprava* **1996**, *3–4*, 53–56.

6. Catanzaro, R.; Lorenzetti, A.; Solimene, U.; Zerbinati, N.; Milazzo, M.; Celep, G.; Sapienza, C.; Italia, A.; Polimeni, A.; Marotta, F. Testing a novel bioactive marine nutraceutical on osteoarthritis patients. *Acta Bio-Med.* **2013**, *84*, 30–37.

7. Hansen, G.; Nielsen, L.; Kluger, E.; Thysen, M.H.; Emmertsen, H.; Stengård-Pedersen, K.; Lund, E.C.; Unger, B.; Andersen, P.W. [Nutritional status of Danish patients with rheumatoid arthritis and effects of a diet adjusted in energy intake, fish content and antioxidants]. *Ugeskr Laeger* **1998**, *160*, 3074–3078.

8. Hansen, G.V.; Nielsen, L.; Kluger, E.; Thysen, M.; Emmertsen, H.; Stengaard-Pedersen, K.; Hansen, E.L.; Unger, B.; Andersen, P.W. Nutritional status of Danish rheumatoid arthritis patients and effects of a diet adjusted in energy intake, fish-meal, and antioxidants. *Scand. J. Rheumatol.* **1996**, *25*, 325–330.

9. Sarzi-Puttini, P.; Comi, D.; Boccassini, L.; Muzzupappa, S.; Turiel, M.; Panni, B.; Salvaggio, A. Diet therapy for rheumatoid arthritis. A controlled double-blind study of two different dietary regimens. *Scand. J. Rheumatol.* **2000**, *29*, 302–307.

10. Denissov, L.N.; Sharafetdinov, K.K.; Samsonov, M.A. On the medicinal efficacy of dietetic therapy in patients with rheumatoid-arthritis. *Int. J. Clin. Pharmacol. Res.* **1992**, *12*, 19–25.

S2.5. Non-Arthritis

1. Jones, D.W.; Wright, D.; Jankowski, T.A.; Vincent, E.C. Q/what treatments relieve arthritis and fatigue associated with systemic lupus erythematosus? *J. Fam. Pract.* **2014**, *63*, 607–617.

2. Frati, C.; Bevilacqua, L.; Apostolico, V. Association of etretinate and fish oil in psoriasis therapy. Inhibition of hypertriglyceridemia resulting from retinoid therapy after fish oil supplementation. *Acta Derm. Venereol. Suppl.* **1994**, *186*, 151–153.

3. Fontani, G.; Suman, A.L.; Migliorini, S.; Corradeschi, F.; Ceccarelli, I.; Aloisi, A.; Carli, G. Administration of omega-3 fatty acids reduces positive tender point count in chronic musculoskeletal pain patients. *J. Complement. Integr. Med.* **2010**, *7*, doi:10.2202/1553-3840.1366.

4. Fahrer, H.; Hoeflin, F.; Lauterburg, B.H.; Peheim, E.; Levy, A.; Vischer, T.L. Diet and fatty acids: can fish substitute for fish oil? *Clin. Exp. Rheumatol.* **1991**, *9*, 403–406.

5. Mantzioris, E.; James, M.J.; Gibson, R.A.; Cleland, L.G. Dietary substitution with an alpha-linolenic acid-rich vegetable oil increases eicosapentaenoic acid concentrations in tissues. *Am. J. Clin. Nutr.* **1994**, *59*, 1304–1309.

6. Lindh, G.; Ronneberg, R.; Karlsson, J. Plasma nutrients in joint and muscle pain patients before and after nutritional therapy. *J. Nutr. Environ. Med.* **1997**, *7*, 15–26.

S2.6. Not Add-On

1. Kremer, J.M.; Bigauoette, J.; Michalek, A.V.; Timchalk, M.A.; Lininger, L.; Rynes, R.I.; Huyck, C.; Zieminski, J.; Bartholomew, L.E. Effects of manipulation of dietary fatty acids on clinical manifestations of rheumatoid arthritis. *Lancet* **1985**, *1*, 184–187.

2. Caturla, N.; Funes, L.; Perez-Fons, L.; Micol, V. A randomized, double-blinded, placebo-controlled study of the effect of a combination of lemon verbena extract and fish oil omega-3 fatty acid on joint management. *J. Altern. Complement. Med.* **2011**, *17*, 1051–1063.

3. Moore, N.; Pariente, A. Effect of a food supplement on NSAIDs use in osteoarthritis of the knee or hip: A double-blind randomized placebo-controlled clinical trial. *Basic Clin. Pharmacol. Toxicol.* **2009**, *105*, 125.

4. Dawczynski, C.; Schubert, R.; Hein, G.; Müller, A.; Eidner, T.; Vogelsang, H.; Basu, S.; Jahreis, G. Long-term moderate intervention with *n*-3 long-chain PUFA-supplemented dairy products: effects on pathophysiological biomarkers in patients with rheumatoid arthritis. *Br. J. Nutr.* **2009**, *101*, 1517–1526.

5. Magaro, M.; Altomonte, L.; Zoli, A.; Mirone, L.; De Sole, P.; Di Mario, G.; Lippa, S.; Oradei, A. Influence of diet with different lipid composition on neutrophil chemiluminescence and disease activity in patients with rheumatoid arthritis. *Ann. Rheum. Dis.* **1988**, *47*, 793–796.

6. Kayacelebi, A.A.; Willers, J.; Pham, V.V.; Hahn, A.; Schneider, J.Y.; Rothmann, S.; Frölich, J.C.; Tsikas, D. Plasma homoarginine, arginine, asymmetric dimethylarginine and total homocysteine interrelationships in rheumatoid arthritis, coronary artery disease and peripheral artery occlusion disease. *Amino Acids* **2015**, *47*, 1885–1891.

7. Remans, P.H.; Sont, J.K.; Wagenaar, L.W.; Wouters-Wesseling, W.; Zuijderduin, W.M.; Jongma, A.; Breedveld, F.C.; Van Laar, J.M. Nutrient supplementation with polyunsaturated fatty acids and micronutrients in rheumatoid arthritis: Clinical and biochemical effects. *Eur. J. Clin. Nutr.* **2004**, *58*, 839–845.

8. Gibson, S.L.M.; Gibson, R.G. The treatment of arthritis with a lipid extract of Perna canaliculus: A randomized trial. *Complement. Ther. Med.* **1998**, *6*, 122–126.

9. Jacquet, A.; Girodet, P.O.; Pariente, A.; Forest, K.; Mallet, L.; Moore, N. Phytalgic, a food supplement, vs placebo in patients with osteoarthritis of the knee or hip: A randomised double-blind placebo-controlled clinical trial. *Arthritis Res. Ther.* **2009**, *11*, R192.

10. Torley, H.I.; Richards, I.M.; O’Dowd, A.; Fitzsimmons, C.; Belch, J.J.F.; Sturrock, R.D. A double blind placebo controlled trial of efamol marine in psoriatic arthritis. *Br. J. Rheumatol.* **1989**, *28* (Suppl. 2), 1.

11. Zawadzki, M.; Janosch, C.; Szechinski, J. Perna canaliculus lipid complex PCSO-524 demonstrated pain relief for osteoarthritis patients benchmarked against fish oil, a randomized trial, without placebo control. *Mar. Drugs* **2013**, *11*, 1920–1935.

12. Szechinski, J.; Zawadzki, M. Measurement of pain relief resulting from the administration of Perna canaliculus lipid complex PCSO-524 (TM) as compared to fish oil for treating patients who suffer from osteoarthritis of knee and/or hip joints. *Reumatologia* **2011**, *49*, 244–252.

13. Heinle, K.; Adam, A.; Gradl, M.; Wiseman, M.; Adam, O. [Selenium concentration in erythrocytes of patients with rheumatoid arthritis. Clinical and laboratory chemistry infection markers during administration of selenium]. *Med. Klin.* **1997**, *92* (Suppl. 3), 29–31.

14. Hamblin, L.; Laird, A.; Parkes, E.; Walker, A.F. Improved arthritic knee health in a pilot RCT of phytotherapy. *J. R. Soc. Promot. Health* **2008**, *128*, 255–262.

15. Veale, D.J.; Torley, H.I.; Richards, I.M.; O'Dowd, A.; Fitzsimons, C.; Belch, J.J.; Sturrock, R.D. A double-blind placebo controlled trial of Efamol Marine on skin and joint symptoms of psoriatic arthritis. *Br. J. Rheumatol.* **1994**, *33*, 954–958.

S2.7. Animal Studies

1. Lefkowith, J.B.; Morrison, A.; Lee, V.; Rogers, M. Manipulation of the acute inflammatory response by dietary polyunsaturated fatty-acid modulation. *J. Immunol.* **1990**, *145*, 1523–1529.

S3. Reference Lists of Trials Excluded Prior to the Meta-Analysis

S3.1. No Data Available (8 Trials, 8 Records)

S3.1.1. Only trial registration available (7 trials, 7 records)

1. ClinicalTrials.gov-Registration. ClinicalTrials.gov. Bethesda (MD): National Library of Medicine (US). 2000 February 29. Identifier NCT01351805, Vitamin D and Fish Oil for Autoimmune Disease, Inflammation and Knee Pain. Available online: http://ClinicalTrials.gov/show/NCT01351805 (accessed on 13 May 2015).

2. ClinicalTrials.gov-Registration. ClinicalTrials.gov. Bethesda (MD): National Library of Medicine (US). 2000 February 29. Identifier NCT00399282, Omega-3 and Vitamin E Supplementation in Patients with Rheumatoid Arthritis. Available online: http://ClinicalTrials.gov/show/NCT00399282 (accessed on 13 May 2015).

3. ClinicalTrials.gov-Registration. ClinicalTrials.gov. Bethesda (MD): National Library of Medicine (US). 2000 February 29. Identifier NCT01818804, The Effect of *n-*3 Polyunsaturated Fatty Acids in Patients with Psoriatic Arthritis. Available online: http://ClinicalTrials.gov/show/NCT01818804 (accessed on 13 May 2015).

4. ClinicalTrials.gov-Registration. ClinicalTrials.gov. Bethesda (MD): National Library of Medicine (US). 2000 February 29. Identifier NCT02333084, The Influence Of a Natural Anti-Inflammatory Product on Levels of Inflammatory Markers in Cases with Osteoarthritis of Knee. Available online: http://ClinicalTrials.gov/
show/NCT02333084 (accessed on 13 May 2015).

5. ClinicalTrials.gov-Registration. ClinicalTrials.gov. Bethesda (MD): National Library of Medicine (US). 2000 February 29. Identifier NCT01742468, Intervention With *n-*3 Polyunsaturated Fatty Acids in Patients with Rheumatoid Arthritis. Available online: http://ClinicalTrials.gov/show/NCT01742468 (accessed on 13 May 2015).

6. CTRI-Registration. Clinical Trials Registry India. New Delhi: Database Publisher (India). 2007 June 20. Identifier CTRI/2010/091/000059, Evaluation of Efficacy and Tolerability of a Fixed Dose Combination of Glucosamine Sulphate and Omega 3 Fatty Acid For Treatment of Painful Osteoarthritis of knee: An Open, Prospective, Randomized Three Arm Study. Available online: http://www.ctri.nic.in/Clinicaltrials/
pmaindet2012.php?trialid=1278 (accessed on 13 May 2015).

7. UMIN-CTR-Registration. UMIN Clinical Trial Registry. Japan, University Hospital Medical Information Network (UMIN) Center: 1989. Identifier JPRN-UMIN000014413, Effects of Krill Oil on Knee Joint Pain. Available online: https://upload.umin.ac.jp/cgi-open-bin/ctr/ctr.cgi?function=brows&action=brows&type=
summary&recptno=R000016768&language=E (accessed on 13 May 2015).

S3.1.2. Ongoing at Publication Date (1 Trial, 1 Record)

1. Singh, G.; Chandra, R.K. Fish oils in rheumatoid arthritis. *Ann. Intern. Med.* **1988**, *108*, 904–905.

S3.1.3. Language (5 Trials, 5 Records)

1. Alpigiani, M.G.; Ravera, G.; Buzzanca, C.; Devescovi, R.; Fiore, P.; Iester, A. [The use of *n-*3 fatty acids in chronic juvenile arthritis]. *Med. Surg. Pediatr.* **1996**, *18*, 387–390.

2. Tchorzewski, H.; Banasik, M.; Glowacka, E.; Lewkowicz, P. [Modification of innate immunity in humans by active components of shark liver oil]. *Polski Merkur. Lekarsk.* **2002**, *13*, 329–332.

3. Adam, O. [Nutrition as adjuvant therapy in chronic polyarthritis]. *Z. Rheumatol.* **1993**, *52*, 275–280.

4. Astorga, G.; Cubillos, A.; Masson, L.; Silva, J.J. [Active rheumatoid arthritis: effect of dietary supplementation with omega-3 oils. A controlled double-blind trial]. *Rev. Med. Chile* **1991**, *119*, 267–272.

5. Mizushima, Y. [Progress on therapy of collagen diseases and rheumatoid arthritis]. *Nihon Naika Gakkai Zasshi* **1993**, *82*, 386–389.

S3.1.4. High vs. Low Dose (4 Trials, 8 Records)

1. Sundström, B.; Stålnacke, K.; Hagfors, L.; Johansson, G. Supplementation of omega-3 fatty acids in patients with ankylosing spondylitis. *Scand. J. Rheumatol.* **2006**, *35*, 359–362.

2. ANZCTR-Registration. Australian and New Zealand Clinical Trials Registry. Sydney (NSW): National Health and Medical Research Council (Australia); 2005—Identifier ACTRN12607000415404, A Randomized Controlled Trial in Adults with Knee Osteoarthritis to Determine the Effect of High Dose Fish Oil Compared with Low Dose Fish Oil on Symptomatic and Structural Outcomes. Available online: http://www.anzctr.org.au/ACTRN12607000415404.aspx (accessed on 12 May 2015).

3. Proudman, S.; Spargo, L.; Hall, C.; McWilliams, L.; Lee, A.; Rischmueller, M.; Gibson, R.; James, M.; Cleland, L. Fish oil in rheumatoid arthritis: a randomised, double blind trial comparing high dose with low dose. *Intern. Med. J.* **2012**, *42*, 2–3.

4. Proudman, S.; Spargo, L.; Hall, C.; McWilliams, L.; Lee, A.; Rischmueller, M.; Gibson, R.; James, M.; Cleland, L. Fish Oil in Rheumatoid Arthritis: A Randomised, Double Blind Trial Comparing High Dose with Low Dose. *Arthritis Rheum.* **2012**, *64*, S196–S196.

5. Denman, M. In patients with early rheumatoid arthritis, fish oil reduced failure of treatment with DMARDs. *Ann. Intern. Med.* **2014**, *160*, doi:10.7326/0003-4819-160-4-201402180-02011.

6. Proudman, S.M.; James, M.J.; Spargo, L.D.; Metcalf, R.G.; Sullivan, T.R.; Rischmueller, M.; Flabouris, K.; Wechalekar, M.D.; Lee, A.T.; Cleland, L.G. Fish oil in recent onset rheumatoid arthritis: a randomised, double-blind controlled trial within algorithm-based drug use. *Ann Rheum Dis.* **2015**, *74*, 89–95.

7. Hill, C.L.; Jones, G.; Lester, S.; Battersby, R.; Fedorova, T.; Hynes, K.; Proudman, S.; Cleland, L.G.; March, L. Effect Of Fish Oil On Structural Progression In Knee Osteoarthritis: A Two Year Randomized, Double-Blind Clinical Trial Comparing High Dose With Low Dose. *Arthritis* *Rheum.* **2013**, *65*, S914–S914.

8. Hill, C.L.; Jones, G.; March, L.; Battersby, R.; Hynes, K.; Fedorova, T.; Lester, S. Fish oil in knee osteoarthritis: A two year randomized, double-blind clinical trial comparing high dose with low dose. *Arthritis Rheum.* **2011**, *63*, 10.

S3.1.5. Uses Infusion (3 Trials, 3 Records)

1. Bahadori, B.; Uitz, E.; Thonhofer, R.; Trummer, M.; Pestemer-Lach, I.; McCarty, M.; Krejs, G.J. omega-3 Fatty acids infusions as adjuvant therapy in rheumatoid arthritis. *JPEN J. Parenter. Enter. Nutr.* **2010**, *34*, 151–155.

2. Uitz E, Bahadori B, Thonhofer R, et al. Omega-3 fatty acids as adjuvant therapy in rheumatoid arthritis. A double-blind, randomized, placebo-controlled trial. *Ann. Rheum. Dis.* **2006**, *65*, 347–347.

3. ISRCTN-Registration. N3 Fatty Acids as Adjuvant Therapy in Rheumatoid Arthritis (N3-Fettsäuren als Adjuvante Therapie bei Rheumatoider Arthritis. Available online: http://isrctn.com/ISRCTN48092875 (accessed on 12 May 2015).

S3.1.6. No Control Group (1 Trial, 1 Record)

1. Hosseini SA, Rahim F, Mola K. Omega-3 induced change in clinical parameters of rheumatoid arthritis. *Journal of Medical Sciences.* 2009, 9, 93–97.

S3.2. Possible Relevant Trials, No Obtainable Publications (2 Trials, 2 Records)

Obtained from the reference list from Geusens et al. (1994) (see below).

1. Tulleken, J.E.; Limburg, P.C.; Wassenaar, W.; Van Rijswijk, M.H. Nonsteroidal Anti-Inflammatory Drug Demand during Fish Oil Treatment in Rheumatoid Arthritis, Long Chain Omega-3 Polyunsaturated Fatty Acids in Rheumatoid Arthritis. Thesis, Rijksuniversiteit Groningen, Groningen, The Netherlands, 1991.

2. Tulleken, J.E.; Limburg, P.C.; Muskiet, F.A.J.; Kazemier, K.M.; Van Rijswijk, M.H. A Comparison between the Effects of Fish Oil Ethyl Esters and Fish Oil Triglycerides in Rheumatoid Arthritis, Long Chain Omega-3 Polyunsaturated Fatty Acids in Rheumatoid Arthritis. Thesis, Rijksuniversiteit Groningen, Groningen, The Netherlands, 1991.

3. Geusens, P.; Wouters, C.; Nijs, J.; Jiang, Y.; Dequeker, J. Long-term effect of omega-3 fatty acid supplementation in active rheumatoid arthritis. A 12-month, double-blind, controlled study. *Arthritis Rheum.* **1994**, *37*, 824–829.

Table S1 Search strategies for all databases. The search strategy was developed by N.K.S. and S.M.N. with assistance from S.T. It consisted of the three search concepts: (i) marine oil supplements; (ii) arthritis; and (iii) randomized trial design. Within each search concept various keywords and when available Medical Subject Heading (MeSH) terms were used, making the search sensitive rather than specific. Different terms for common marine oil supplemente (MOS) were found searching the Dietary Supplement Label Database (http://www.dsld.nlm.nih.gov/dsld/). The randomized controlled trial (RCT) search concept was modified from the *Cochrane Highly Sensitive Search Strategy for identifying randomized trials in MEDLINE: sensitivity-maximizing version (2008 revision); PubMed format* [1]. All the search strategies took their basis in the search strategy for PubMed, which was adjusted to fit with each of the other search strategies.

**Table S1.** Search Strategies for all Databases.

| **PubMed** | (essential fatty acids OR polyunsaturated OR PUFA OR PUFAs OR omega 3 OR *n-*3 fatty acids OR Docosahexaenoic Acid OR Eicosapentaenoic Acid OR ((fish OR marine OR krill OR haddock OR cod OR salmon OR mackerel OR herring OR anchovy OR sardine OR tuna OR skipjack OR halibut OR coalfish OR shark OR whale OR seal OR calamari OR algae OR algal OR spirulina OR seaweed OR euphausia superba OR haematococcus pluvialis OR hematococcus pluvialis OR lithothamnion corallioides OR nova scotia dulce OR ascophyllum nodosum OR chlorella OR lithothamnion calcareum OR gigartina OR mussel OR perna canaliculus) AND (oil OR oils OR fatty acids OR lipid OR lipids OR triglyceride OR triglycerides)))  AND  (musculoskeletal diseases OR polyarthritis OR polyarthritides OR arthritides OR arthriti* OR joint pain OR rheumatoid OR rheuma* OR osteoarthritis OR Chondrocalcinosis OR calcium pyrophosphate deposition disease OR Gout OR Periarthritis OR Sacroiliitis OR Spondylarthritis OR Spondylarthropathies OR Spondylarthropathy OR Spondyloarthritis OR Spondyloarthropathies OR Spondyloarthropathy OR ((Still’s OR caplan OR caplan’s OR Felty OR Felty’s OR Sjogren OR Sjogren’s OR gouty OR Wissler OR Wissler’s OR Wissler-Fanconi) AND (syndrome OR disease OR arthritis)))  AND  (randomized controlled trial [pt] OR controlled clinical trial [pt] OR placebo [tiab] OR drug therapy [sh] OR trial [tiab] OR groups [tiab] OR clinical trials as topic [mesh: noexp] OR Clinical Trial OR random* [tiab] OR random allocation [mh] OR single-blind method [mh] OR double-blind method [mh] OR cross-over studies)  NOT  (animals [mh] NOT humans [mh]) |
| --- | --- |
| **Web of Science *** | (essential fatty acids OR polyunsaturated OR PUFA* OR omega 3 OR *n-*3 fatty acids OR Docosahexaenoic OR Eicosapentaenoic OR ((fish OR marine OR krill OR haddock OR cod OR salmon OR mackerel OR herring OR anchovy OR sardine OR tuna OR skipjack OR halibut OR coalfish OR shark OR whale OR seal OR calamari OR algae OR algal OR spirulina OR seaweed OR euphausia superba OR haematococcus pluvialis OR hematococcus pluvialis OR lithothamnion corallioides OR nova scotia dulce OR ascophyllum nodosum OR chlorella OR lithothamnion calcareum OR gigartina OR mussel OR perna canaliculus) AND (oil* OR fatty acid* OR lipid* OR triglyceride *)))  AND  (musculoskeletal OR polyarthritis OR polyarthritides OR arthritides OR arthriti* OR joint pain OR rheuma* OR osteoarthritis OR Chondrocalcinosis OR calcium pyrophosphate deposition disease OR Gout OR Periarthritis OR Sacroiliitis OR Spondylarthritis OR Spondylarthropathies OR Spondylarthropathy OR Spondyloarthritis OR Spondyloarthropathies OR Spondyloarthropathy OR ((Still’s OR caplan * OR Felty* OR Sjogren* OR gouty OR Wissler *) AND (syndrome OR disease OR arthritis)))  AND  (controlled trial OR Clinical Trial OR placebo OR random * OR alloc * OR single-blind OR double-blind OR cross-over) |
| **CENTRAL ^†^** | **Box 1**  essential near fatty near acids OR polyunsaturated OR PUFA* OR omega 3 OR *n-*3 near fatty near acids OR Docosahexaenoic OR Eicosapentaenoic OR ((fish OR marine OR krill OR haddock OR cod OR salmon OR mackerel OR herring OR anchovy OR sardine OR tuna OR skipjack OR halibut OR coalfish OR shark OR whale OR seal OR calamari OR algae OR algal OR spirulina OR seaweed OR euphausia near superba OR haematococcus near pluvialis OR hematococcus near pluvialis OR lithothamnion near corallioides OR nova near scotia near dulce OR ascophyllum near nodosum OR chlorella OR lithothamnion near calcareum OR gigartina OR mussel OR perna near canaliculus) AND (oil OR fatty near acids OR lipids OR triglycerides))  **Box 2**  Musculoskeletal OR *arthritis OR *arthritides OR arthriti* OR joint near pain OR rheuma* OR Chondrocalcinosis OR calcium near pyrophosphate near deposition near disease OR Gout OR Periarthritis OR Sacroiliitis OR Spondylarth* OR Spondyloarth* OR ((stil* OR capla* OR Felt* OR Sjogre* OR gouty OR Wissle*) AND (syndrome OR disease OR arthritis)) |
| **EMBASE ^‡^** | #1: essential fatty acids OR polyunsaturated OR PUFA* OR omega 3 OR *n-*3 fatty acids OR Docosahexaenoic OR Eicosapentaenoic  #2: fish OR marine OR krill OR haddock OR cod OR salmon OR mackerel OR herring OR anchovy OR sardine OR tuna OR skipjack OR halibut OR coalfish OR shark OR whale OR seal OR calamari OR algae OR algal OR spirulina OR seaweed OR euphausia superba OR haematococcus pluvialis OR hematococcus pluvialis OR lithothamnion corallioides OR nova scotia dulce OR ascophyllum nodosum OR chlorella OR lithothamnion calcareum OR gigartina OR mussel OR perna canaliculus  #3: oil* OR fatty acid* OR lipid* OR triglyceride*  #4: musculoskeletal OR polyarthritis OR polyarthritides OR arthritides OR arthriti* OR joint pain OR rheuma* OR osteoarthritis OR Chondrocalcinosis OR calcium pyrophosphate deposition disease OR Gout OR Periarthritis OR Sacroiliitis OR Spondylarthritis OR Spondylarthropathies OR Spondylarthropathy OR Spondyloarthritis OR Spondyloarthropathies OR Spondyloarthropathy  #5: Still’s OR caplan* OR Felty* OR Sjogren* OR gouty OR Wissler*  #6: syndrome OR disease OR arthritis  #7: random*.tw. OR clinical trial* OR exp health care quality OR controlled trial OR placebo OR alloc* OR single-blind OR double-blind OR cross-over  #8: #2 AND #3  #9: #1 OR #8  #10: #5 AND #6  #11: #4 OR #10  #12: #9 AND #11 AND #7 |
| **Clinicaltrials.gov ^§^** | Condition  (A) musculoskeletal diseases OR polyarthritis OR polyarthritides OR arthritides OR arthritis OR joint pain OR rheumatoid OR rheumatic OR osteoarthritis OR Chondrocalcinosis OR calcium pyrophosphate deposition disease OR Gout OR Periarthritis  (B) Sacroiliitis OR Spondylarthritis OR Spondylarthropathies OR Spondylarthropathy OR Spondyloarthritis OR Spondyloarthropathies OR Spondyloarthropathy OR Still OR caplan OR Felty OR Sjogren OR gouty OR Wissler OR Wissler-Fanconi  Intervention  (1) essential fatty acids OR polyunsaturated OR PUFA OR PUFAs OR omega 3 OR *n-*3 fatty acids OR Docosahexaenoic OR Eicosapentaenoic OR fish OR marine OR oil OR oils OR fatty acids OR lipid OR lipids  (2) krill OR haddock OR cod OR salmon OR mackerel OR herring OR anchovy OR sardine OR tuna OR skipjack OR halibut OR coalfish OR shark OR whale OR seal OR calamari OR algae OR algal OR spirulina OR seaweed  (3) euphausia superba OR haematococcus pluvialis OR hematococcus pluvialis OR lithothamnion corallioides OR nova scotia dulce OR ascophyllum nodosum OR chlorella OR lithothamnion calcareum OR gigartina OR mussel OR perna canaliculus  Study type  Interventional Studies |
| **World Health Organization International Clinical Trial Registry Platform portal (ICTRP) ^\|\|^** | **Advanced Search**  Recruitment status  “ALL”  Condition  musculoskeletal OR polyarthritis OR arthriti* OR joint pain OR rheuma* OR osteoarthritis OR  Chondrocalcinosis OR Gout OR Periarthritis OR Sacroiliitis OR Spondylarthritis ORSpondyloarthritis OR Spondyloarthropathies OR Spondyloarthropathy OR Still’s disease OR Felty syndrome OR Sjogren syndrome  Intervention  essential fatty acids OR polyunsaturated OR PUFA OR PUFAs OR omega 3 OR *n-*3 fatty acids OR Docosahexaenoic Acid OR Eicosapentaenoic Acid OR oil OR oils OR fatty acids OR lipid OR lipids OR fish OR marine OR krill OR haddock OR cod OR salmon OR mackerel OR herring OR anchovy OR sardine OR tuna OR skipjack OR halibut OR coalfish OR shark OR whale OR seal OR calamari OR algae OR algal OR spirulina OR seaweed OR euphausia superba OR haematococcus pluvialis OR hematococcus pluvialis OR lithothamnion corallioides OR nova scotia dulce OR ascophyllum nodosum OR chlorella OR mussel OR perna canaliculus  **Basic Search**  Intervention AND condition 1  essential fatty acids AND musculoskeletal OR polyunsaturated AND musculoskeletal OR PUFA AND musculoskeletal OR PUFAs AND musculoskeletal OR omega 3 AND musculoskeletal OR *n-*3 fatty acids AND musculoskeletal OR Docosahexaenoic Acid AND musculoskeletal OR Eicosapentaenoic Acid AND musculoskeletal OR oil AND musculoskeletal OR oils AND musculoskeletal OR fatty acids AND musculoskeletal OR lipid AND musculoskeletal OR lipids AND musculoskeletal OR fish AND musculoskeletal OR marine AND musculoskeletal OR krill AND musculoskeletal OR haddock AND musculoskeletal OR cod AND musculoskeletal OR salmon AND musculoskeletal OR mackerel AND musculoskeletal OR herring AND musculoskeletal OR anchovy AND musculoskeletal OR sardine AND musculoskeletal OR tuna AND musculoskeletal OR skipjack AND musculoskeletal OR halibut AND musculoskeletal OR coalfish AND musculoskeletal OR shark AND musculoskeletal OR whale AND musculoskeletal OR seal AND musculoskeletal OR calamari AND musculoskeletal OR algae AND musculoskeletal OR algal AND musculoskeletal OR spirulina AND musculoskeletal OR seaweed AND musculoskeletal OR euphausia superba AND musculoskeletal OR haematococcus pluvialis AND musculoskeletal OR hematococcus pluvialis AND musculoskeletal OR lithothamnion corallioides AND musculoskeletal OR nova scotia dulce AND musculoskeletal OR ascophyllum nodosum AND musculoskeletal OR chlorella AND musculoskeletal OR mussel AND musculoskeletal OR perna canaliculus AND musculoskeletal  Intervention AND condition 2  essential fatty acids AND polyarthritis OR polyunsaturated AND polyarthritis OR PUFA AND polyarthritis OR PUFAs AND polyarthritis OR omega 3 AND polyarthritis OR *n-*3 fatty acids AND polyarthritis OR Docosahexaenoic Acid AND polyarthritis OR Eicosapentaenoic Acid AND polyarthritis OR oil AND polyarthritis OR oils AND polyarthritis OR fatty acids AND polyarthritis OR lipid AND polyarthritis OR lipids AND polyarthritis OR fish AND polyarthritis OR marine AND polyarthritis OR krill AND polyarthritis OR haddock AND polyarthritis OR cod AND polyarthritis OR salmon AND polyarthritis OR mackerel AND polyarthritis OR herring AND polyarthritis OR anchovy AND polyarthritis OR sardine AND polyarthritis OR tuna AND polyarthritis OR skipjack AND polyarthritis OR halibut AND polyarthritis OR coalfish AND polyarthritis OR shark AND polyarthritis OR whale AND polyarthritis OR seal AND polyarthritis OR calamari AND polyarthritis OR algae AND polyarthritis OR algal AND polyarthritis OR spirulina AND polyarthritis OR seaweed AND polyarthritis OR euphausia superba AND polyarthritis OR haematococcus pluvialis AND polyarthritis OR hematococcus pluvialis AND polyarthritis OR lithothamnion corallioides AND polyarthritis OR nova scotia dulce AND polyarthritis OR ascophyllum nodosum AND polyarthritis OR chlorella AND polyarthritis OR mussel AND polyarthritis OR perna canaliculus AND polyarthritis  Intervention AND condition 3  essential fatty acids AND arthriti* OR polyunsaturated AND arthriti* OR PUFA AND arthriti* OR PUFAs AND arthriti* OR omega 3 AND arthriti* OR *n-*3 fatty acids AND arthriti* OR Docosahexaenoic Acid AND arthriti* OR Eicosapentaenoic Acid AND arthriti* OR oil AND arthriti* OR oils AND arthriti* OR fatty acids AND arthriti* OR lipid AND arthriti* OR lipids AND arthriti* OR fish AND arthriti* OR marine AND arthriti* OR krill AND arthriti* OR haddock AND arthriti* OR cod AND arthriti* OR salmon AND arthriti* OR mackerel AND arthriti* OR herring AND arthriti* OR anchovy AND arthriti* OR sardine AND arthriti* OR tuna AND arthriti* OR skipjack AND arthriti* OR halibut AND arthriti* OR coalfish AND arthriti* OR shark AND arthriti* OR whale AND arthriti* OR seal AND arthriti* OR calamari AND arthriti* OR algae AND arthriti* OR algal AND arthriti* OR spirulina AND arthriti* OR seaweed AND arthriti* OR euphausia superba AND arthriti* OR haematococcus pluvialis AND arthriti* OR hematococcus pluvialis AND arthriti* OR lithothamnion corallioides AND arthriti* OR nova scotia dulce AND arthriti* OR ascophyllum nodosum AND arthriti* OR chlorella AND arthriti* OR mussel AND arthriti* OR perna canaliculus AND arthriti*  Intervention AND condition 4  essential fatty acids AND joint pain OR polyunsaturated AND joint pain OR PUFA AND joint pain OR PUFAs AND joint pain OR omega 3 AND joint pain OR *n-*3 fatty acids AND joint pain OR Docosahexaenoic Acid AND joint pain OR Eicosapentaenoic Acid AND joint pain OR oil AND joint pain OR oils AND joint pain OR fatty acids AND joint pain OR lipid AND joint pain OR lipids AND joint pain OR fish AND joint pain OR marine AND joint pain OR krill AND joint pain OR haddock AND joint pain OR cod AND joint pain OR salmon AND joint pain OR mackerel AND joint pain OR herring AND joint pain OR anchovy AND joint pain OR sardine AND joint pain OR tuna AND joint pain OR skipjack AND joint pain OR halibut AND joint pain OR coalfish AND joint pain OR shark AND joint pain OR whale AND joint pain OR seal AND joint pain OR calamari AND joint pain OR algae AND joint pain OR algal AND joint pain OR spirulina AND joint pain OR seaweed AND joint pain OR euphausia superba AND joint pain OR haematococcus pluvialis AND joint pain OR hematococcus pluvialis AND joint pain OR lithothamnion corallioides AND joint pain OR nova scotia dulce AND joint pain OR ascophyllum nodosum AND joint pain OR chlorella AND joint pain OR mussel AND joint pain OR perna canaliculus AND joint pain  Intervention AND condition 5  essential fatty acids AND rheuma* OR polyunsaturated AND rheuma* OR PUFA AND rheuma* OR PUFAs AND rheuma* OR omega 3 AND rheuma* OR *n-*3 fatty acids AND rheuma* OR Docosahexaenoic Acid AND rheuma* OR Eicosapentaenoic Acid AND rheuma* OR oil AND rheuma* OR oils AND rheuma* OR fatty acids AND rheuma* OR lipid AND rheuma* OR lipids AND rheuma* OR fish AND rheuma* OR marine AND rheuma* OR krill AND rheuma* OR haddock AND rheuma* OR cod AND rheuma* OR salmon AND rheuma* OR mackerel AND rheuma* OR herring AND rheuma* OR anchovy AND rheuma* OR sardine AND rheuma* OR tuna AND rheuma* OR skipjack AND rheuma* OR halibut AND rheuma* OR coalfish AND rheuma* OR shark AND rheuma* OR whale AND rheuma* OR seal AND rheuma* OR calamari AND rheuma* OR algae AND rheuma* OR algal AND rheuma* OR spirulina AND rheuma* OR seaweed AND rheuma* OR euphausia superba AND rheuma* OR haematococcus pluvialis AND rheuma* OR hematococcus pluvialis AND rheuma* OR lithothamnion corallioides AND rheuma* OR nova scotia dulce AND rheuma* OR ascophyllum nodosum AND rheuma* OR chlorella AND rheuma* OR mussel AND rheuma* OR perna canaliculus AND rheuma*  Intervention AND condition 6  essential fatty acids AND osteoarthritis OR polyunsaturated AND osteoarthritis OR PUFA AND osteoarthritis OR PUFAs AND osteoarthritis OR omega 3 AND osteoarthritis OR *n-*3 fatty acids AND osteoarthritis OR Docosahexaenoic Acid AND osteoarthritis OR Eicosapentaenoic Acid AND osteoarthritis OR oil AND osteoarthritis OR oils AND osteoarthritis OR fatty acids AND osteoarthritis OR lipid AND osteoarthritis OR lipids AND osteoarthritis OR fish AND osteoarthritis OR marine AND osteoarthritis OR krill AND osteoarthritis OR haddock AND osteoarthritis OR cod AND osteoarthritis OR salmon AND osteoarthritis OR mackerel AND osteoarthritis OR herring AND osteoarthritis OR anchovy AND osteoarthritis OR sardine AND osteoarthritis OR tuna AND osteoarthritis OR skipjack AND osteoarthritis OR halibut AND osteoarthritis OR coalfish AND osteoarthritis OR shark AND osteoarthritis OR whale AND osteoarthritis OR seal AND osteoarthritis OR calamari AND osteoarthritis OR algae AND osteoarthritis OR algal AND osteoarthritis OR spirulina AND osteoarthritis OR seaweed AND osteoarthritis OR euphausia superba AND osteoarthritis OR haematococcus pluvialis AND osteoarthritis OR hematococcus pluvialis AND osteoarthritis OR lithothamnion corallioides AND osteoarthritis OR nova scotia dulce AND osteoarthritis OR ascophyllum nodosum AND osteoarthritis OR chlorella AND osteoarthritis OR mussel AND osteoarthritis OR perna canaliculus AND osteoarthritis  Intervention AND condition 7  essential fatty acids AND Still’s disease OR polyunsaturated AND Still’s disease OR PUFA AND Still’s disease OR PUFAs AND Still’s disease OR omega 3 AND Still’s disease OR *n-*3 fatty acids AND Still’s disease OR Docosahexaenoic Acid AND Still’s disease OR Eicosapentaenoic Acid AND Still’s disease OR oil AND Still’s disease OR oils AND Still’s disease OR fatty acids AND Still’s disease OR lipid AND Still’s disease OR lipids AND Still’s disease OR fish AND Still’s disease OR marine AND Still’s disease OR krill AND Still’s disease OR haddock AND Still’s disease OR cod AND Still’s disease OR salmon AND Still’s disease OR mackerel AND Still’s disease OR herring AND Still’s disease OR anchovy AND Still’s disease OR sardine AND Still’s disease OR tuna AND Still’s disease OR skipjack AND Still’s disease OR halibut AND Still’s disease OR coalfish AND Still’s disease OR shark AND Still’s disease OR whale AND Still’s disease OR seal AND Still’s disease OR calamari AND Still’s disease OR algae AND Still’s disease OR algal AND Still’s disease OR spirulina AND Still’s disease OR seaweed AND Still’s disease OR euphausia superba AND Still’s disease OR haematococcus pluvialis AND Still’s disease OR hematococcus pluvialis AND Still’s disease OR lithothamnion corallioides AND Still’s disease OR nova scotia dulce AND Still’s disease OR ascophyllum nodosum AND Still’s disease OR chlorella AND Still’s disease OR mussel AND Still’s disease OR perna canaliculus AND Still’s disease  Intervention AND condition 8 (a)  essential fatty acids AND Chondrocalcinosis OR polyunsaturated AND Chondrocalcinosis OR PUFA AND Chondrocalcinosis OR PUFAs AND Chondrocalcinosis OR omega 3 AND Chondrocalcinosis OR *n-*3 fatty acids AND Chondrocalcinosis OR Docosahexaenoic Acid AND Chondrocalcinosis OR Eicosapentaenoic Acid AND Chondrocalcinosis OR oil AND Chondrocalcinosis OR oils AND Chondrocalcinosis OR fatty acids AND Chondrocalcinosis OR lipid AND Chondrocalcinosis OR lipids AND Chondrocalcinosis OR fish AND Chondrocalcinosis OR marine AND Chondrocalcinosis OR krill AND Chondrocalcinosis OR haddock AND Chondrocalcinosis OR cod AND Chondrocalcinosis OR salmon AND Chondrocalcinosis OR mackerel AND Chondrocalcinosis OR herring AND Chondrocalcinosis OR anchovy AND Chondrocalcinosis OR sardine AND Chondrocalcinosis OR tuna AND Chondrocalcinosis OR skipjack AND Chondrocalcinosis OR halibut AND Chondrocalcinosis OR coalfish AND Chondrocalcinosis OR shark AND Chondrocalcinosis OR whale AND Chondrocalcinosis OR seal AND Chondrocalcinosis OR calamari AND Chondrocalcinosis OR algae AND Chondrocalcinosis OR algal AND Chondrocalcinosis OR spirulina AND Chondrocalcinosis OR seaweed AND Chondrocalcinosis OR euphausia superba AND Chondrocalcinosis OR haematococcus pluvialis AND Chondrocalcinosis OR hematococcus pluvialis AND Chondrocalcinosis OR lithothamnion corallioides AND Chondrocalcinosis OR nova scotia dulce AND Chondrocalcinosis OR ascophyllum nodosum AND Chondrocalcinosis OR chlorella AND Chondrocalcinosis  Intervention AND condition 8 (b)  mussel AND Chondrocalcinosis OR perna canaliculus AND Chondrocalcinosis  Intervention AND condition 9  essential fatty acids AND Gout OR polyunsaturated AND Gout OR PUFA AND Gout OR PUFAs AND Gout OR omega 3 AND Gout OR *n-*3 fatty acids AND Gout OR Docosahexaenoic Acid AND Gout OR Eicosapentaenoic Acid AND Gout OR oil AND Gout OR oils AND Gout OR fatty acids AND Gout OR lipid AND Gout OR lipids AND Gout OR fish AND Gout OR marine AND Gout OR krill AND Gout OR haddock AND Gout OR cod AND Gout OR salmon AND Gout OR mackerel AND Gout OR herring AND Gout OR anchovy AND Gout OR sardine AND Gout OR tuna AND Gout OR skipjack AND Gout OR halibut AND Gout OR coalfish AND Gout OR shark AND Gout OR whale AND Gout OR seal AND Gout OR calamari AND Gout OR algae AND Gout OR algal AND Gout OR spirulina AND Gout OR seaweed AND Gout OR euphausia superba AND Gout OR haematococcus pluvialis AND Gout OR hematococcus pluvialis AND Gout OR lithothamnion corallioides AND Gout OR nova scotia dulce AND Gout OR ascophyllum nodosum AND Gout OR chlorella AND Gout OR mussel AND Gout OR perna canaliculus AND Gout  Intervention AND condition 10  essential fatty acids AND Periarthritis OR polyunsaturated AND Periarthritis OR PUFA AND Periarthritis OR PUFAs AND Periarthritis OR omega 3 AND Periarthritis OR *n*-3 fatty acids AND Periarthritis OR Docosahexaenoic Acid AND Periarthritis OR Eicosapentaenoic Acid AND Periarthritis OR oil AND Periarthritis OR oils AND Periarthritis OR fatty acids AND Periarthritis OR lipid AND Periarthritis OR lipids AND Periarthritis OR fish AND Periarthritis OR marine AND Periarthritis OR krill AND Periarthritis OR haddock AND Periarthritis OR cod AND Periarthritis OR salmon AND Periarthritis OR mackerel AND Periarthritis OR herring AND Periarthritis OR anchovy AND Periarthritis OR sardine AND Periarthritis OR tuna AND Periarthritis OR skipjack AND Periarthritis OR halibut AND Periarthritis OR coalfish AND Periarthritis OR shark AND Periarthritis OR whale AND Periarthritis OR seal AND Periarthritis OR calamari AND Periarthritis OR algae AND Periarthritis OR algal AND Periarthritis OR spirulina AND Periarthritis OR seaweed AND Periarthritis OR euphausia superba AND Periarthritis OR haematococcus pluvialis AND Periarthritis OR hematococcus pluvialis AND Periarthritis OR lithothamnion corallioides AND Periarthritis OR nova scotia dulce AND Periarthritis OR ascophyllum nodosum AND Periarthritis OR chlorella AND Periarthritis OR mussel AND Periarthritis OR perna canaliculus AND Periarthritis  Intervention AND condition 11  essential fatty acids AND Sacroiliitis OR polyunsaturated AND Sacroiliitis OR PUFA AND Sacroiliitis OR PUFAs AND Sacroiliitis OR omega 3 AND Sacroiliitis OR *n*-3 fatty acids AND Sacroiliitis OR Docosahexaenoic Acid AND Sacroiliitis OR Eicosapentaenoic Acid AND Sacroiliitis OR oil AND Sacroiliitis OR oils AND Sacroiliitis OR fatty acids AND Sacroiliitis OR lipid AND Sacroiliitis OR lipids AND Sacroiliitis OR fish AND Sacroiliitis OR marine AND Sacroiliitis OR krill AND Sacroiliitis OR haddock AND Sacroiliitis OR cod AND Sacroiliitis OR salmon AND Sacroiliitis OR mackerel AND Sacroiliitis OR herring AND Sacroiliitis OR anchovy AND Sacroiliitis OR sardine AND Sacroiliitis OR tuna AND Sacroiliitis OR skipjack AND Sacroiliitis OR halibut AND Sacroiliitis OR coalfish AND Sacroiliitis OR shark AND Sacroiliitis OR whale AND Sacroiliitis OR seal AND Sacroiliitis OR calamari AND Sacroiliitis OR algae AND Sacroiliitis OR algal AND Sacroiliitis OR spirulina AND Sacroiliitis OR seaweed AND Sacroiliitis OR euphausia superba AND Sacroiliitis OR haematococcus pluvialis AND Sacroiliitis OR hematococcus pluvialis AND Sacroiliitis OR lithothamnion corallioides AND Sacroiliitis OR nova scotia dulce AND Sacroiliitis OR ascophyllum nodosum AND Sacroiliitis OR chlorella AND Sacroiliitis OR mussel AND Sacroiliitis OR perna canaliculus AND Sacroiliitis  Intervention AND condition 12 (a)  essential fatty acids AND Spondylarthritis OR polyunsaturated AND Spondylarthritis OR PUFA AND Spondylarthritis OR PUFAs AND Spondylarthritis OR omega 3 AND Spondylarthritis OR *n*-3 fatty acids AND Spondylarthritis OR Docosahexaenoic Acid AND Spondylarthritis OR Eicosapentaenoic Acid AND Spondylarthritis OR oil AND Spondylarthritis OR oils AND Spondylarthritis OR fatty acids AND Spondylarthritis OR lipid AND Spondylarthritis OR lipids AND Spondylarthritis OR fish AND Spondylarthritis OR marine AND Spondylarthritis OR krill AND Spondylarthritis OR haddock AND Spondylarthritis OR cod AND Spondylarthritis OR salmon AND Spondylarthritis OR mackerel AND Spondylarthritis OR herring AND Spondylarthritis OR anchovy AND Spondylarthritis OR sardine AND Spondylarthritis OR tuna AND Spondylarthritis OR skipjack AND Spondylarthritis OR halibut AND Spondylarthritis OR coalfish AND Spondylarthritis OR shark AND Spondylarthritis OR whale AND Spondylarthritis OR seal AND Spondylarthritis OR calamari AND Spondylarthritis OR algae AND Spondylarthritis OR algal AND Spondylarthritis OR spirulina AND Spondylarthritis OR seaweed AND Spondylarthritis OR euphausia superba AND Spondylarthritis OR haematococcus pluvialis AND Spondylarthritis OR hematococcus pluvialis AND Spondylarthritis OR lithothamnion corallioides AND Spondylarthritis OR nova scotia dulce AND Spondylarthritis OR ascophyllum nodosum AND Spondylarthritis OR chlorella AND Spondylarthritis OR mussel AND Spondylarthritis  Intervention AND condition 12 (b)  perna canaliculus AND Spondylarthritis  Intervention AND condition 13 (a)  essential fatty acids AND Spondyloarthritis OR polyunsaturated AND Spondyloarthritis OR PUFA AND Spondyloarthritis OR PUFAs AND Spondyloarthritis OR omega 3 AND Spondyloarthritis OR *n*-3 fatty acids AND Spondyloarthritis OR Docosahexaenoic Acid AND Spondyloarthritis OR Eicosapentaenoic Acid AND Spondyloarthritis OR oil AND Spondyloarthritis OR oils AND Spondyloarthritis OR fatty acids AND Spondyloarthritis OR lipid AND Spondyloarthritis OR lipids AND Spondyloarthritis OR fish AND Spondyloarthritis OR marine AND Spondyloarthritis OR krill AND Spondyloarthritis OR haddock AND Spondyloarthritis OR cod AND Spondyloarthritis OR salmon AND Spondyloarthritis OR mackerel AND Spondyloarthritis OR herring AND Spondyloarthritis OR anchovy AND Spondyloarthritis OR sardine AND Spondyloarthritis OR tuna AND Spondyloarthritis OR skipjack AND Spondyloarthritis OR halibut AND Spondyloarthritis OR coalfish AND Spondyloarthritis OR shark AND Spondyloarthritis OR whale AND Spondyloarthritis OR seal AND Spondyloarthritis OR calamari AND Spondyloarthritis OR algae AND Spondyloarthritis OR algal AND Spondyloarthritis OR spirulina AND Spondyloarthritis OR seaweed AND Spondyloarthritis OR euphausia superba AND Spondyloarthritis OR haematococcus pluvialis AND Spondyloarthritis OR hematococcus pluvialis AND Spondyloarthritis OR lithothamnion corallioides AND Spondyloarthritis OR nova scotia dulce AND Spondyloarthritis OR ascophyllum nodosum AND Spondyloarthritis OR chlorella AND Spondyloarthritis  Intervention AND condition 13 (b)  mussel AND Spondyloarthritis OR perna canaliculus AND Spondyloarthritis  Intervention AND condition 14 (a)  essential fatty acids AND Spondyloarthropathies OR polyunsaturated AND Spondyloarthropathies OR PUFA AND Spondyloarthropathies OR PUFAs AND Spondyloarthropathies OR omega 3 AND Spondyloarthropathies OR *n*-3 fatty acids AND Spondyloarthropathies OR Docosahexaenoic Acid AND Spondyloarthropathies OR Eicosapentaenoic Acid AND Spondyloarthropathies OR oil AND Spondyloarthropathies OR oils AND Spondyloarthropathies OR fatty acids AND Spondyloarthropathies OR lipid AND Spondyloarthropathies OR lipids AND Spondyloarthropathies OR fish AND Spondyloarthropathies OR marine AND Spondyloarthropathies OR krill AND Spondyloarthropathies OR haddock AND Spondyloarthropathies OR cod AND Spondyloarthropathies OR salmon AND Spondyloarthropathies OR mackerel AND Spondyloarthropathies OR herring AND Spondyloarthropathies OR anchovy AND Spondyloarthropathies OR sardine AND Spondyloarthropathies OR tuna AND Spondyloarthropathies OR skipjack AND Spondyloarthropathies OR halibut AND Spondyloarthropathies OR coalfish AND Spondyloarthropathies OR shark AND Spondyloarthropathies OR whale AND Spondyloarthropathies OR seal AND Spondyloarthropathies OR calamari AND Spondyloarthropathies OR algae AND Spondyloarthropathies OR algal AND Spondyloarthropathies OR spirulina AND Spondyloarthropathies OR seaweed AND Spondyloarthropathies OR euphausia superba AND Spondyloarthropathies OR haematococcus pluvialis AND Spondyloarthropathies OR hematococcus pluvialis AND Spondyloarthropathies  Intervention AND condition 14 (b)  lithothamnion corallioides AND Spondyloarthropathies OR nova scotia dulce AND Spondyloarthropathies OR ascophyllum nodosum AND Spondyloarthropathies OR chlorella AND Spondyloarthropathies OR mussel AND Spondyloarthropathies OR perna canaliculus AND Spondyloarthropathies  Intervention AND condition 15 (a)  essential fatty acids AND Spondyloarthropathy OR polyunsaturated AND Spondyloarthropathy OR PUFA AND Spondyloarthropathy OR PUFAs AND Spondyloarthropathy OR omega 3 AND Spondyloarthropathy OR *n*-3 fatty acids AND Spondyloarthropathy OR Docosahexaenoic Acid AND Spondyloarthropathy OR Eicosapentaenoic Acid AND Spondyloarthropathy OR oil AND Spondyloarthropathy OR oils AND Spondyloarthropathy OR fatty acids AND Spondyloarthropathy OR lipid AND Spondyloarthropathy OR lipids AND Spondyloarthropathy OR fish AND Spondyloarthropathy OR marine AND Spondyloarthropathy OR krill AND Spondyloarthropathy OR haddock AND Spondyloarthropathy OR cod AND Spondyloarthropathy OR salmon AND Spondyloarthropathy OR mackerel AND Spondyloarthropathy OR herring AND Spondyloarthropathy OR anchovy AND Spondyloarthropathy OR sardine AND Spondyloarthropathy OR tuna AND Spondyloarthropathy OR skipjack AND Spondyloarthropathy OR halibut AND Spondyloarthropathy OR coalfish AND Spondyloarthropathy OR shark AND Spondyloarthropathy OR whale AND Spondyloarthropathy OR seal AND Spondyloarthropathy OR calamari AND Spondyloarthropathy OR algae AND Spondyloarthropathy OR algal AND Spondyloarthropathy OR spirulina AND Spondyloarthropathy OR seaweed AND Spondyloarthropathy OR euphausia superba AND Spondyloarthropathy OR haematococcus pluvialis AND Spondyloarthropathy OR hematococcus pluvialis AND Spondyloarthropathy OR lithothamnion corallioides AND Spondyloarthropathy OR nova scotia dulce AND Spondyloarthropathy  Intervention AND condition 15 (b)  ascophyllum nodosum AND Spondyloarthropathy OR chlorella AND Spondyloarthropathy OR mussel AND Spondyloarthropathy OR perna canaliculus AND Spondyloarthropathy  Intervention AND condition 16  essential fatty acids AND Felty syndrome OR polyunsaturated AND Felty syndrome OR PUFA AND Felty syndrome OR PUFAs AND Felty syndrome OR omega 3 AND Felty syndrome OR *n*-3 fatty acids AND Felty syndrome OR Docosahexaenoic Acid AND Felty syndrome OR Eicosapentaenoic Acid AND Felty syndrome OR oil AND Felty syndrome OR oils AND Felty syndrome OR fatty acids AND Felty syndrome OR lipid AND Felty syndrome OR lipids AND Felty syndrome OR fish AND Felty syndrome OR marine AND Felty syndrome OR krill AND Felty syndrome OR haddock AND Felty syndrome OR cod AND Felty syndrome OR salmon AND Felty syndrome OR mackerel AND Felty syndrome OR herring AND Felty syndrome OR anchovy AND Felty syndrome OR sardine AND Felty syndrome OR tuna AND Felty syndrome OR skipjack AND Felty syndrome OR halibut AND Felty syndrome OR coalfish AND Felty syndrome OR shark AND Felty syndrome OR whale AND Felty syndrome OR seal AND Felty syndrome OR calamari AND Felty syndrome OR algae AND Felty syndrome OR algal AND Felty syndrome OR spirulina AND Felty syndrome OR seaweed AND Felty syndrome OR euphausia superba AND Felty syndrome OR haematococcus pluvialis AND Felty syndrome OR hematococcus pluvialis AND Felty syndrome OR lithothamnion corallioides AND Felty syndrome OR nova scotia dulce AND Felty syndrome OR ascophyllum nodosum AND Felty syndrome OR chlorella AND Felty syndrome OR mussel AND Felty syndrome OR perna canaliculus AND Felty syndrome  Intervention AND condition 17 (a)  essential fatty acids AND Sjogren syndrome OR polyunsaturated AND Sjogren syndrome OR PUFA AND Sjogren syndrome OR PUFAs AND Sjogren syndrome OR omega 3 AND Sjogren syndrome OR *n*-3 fatty acids AND Sjogren syndrome OR Docosahexaenoic Acid AND Sjogren syndrome OR Eicosapentaenoic Acid AND Sjogren syndrome OR oil AND Sjogren syndrome OR oils AND Sjogren syndrome OR fatty acids AND Sjogren syndrome OR lipid AND Sjogren syndrome OR lipids AND Sjogren syndrome OR fish AND Sjogren syndrome OR marine AND Sjogren syndrome OR krill AND Sjogren syndrome OR haddock AND Sjogren syndrome OR cod AND Sjogren syndrome OR salmon AND Sjogren syndrome OR mackerel AND Sjogren syndrome OR herring AND Sjogren syndrome OR anchovy AND Sjogren syndrome OR sardine AND Sjogren syndrome OR tuna AND Sjogren syndrome OR skipjack AND Sjogren syndrome OR halibut AND Sjogren syndrome OR coalfish AND Sjogren syndrome OR shark AND Sjogren syndrome OR whale AND Sjogren syndrome OR seal AND Sjogren syndrome OR calamari AND Sjogren syndrome OR algae AND Sjogren syndrome OR algal AND Sjogren syndrome OR spirulina AND Sjogren syndrome OR seaweed AND Sjogren syndrome OR euphausia superba AND Sjogren syndrome OR haematococcus pluvialis AND Sjogren syndrome OR hematococcus pluvialis AND Sjogren syndrome OR lithothamnion corallioides AND Sjogren syndrome OR nova scotia dulce AND Sjogren syndrome OR ascophyllum nodosum AND Sjogren syndrome OR chlorella AND Sjogren syndrome  Intervention AND condition 17 (b)  mussel AND Sjogren syndrome OR perna canaliculus AND Sjogren syndrome |

* The search was applied for “Topic”; ^†^ Two boxes were to be used simultaneously, combined with “AND”, and both set at “Search All Text”. The “Search Limit” “Trials” was not used, in order to obtain relevant reviews from which references could be searched, hence the entire Cochrane Library was searched; ^‡^ The search was carried out in “Advanced Search” and `^#^´ indicates separate searches conducted and combined. The last line, #12, is the final search. The RCT filter with the highest sensitivity developed by Wong, et al. [2] for use in EMBASE was applied when searching this database; ^§^ The search was conducted within “Advanced search” only, since this interface improves precision without loss of sensitivity compared with the “Basic search” [3]. Because of limited space in the search boxes, six searches were conducted so that all possible combinations were applied. E.g., search one: condition (A) combined with intervention (1) combined with study type “Interventional Studies”; search two: condition (A) combined with intervention (2) combined with study type “Interventional Studies”, and so forth; ^||^ The search was conducted in the basic and advanced interface, since the advanced interface may reduce sensitivity compared with the basic interface [3]. Due to limited space, the condition-part was shortened down, making sure it did not result in less hits, and parentheses were removed, which cannot be used. The basic search was carried out 17 times, one for each condition-term, since a search “term a” OR “term b” AND “term c” will be searched as “term a” OR (“term b” AND “term c”).

References

1. Higgins, J.P.; Green, S. Box 6.4.a: Cochrane Highly Sensitive Search Strategy for Identifying Randomized Trials in MEDLINE: Sensitivity-Maximizing Version (2008 Revision); PubMed Format. Available online: http://handbook.cochrane.org/chapter_6/box_6_4_a_cochrane_hsss_2008_sensmax_pubmed.htm (accessed on 2 March 2015).
2. Wong, S.S.L.; Wilczynski, N.L.; Haynes, R.B.; Hedges, T. Developing optimal search strategies for detecting clinically sound treatment studies in EMBASE. *J. Med. Libr. Assoc.* **2006**, *94*, 41–47.
3. Glanville, J.M.; Duffy, S.; McCool, R.; Varley, D. Searching ClinicalTrials.gov and the International Clinical Trials Registry Platform to inform systematic reviews: what are the optimal search approaches? *J. Med. Libr. Assoc.* **2014**, *102*, 177–183.

**Table S2.** Outcome matrix. Summary of outcomes, for which results have been reported, for studies included in the meta-analysis.

| Reference | **Pain** | | | | | | | | | | | | | | **Function** | | | | | | | | | | | | | | | | | | | | | **Inflammation** | | | | | | | | | | | | | | | |
| --- | --- | --- | --- | --- | --- | --- | --- | --- | --- | --- | --- | --- | --- | --- | --- | --- | --- | --- | --- | --- | --- | --- | --- | --- | --- | --- | --- | --- | --- | --- | --- | --- | --- | --- | --- | --- | --- | --- | --- | --- | --- | --- | --- | --- | --- | --- | --- | --- | --- | --- | --- |
|  | Pain scale including VAS | WOMAC | Tender/painful joint count | Joint tenderness/pain index | Patient’s assessment of pain | Physician ‘s assessment of pain | Ritchie’s articular index | Pain throughout 24 hours | NSAID requirement | WOMAC, pain | Pain evaluation hip | Pain evaluation knee | Bodily pain (SF-36 part) | Global assessment of pain by patient | Interval to onset of fatigue, hours | Grip strength | MHAQ | HAQ | Patient’s assessment of disability | Patient assessed disability | Time taken to walk 18 metres | Time to walk a specific distance | Interference with acticities, VAS | WOMAC for function | PM fatique | Utilities index | Physical functioning (SF-36 part) | Onset of fatique after walking | Classification of funtional status in RA * | CAIMS-2SF, physical domain | Daily activity score | Fries index | Dynamic balance | Stair climb | Sit-to-stand | CRP | TNF-alpha | ESR | Calprotectin (feces)** | IL-1 beta | IL-2 | IL-6 | IL-10 | PGE2 | C3d | C3 | C4 | 11-TXB2 | PG-M | LTB4 | LTB5 |
| Belch 1986 |  |  |  |  |  |  |  |  | o |  |  |  |  |  |  |  |  |  |  |  |  |  |  |  |  |  |  |  |  |  |  |  |  |  |  | o |  | o |  |  |  |  |  |  |  |  |  |  |  |  |  |
| Belch 1988 | v |  |  |  |  |  | v |  | v |  |  |  |  |  |  | v |  |  |  |  |  |  |  |  |  |  |  |  |  |  |  |  |  |  |  | v |  | v |  |  |  |  |  |  |  |  |  |  |  |  |  |
| Cleland 1988 | v |  | v |  |  |  |  |  |  |  |  |  |  |  |  | v |  |  |  |  |  | v |  |  |  |  |  |  |  |  |  |  |  |  |  |  |  | o |  |  |  |  |  |  |  |  |  |  |  | v | v |
| Stammers 1989 | v |  |  |  |  |  |  |  |  |  |  |  |  |  |  |  |  |  |  |  |  |  | v |  |  |  |  |  |  |  |  |  |  |  |  |  |  |  |  |  |  |  |  |  |  |  |  |  |  |  |  |
| Kremer 1990 |  |  | v |  | v | v |  |  |  |  |  |  |  |  | v | v |  |  |  |  |  |  |  |  |  |  |  |  |  |  |  |  |  |  |  |  |  | o |  | v | v |  |  |  |  |  |  |  |  | v |  |
| Tulleken 1990 | v |  |  | v |  |  | v |  |  |  |  |  |  |  |  | v |  |  |  |  |  |  |  |  |  |  |  |  |  |  |  |  |  |  |  | v |  | v |  |  |  |  |  |  |  |  |  |  |  |  |  |
| van der Tempel 1990 | v |  |  |  | v |  |  |  |  |  |  |  |  |  |  | o |  |  |  |  |  |  |  |  |  |  |  |  |  |  |  |  |  |  |  | v |  | v |  |  |  |  |  |  |  |  |  |  |  | v | v |
| Espersen 1992 |  |  |  |  |  |  | v |  |  |  |  |  |  |  |  |  |  |  |  |  |  |  |  |  |  |  |  |  |  |  |  |  |  |  |  |  | v |  |  | v |  |  |  |  | v |  |  |  |  |  |  |
| Kjeldsen-Kragh 1992 | v |  | v |  |  |  | v |  |  |  |  |  |  |  |  | v |  | v |  |  |  |  |  |  |  |  |  |  |  |  |  |  |  |  |  | v |  | v |  |  |  |  |  |  |  |  |  |  |  |  |  |
| Magaró 1992 | v |  |  |  |  |  | v |  |  |  |  |  |  |  |  | v |  |  |  |  |  |  |  |  |  |  |  |  |  |  |  |  |  |  |  |  |  | v |  |  |  |  |  |  |  |  |  |  |  |  |  |
| Nielsen 1992^b^ | v |  |  | v |  |  |  |  |  |  |  |  |  |  |  | v |  |  |  |  |  |  |  |  |  |  |  |  |  |  | v |  |  |  |  | v |  | v |  |  |  |  |  |  |  |  |  |  |  |  |  |
| (Faarvang 1994)^b^ | v |  |  | v |  |  |  |  | o |  |  |  |  |  |  | v |  |  |  |  |  |  |  |  |  |  |  |  |  |  | v |  |  |  |  | v |  | v |  |  |  |  |  |  |  |  |  |  |  |  |  |
| Sköldstam 1992 | v |  |  |  |  |  | v |  | v |  |  |  |  |  |  | v |  |  |  |  |  |  |  |  |  |  |  |  |  |  |  | v |  |  |  | v |  | v |  |  |  |  |  |  |  |  |  |  |  |  |  |
| Stammers 1992 | v |  |  |  |  | o |  |  |  |  |  |  |  |  |  |  |  |  | o | v |  |  |  |  |  |  |  |  |  |  |  |  |  |  |  |  |  | o |  |  |  |  |  |  |  |  |  |  |  |  |  |
| Geusens 1994 |  |  | v |  | v | v | v |  | v |  |  |  |  |  |  | v |  |  |  |  |  |  |  |  |  |  |  |  |  |  |  |  |  |  |  |  |  | o |  |  |  |  |  |  |  |  |  |  |  |  |  |
| Kremer 1995 |  |  | v |  | v | v |  |  |  |  |  |  |  |  | v | v |  |  |  |  |  |  |  |  |  |  |  |  |  |  |  |  |  |  |  |  | v |  |  | v |  |  |  |  |  |  |  |  |  |  |  |
| Vargová 1998 |  |  |  |  |  |  |  |  | v |  |  |  |  |  |  |  |  |  |  |  |  |  |  |  |  |  |  |  |  |  |  |  |  |  |  | (v) |  |  |  |  |  |  |  |  |  |  |  |  |  |  |  |
| Volker 2000 | v |  | v |  |  | v |  |  |  |  |  |  |  |  |  |  |  | v |  |  |  |  |  |  |  |  |  |  |  |  |  |  |  |  |  | v |  | v |  |  |  |  |  |  |  |  |  |  |  |  |  |
| Adam 2003 | v |  | v |  |  |  |  |  | v |  |  |  |  |  |  | o |  |  |  |  |  |  |  |  |  |  |  |  |  |  |  |  |  |  |  | v | v | v |  |  |  |  |  | v |  | v | v | v | v | v |  |
| Lau 2004 | v |  |  |  |  |  |  |  | v |  |  |  |  |  |  |  |  |  |  |  |  |  |  |  |  |  |  |  |  | v |  |  |  |  |  | v |  | v |  |  |  |  |  |  |  |  |  |  |  |  |  |
| Sundrarjun 2004 | v |  | v |  |  |  |  |  |  |  |  |  |  |  |  |  | v |  |  |  |  |  |  |  |  |  |  |  |  |  |  |  |  |  |  | v | v | v |  |  |  | v |  |  |  |  |  |  |  |  |  |
| Berbert 2005 |  |  |  |  | v |  | v |  |  |  |  |  |  |  |  | v |  | v |  |  |  |  |  |  |  |  |  | v | v |  |  |  |  |  |  | v |  | v |  |  |  |  |  |  |  |  |  |  |  |  |  |
| Madland 2006 | v |  | v |  |  |  |  |  |  |  |  |  |  |  |  |  | v |  |  |  |  |  |  |  |  |  |  |  |  |  |  |  |  |  |  |  |  | v | v |  |  |  |  |  |  |  |  |  |  |  |  |
| Deutsch 2007 |  |  |  |  |  |  |  |  |  | v |  |  |  |  |  |  |  |  |  |  |  |  |  | v |  |  |  |  |  |  |  |  |  |  |  | v |  |  |  |  |  |  |  |  |  |  |  |  |  |  |  |
| Galarraga 2008 | v |  |  |  |  |  |  |  | v |  |  |  |  |  |  | v |  | v |  |  |  |  |  |  |  |  |  |  |  |  |  |  |  |  |  | v |  |  |  |  |  |  |  |  |  |  |  |  |  |  |  |
| Das Gupta 2009 | v |  | v |  |  |  |  |  |  |  |  |  | v |  |  | v |  |  |  |  |  |  |  |  |  |  | v |  |  |  |  |  |  |  |  | v |  | v |  |  |  |  |  |  |  |  |  |  |  |  |  |
| Gruenwald 2009 | v |  |  |  |  |  |  |  |  | v | v | v |  |  |  |  |  |  |  |  |  |  |  | v |  |  |  |  |  |  |  |  |  |  |  |  |  |  |  |  |  |  |  |  |  |  |  |  |  |  |  |
| Park 2013 | v |  |  |  |  |  |  |  | v |  |  |  |  |  |  |  |  | v |  |  |  |  |  |  |  |  |  |  |  |  |  |  |  |  |  | v | v |  |  |  |  | v |  | v |  |  |  |  |  | v |  |
| Araújo 2014 |  |  | v |  |  |  |  |  |  |  |  |  |  |  |  |  |  |  |  |  |  |  |  |  |  |  |  |  |  |  |  |  |  |  |  | v |  | v |  |  |  |  |  |  |  |  |  |  |  |  |  |
| Stebbings 2014 | v | v |  |  |  |  |  |  | v | v |  |  |  |  |  |  |  | v |  |  |  | v |  | v |  |  |  |  |  |  |  |  | v | v | v | v |  |  |  |  |  |  |  |  |  |  |  |  |  |  |  |
| Fu 2015 |  |  | v |  |  |  |  |  |  |  |  |  |  |  |  |  |  |  |  |  |  |  |  |  |  |  |  |  |  |  |  |  |  |  |  | v | v | v |  | v |  | v | v | v |  |  |  |  |  |  |  |
| **Trials with incomplete data or no data for pain** | | | | | | | | | | | | | | | | | | | | | | | | | | | | | | | | | | | | | | | | | | | | | | | | | | | |
| Kremer 1986 | o |  | o |  |  |  |  |  |  |  |  |  |  |  |  |  |  |  |  |  |  |  |  |  | o |  |  |  |  |  |  |  |  |  |  |  |  |  |  |  |  |  |  |  |  |  |  |  |  |  |  |
| Darlington and Ramsey 1987 |  |  | o |  |  |  |  | o |  |  |  |  |  |  |  | o |  |  |  |  | o |  |  |  |  |  |  |  |  |  |  |  |  |  |  |  |  | o |  |  |  |  |  |  |  | o |  |  |  |  |  |
| Harnandez-Cruz 1998 | o |  | o |  |  |  | o |  |  |  |  |  |  |  |  |  |  | o |  |  |  |  |  |  |  | o |  |  |  |  |  |  |  |  |  |  |  |  |  |  |  |  |  |  |  |  |  |  |  |  |  |
| Kremer 1988 |  |  | o |  |  |  |  |  |  |  |  |  |  |  |  |  |  |  |  |  |  |  |  |  |  |  |  |  |  |  |  |  |  |  |  |  |  |  |  |  |  |  |  |  |  |  |  |  |  | o |  |
| Kremer 1993 |  |  | o |  | o | o |  |  |  |  |  |  |  |  |  |  |  |  |  |  |  |  |  |  |  |  |  |  |  |  |  |  |  |  |  |  |  |  |  |  |  |  |  |  |  |  |  |  |  |  |  |
| Lau 1993 | o |  |  |  |  |  | o |  | v |  |  |  |  |  |  | o |  |  |  |  |  |  |  |  |  |  |  |  |  |  |  |  |  |  |  | o |  | v |  |  |  |  |  |  |  |  |  |  |  |  |  |
| Lau et al. 1995 | o |  |  |  |  |  | o |  |  |  |  |  |  |  |  | o |  |  |  |  |  |  |  |  |  |  |  |  |  |  |  |  |  |  |  | o |  | o |  |  |  |  |  |  |  |  |  |  |  |  |  |
| Kolahi 2010a |  |  |  |  |  |  |  |  |  |  |  |  |  |  |  |  |  |  |  |  |  |  |  |  |  |  |  |  |  |  |  |  |  |  |  | v | v |  |  |  |  |  |  |  |  |  |  |  |  |  |  |
| Kolahi 2010b |  |  | o |  |  |  |  |  |  |  |  |  |  | o |  |  |  |  |  |  |  |  |  |  |  |  |  |  |  |  |  |  |  |  |  |  |  |  |  |  |  |  |  |  |  |  |  |  |  |  |  |
| Dawczynski 2011 |  |  |  |  |  |  |  |  |  |  |  |  |  |  |  |  |  |  |  |  |  |  |  |  |  |  |  |  |  |  |  |  |  |  |  |  |  |  |  |  |  |  |  |  |  |  |  |  |  |  |  |
| Reed 2014^a^ |  |  |  |  |  |  |  |  | v |  |  |  |  |  |  |  |  |  |  |  |  |  |  |  |  |  |  |  |  |  |  |  |  |  |  |  |  |  |  |  |  |  |  |  |  |  |  |  |  |  |  |
| (Olendzki 2011)^a^ |  |  |  |  |  |  |  |  |  |  |  |  |  |  |  |  |  |  |  |  |  |  |  |  |  |  |  |  |  |  |  |  |  |  |  | o |  | o |  |  |  |  |  |  |  |  |  |  |  |  |  |
| Yazdanpanah 2014 | o | o |  |  |  |  |  |  |  |  |  |  |  |  |  |  |  |  |  |  |  |  |  | o |  |  |  |  |  |  |  |  |  |  |  |  |  |  |  |  |  |  |  |  |  |  |  |  |  |  |  |

| Reference | **Other** | | | | | | | | | | | | | | | | | | | | | | | | | | | | | | | | | | | | | | | | | | |
| --- | --- | --- | --- | --- | --- | --- | --- | --- | --- | --- | --- | --- | --- | --- | --- | --- | --- | --- | --- | --- | --- | --- | --- | --- | --- | --- | --- | --- | --- | --- | --- | --- | --- | --- | --- | --- | --- | --- | --- | --- | --- | --- | --- |
|  | Swollen joint count | Joint swelling index | Joint circumference, hands | DAS28 | Morning stiffness | WOMAC for stiffness/total WOMAC | Patient’s global assessment of arthritis activity | Physician’s global assessment of arthritis activity | Patient’s global assessment of disease | Physician’s global assessment of disease | Diastolic BP, mm Hg | Systolic BP, mm Hg | CDAI | Dietary intake | Patient‘s impression of their RA condition | EVA GH | Subjective response | Global efficacy evaluation | Disease activity score | Glucocorticoids requirement | Physical role (SF-36 part) | General health (SF-36 part) | Vitality (SF-36 part) | Social functioning (SF-36 part) | Emotional role (SF-36 part) | Mental health (SF-36 part) | COKS (Chinese Oxford Score) | CAIMS-2SF, dif. domain | No. patients using diff. types of medicine (excl NSAID) | Medicine requirement (e.g., glucocorticoids) | Erosions score | Joint space narrowing score | Combined score (radiograph) | Pulse | Breathing rate | Body temperature | Weight | Waist circumference | Quality of Life | Cholesterol (Total, HDL- and/or LDL cholesterol) | Fatty acids or triacylglycerides | RF | Other laboratory tests |
| Belch 1986 |  |  |  |  | o |  |  |  |  |  |  |  |  |  |  |  | o |  |  |  |  |  |  |  |  |  |  |  |  |  |  |  |  |  |  |  |  |  |  |  |  |  | o |
| Belch 1988 |  |  |  |  | v |  |  |  |  |  |  |  |  |  |  |  | v |  |  |  |  |  |  |  |  |  |  |  |  |  |  |  |  |  |  |  |  |  |  |  |  | v | v |
| Cleland 1988 | v |  |  |  | v |  |  |  |  |  |  |  |  |  |  |  |  |  |  |  |  |  |  |  |  |  |  |  |  |  |  |  |  |  |  |  |  |  |  | v |  |  | o/v |
| Stammers 1989 |  |  |  |  |  |  |  |  |  |  |  |  |  |  |  |  |  |  |  |  |  |  |  |  |  |  |  |  |  |  |  |  |  |  |  |  |  |  |  |  |  |  |  |
| Kremer 1990 | v |  |  |  | v |  | v | v |  |  |  |  |  |  |  |  |  |  |  |  |  |  |  |  |  |  |  |  |  |  |  |  |  |  |  |  |  |  |  |  |  | o | o/v |
| Tulleken 1990 | v | v |  |  | v |  |  |  |  |  |  |  |  |  |  |  |  |  |  |  |  |  |  |  |  |  |  |  |  |  |  |  |  |  |  |  |  |  |  |  |  | v | v |
| van der Tempel 1990 |  | v |  |  | v |  |  |  |  |  |  |  |  |  |  |  |  |  |  |  |  |  |  |  |  |  |  |  |  |  |  |  |  |  |  |  |  |  |  |  | v | v | v |
| Espersen 1992 |  |  |  |  |  |  |  |  |  |  |  |  |  |  |  |  |  |  |  |  |  |  |  |  |  |  |  |  |  |  |  |  |  |  |  |  |  |  |  |  |  |  |  |
| Kjeldsen-Kragh 1992 | v |  |  |  | v |  |  |  | v | v |  |  |  |  |  |  |  |  |  |  |  |  |  |  |  |  |  |  |  |  |  |  |  |  |  |  |  |  |  | v | v |  |  |
| Magaró 1992 |  |  |  |  | v |  |  |  |  |  |  |  |  |  |  |  |  |  |  |  |  |  |  |  |  |  |  |  |  |  |  |  |  |  |  |  |  |  |  |  |  |  | v |
| Nielsen 1992^b^ |  | v |  |  | v |  |  |  |  |  |  |  |  |  |  |  |  |  |  |  |  |  |  |  |  |  |  |  |  |  |  |  |  |  |  |  |  |  |  |  |  |  |  |
| (Faarvang 1994)^b^ |  | v |  |  | v |  |  |  |  |  |  |  |  |  |  |  |  |  |  |  |  |  |  |  |  |  |  |  |  |  |  |  |  |  |  |  |  |  |  |  |  |  |  |
| Sköldstam 1992 |  |  |  |  |  |  | v | v |  |  |  |  |  | v |  |  |  |  |  |  |  |  |  |  |  |  |  |  |  |  |  |  |  |  |  |  | v |  |  | v | v |  |  |
| Stammers 1992 |  |  |  |  |  |  |  |  |  |  |  |  |  |  |  |  |  |  |  |  |  |  |  |  |  |  |  |  |  |  |  |  |  |  |  |  |  |  |  |  |  |  | o |
| Geusens 1994 |  |  |  |  | o |  | v | v |  |  |  |  |  |  |  |  |  |  |  |  |  |  |  |  |  |  |  |  |  |  |  |  |  |  |  |  |  |  |  |  |  | v |  |
| Kremer 1995 | v |  |  |  | v |  | v | v |  |  | v | v |  |  |  |  |  |  |  |  |  |  |  |  |  |  |  |  |  |  |  |  |  |  |  |  |  |  |  |  |  |  |  |
| Vargová 1998 |  |  |  |  |  |  |  |  |  |  |  |  |  |  |  |  |  |  |  |  |  |  |  |  |  |  |  |  |  |  |  |  |  |  |  |  |  |  |  |  |  |  |  |
| Volker 2000 | v |  |  |  | v |  | v | v |  |  |  |  |  |  |  |  |  |  |  |  |  |  |  |  |  |  |  |  |  |  |  |  |  |  |  |  |  |  |  |  | v |  |  |
| Adam 2003 | v |  |  |  |  |  | v | v |  |  |  |  |  | v |  |  |  |  |  | v |  |  |  |  |  |  |  |  |  |  |  |  |  |  |  |  |  |  |  | v | v |  | v |
| Lau 2004 |  |  |  |  |  |  | v | v |  |  | v | v |  |  |  |  |  |  |  |  |  |  |  |  |  |  | v | v |  |  |  |  |  |  |  |  |  |  |  |  |  |  | v |
| Sundrarjun 2004 | v |  |  |  |  |  |  |  | v |  |  |  |  | v |  |  |  |  |  |  |  |  |  |  |  |  |  |  |  |  |  |  |  |  |  |  |  |  |  |  | v |  |  |
| Berbert 2005 |  |  |  |  | v |  | v |  |  |  |  |  |  |  |  |  |  |  |  |  |  |  |  |  |  |  |  |  |  |  |  |  |  |  |  |  |  |  |  |  |  | v | v |
| Madland 2006 | v |  |  |  |  |  |  |  | v |  |  |  |  |  |  |  |  |  |  |  |  |  |  |  |  |  |  |  |  |  |  |  |  |  |  |  |  |  |  | v | v |  | v |
| Deutsch 2007 |  |  |  |  |  | v |  |  |  |  |  |  |  |  |  |  |  |  |  |  |  |  |  |  |  |  |  |  |  |  |  |  |  |  |  |  |  |  |  |  |  |  |  |
| Galarraga 2008 |  |  |  | v | v |  |  |  |  |  |  |  |  |  |  |  | v |  |  |  |  |  |  |  |  |  |  |  |  |  |  |  |  |  |  |  |  |  |  |  |  |  |  |
| Das Gupta 2009 | v |  |  | v | v |  |  |  | v |  |  |  |  |  |  |  |  |  |  |  | v | v | v | v | v | v |  |  |  |  |  |  |  |  |  |  |  |  |  |  |  |  |  |
| Gruenwald 2009 |  |  |  |  | v | v |  |  |  |  |  |  |  |  |  |  |  | v |  |  |  |  |  |  |  |  |  |  |  |  |  |  |  |  |  |  |  |  |  |  |  |  | v |
| Park 2013 |  |  |  |  | v |  |  |  | v | v | v | v |  |  |  |  |  |  |  |  |  |  |  |  |  |  |  |  |  | v |  |  |  | v | v | v | v | v |  | v | v |  | v |
| Araújo 2014 | v |  |  | v |  |  |  |  |  |  |  |  |  |  |  | v |  |  |  |  |  |  |  |  |  |  |  |  |  |  |  |  |  |  |  |  |  |  |  |  |  |  |  |
| Stebbings 2014 |  |  |  |  |  | v | v | v |  |  | v | v |  |  |  |  |  |  |  |  |  |  |  |  |  |  |  |  |  | v |  |  |  |  |  |  |  |  | v | v | v |  |  |
| Fu 2015 | v |  |  | v | v |  | v | v |  |  | v | v | v | v |  |  |  |  |  |  |  |  |  |  |  |  |  |  |  |  |  |  |  |  |  |  |  |  |  |  | v | v | v |
| **Trials with incomplete data or no data for pain** | | | | | | | | | | | | | | | | | | | | | | | | | | | | | | | | | | | | | | | | | | | |
| Kremer 1986 | o |  |  |  |  |  |  |  |  |  |  |  |  |  |  |  |  |  |  |  |  |  |  |  |  |  |  |  |  |  |  |  |  |  |  |  |  |  |  |  |  |  |  |
| Darlington and Ramsey 1987 |  |  |  |  | o |  |  |  |  |  |  |  |  |  |  |  |  |  |  |  |  |  |  |  |  |  |  |  |  |  |  |  |  |  |  |  |  |  |  |  |  |  | o |
| Harnandez-Cruz 1998 | o |  |  |  |  |  | o | o |  |  |  |  |  |  |  |  |  |  | o |  |  |  |  |  |  |  |  |  |  |  |  |  |  |  |  |  |  |  |  |  |  |  |  |
| Kremer 1988 | o |  | o |  |  |  |  |  |  |  |  |  |  |  |  |  |  |  |  |  |  |  |  |  |  |  |  |  |  |  |  |  |  |  |  |  |  |  |  |  |  |  |  |
| Kremer 1993 |  |  |  |  | o |  | o | o |  |  |  |  |  |  |  |  |  |  |  |  |  |  |  |  |  |  |  |  |  |  |  |  |  |  |  |  |  |  |  |  |  |  |  |
| Lau 1993 |  |  |  |  | o |  |  |  |  |  |  |  |  |  | v |  |  |  |  |  |  |  |  |  |  |  |  |  |  |  |  |  |  |  |  |  |  |  |  |  | v | o | o |
| Lau 1995 |  |  |  |  | o |  |  |  |  |  |  |  |  |  |  |  |  |  |  |  |  |  |  |  |  |  |  |  |  |  |  |  |  |  |  |  |  |  |  |  | v | o | v/o |
| Kolahi 2010b | o |  |  |  |  |  |  |  |  |  |  |  |  |  |  |  |  |  | o |  |  |  |  |  |  |  |  |  |  |  |  |  |  |  |  |  |  |  |  |  |  |  |  |
| Kolahi 2010a |  |  |  |  |  |  |  |  |  |  |  |  |  |  |  |  |  |  |  |  |  |  |  |  |  |  |  |  |  |  |  |  |  |  |  |  |  |  |  |  |  |  | v |
| Dawczynski 2011 |  |  |  |  |  |  |  |  |  |  |  |  |  | v |  |  |  |  |  |  |  |  |  |  |  |  |  |  |  |  |  |  |  |  |  |  |  |  |  |  | v |  |  |
| Reed 2014 ^a^ |  |  |  | v |  |  |  |  |  |  |  |  | v |  |  |  |  |  |  |  |  |  |  |  |  |  |  |  | v |  | v | v | v |  |  |  |  |  |  |  |  |  |  |
| (Olendzki 2011)^a^ |  |  |  |  |  |  |  |  |  |  | o | o |  | v |  |  |  |  |  |  |  |  |  |  |  |  |  |  |  |  |  |  |  |  |  |  | o |  |  | o | v |  | v |
| Yazdanpanah 2014 |  |  |  |  | o | o |  |  |  |  |  |  |  |  |  |  |  |  |  |  |  |  |  |  |  |  |  |  |  |  |  |  |  |  |  |  |  |  |  |  |  |  |  |

v, indicates fully reported results; v indicates that the outcome has been used in the main meta-analysis of pain, function, or inflammation; (v) indicates that it is unclear if results are baseline, final values or changes; o indicates only p-value reported or if result is significant or not, or only results reported for one group. BP = blood pressure; C3 = Complement component; C4 = Complement component 4; C3d = complement component 3 d; CAIMS-2SF = Chinese Arthritis Impact Measurement Scale-2 Short Form; CDAI = clinical disease activity index; COKS = Chinese Oxford Score; CRP = c-reactive protein; DAS28 = disease activity score 28; diff. = different; ESR = erythrocyte sedimentation rate; EVA GH = escala analógica visual, i.e., VAS general health; HAQ = health assessment qustionaire; HDL = high-density lipoprotein; IL = interleukin; LDL = low-density lipoprotein; LTB = lymphotoxin beta; MHAQ = modified health assessment questionaire; NSAID = non stroidal anti-inflammatory drug; ph = physician; PM = post meridiem = i.e., morning; PG-M = phosphoglycomutase; PGE = prostaglandin E; RA = rheumatoid arthritis; RF = rheumatorid factor; SF-36 = short form (36) health survey; TNF-alpha = tumor necrosis factor alpha; TXB2 = thromboxane B2; VAS = visual analogue scale; WOMAC = Western Ontario and McMaster Universities Arthritis Index. ^a^ Multiple publication; ^b^ Multiple publication; * According to revised criteria of the ACR; ** a meassure for inflammation in intestine. ^a^ Multiple publication; ^b^ Multiple publication.

**Table S3.** Bias assessment table.

| **For Bias table** | **Random Sequence Generation (Selection Bias)** | **Allocation Concealment (Selection Bias)** | **Blinding of Participants (Performance Bias)** | **Blinding of Personnel (Performance Bias)** | **Blinding of Outcome Assessment (Detection bias)** | **Incomplete Outcome Data (Attrition Bias)** |
| --- | --- | --- | --- | --- | --- | --- |
| Belch et al. 1986 | **Inadequate**  Comment: Does not mention randomization. Only an abstract is available.  Quote: “We gave 11 RA patients 450mgs GLA/day (EPO, Efamol Ltd), 11 patients 240mgs EA and 480mgs GLA/day (EPO/fish oil, Efamol Ltd) and 12 patients an inert oil (placebo).” | **Inadequate**  Comment: Not addressed. | **Unclear**  Comment: Have used placebo. Does not address if the blinding was effective.  Quote: “in inert oil (placebo)” “A double blind study”. | **Unclear**  Comment: Insufficient information.  Quote: “A double blind study”. | **Unclear**  Comment: Insufficient information.  Quote: “A double blind study”. | **Unclear**  Comment: Only reports that 11 got EPO/FO, 11 got EPO and 12 got placebo. Does not mention dropouts, which is likely to be present in a 1½ year study. Unclear which analyses ware used. |
| Belch et al. 1988 | **Unclear**  Comment: Insufficient information. Suspicion of inadequate randomization, since the patients were not evenly distributed across the groups, however they do mention randomization.  Quote: “They [the capsules] were issued to the patients in a randomized double blind fashion.” “Sixteen patients received EPO treatment (…) 15 received EPO/fish oil capsules (…) 18 patients received 12 capsules/day of placebo (liquid paraffin).” | **Unclear**  Comment: Not addressed. | **Adequate**  Comment: Have used placebo with similar appearance as treatment. Does not address if the blinding was effective, but the blinding is unlikely to be broken.  Quote: “All three types of capsules were supplied by Efamol Ltd and were visually identical.”, “a double blind placebo controlled study” | **Unclear**  Comment: Does not specify, however from 12 to 15 months, the investigators were not blinded.  Quote: “As this was part of the study design investigators were aware of the treatment in all patients from 12 to 15 months; the patients, however, remained blinded.” “a double blind placebo controlled study” | **Unclear**  Comment: Does not specify, however from 12 to 15 months, the investigators were not blinded.  Quote: “As this was part of the study design investigators were aware of the treatment in all patients from 12 to 15 months; the patients, however, remained blinded.” “a double blind placebo controlled study” | **Adequate**  Comment: Forty-nine were randomized, 15 to EPO/FO, 16 to EPO, and 18 to placebo. There were 2, 3 and 10 dropouts in the groups respectively, however all were included in the analyses (i.e., ITT-analyses were used).  Quote: “Table 2 shows the number of patients withdrawn from the study by 12 months. One patient in the EPO group and two in the EPO/fish oil group were withdrawn owing to increasing symptoms of RA, compared with 10/18 of the placebo patients (both *p <* 0–001, Mann-Whitney). The results from all patients who were withdrawn were analysed throughout the study on an intention to treat basis.” |
| Cleland et al. 1988 | **Unclear**  Comment: Does not describe a random component.  Quote: “Following and initial assessment the patients were stratified according to age, sex, disease duration and disease severity and then randomly allocated into fish oil or comparison (olive oil) treatment groups.” | **Unclear**  Comment: Not addressed. | **Adequate**  Comment: Have used placebo with similar appearance as treatment. Does not address if the blinding was effective, but the blinding is unlikely to be broken.  Quote: “The other group received a similar number daily of capsules of identical appearance containing 1 g olive oil.” | **Unclear**  Comment: Does not specify.  Quote: “The study was double blind noncrossover.” | **Unclear**  Comment: Does not specify.  Quote: “The study was double blind noncrossover.” | **Inadequate**  Comment: Sixty randomized, 30 in each group. Seven in each group were withdrawn due to non-compliance, pharmacy error or dropout, but no distribution among groups stated. Likely that PP-analyses were used.  Quote: “Seven patients in each group dropped out or were eliminated for non-compliance (including one patient in the olive oil treated group who was omitted from analysis due to a pharmacy error in the third month of the study).” |
| Stammers et al. 1989 | **Inadequate**  Comment: Insufficient information. Does not mention randomization.  Quote: “We studied 21 women and 5 men” | **Unclear**  Comment: Not addressed. | **Unclear**  Comment: Have used placebo, but it is only stated that it is oil. Does not address if the blinding was effective.  Quote: “They were then given 10 ml of EPA or placebo oil daily”. | **Inadequate**  Comment: Insufficient information. Does not mention blinding. Possible that there was no blinding, since it was a pilot study. | **Inadequate**  Comment: Insufficient information. Does not mention blinding. Possible that there was no blinding, since it was a pilot study. | **Unclear**  Comment: Twenty-six included. Does not state allocation or dropouts. Unclear which analyses were used. |
| Kremer et al. 1990 | Unclear  Comment: Insufficient information.  Quote: “randomized, double-blind, parallel study”, “Patients were randomized for age, sex, treatment with slow-acting antirheumatic drugs (SAARDs), and disease severity.” | Unclear  Comment: Not addressed. | Inadequate  Comment: Have used placebo. Does not address if the blinding was effective, but the number of capsules was not the same for the three groups, i.e., it is likely that the blinding have been broken.  Quote: “1 group of RA patients who ingested nine olive oil capsules/day (...) one group of RA patients who took a “low dose” of fish oil, which consisted of 27 mg/kg/day EPA and 18 mg/kg/day DHA; and one group of RA patients who took a “high dose” of fish oil, 54 mg/kg/day EPA and 36 mg/kg/day DHA”. | Inadequate  Comment: States that the physicians were blinded, but it is likely that the rest of the personnel were not blinded, since the number of capsules given to the three groups was not the same.  Quote: “double-blind, parallel study.” “All three groups ingested olive oil supplements during weeks 24-30. Patients were not aware of the change in dietary supplement during this period. Physicians were aware of the change, but remained blinded to the original dietary supplement group assignments through the 30 weeks of study” | Adequate  Comment: States that the physicians were blinded.  Quote: “All three groups ingested olive oil supplements during weeks 24-30. Patients were not aware of the change in dietary supplement during this period. Physicians were aware of the change, but remained blinded to the original dietary supplement group assignments through the 30 weeks of study” | Inadequate  Comment: Sixty-four randomized, 22 to low dose, 19 to high dose, and 23 to olive oil. Fifteenwithdrawals including 6 dropouts and 1 noncompliance, leaving 20, 12 and 17 for the analysis, i.e., PP-analyses were probably used for all outcomes. The authors evaluates that the results of the olive oil group may be biased because of the withdrawals.  Quote: “ Any patient in either fish oil group who failed to demonstrate an increase in plasma EPA levels was not included in the data analysis.” “The reasons for the nine administrative withdrawals included a (…) lack of evidence of compliance” |
| Tulleken et al. 1990 | **Unclear**  Comment: Insufficient information.  Quote: “were randomly assigned by the pharmacy department of the hospital to receive either the fish oil supplement or the coconut oil supplement” | **Adequate**  Comment: Third party assigns patients.  Quote: “randomly assigned by the pharmacy department of the hospital” | **Adequate**  Comment: Have used placebo with similar appearance as treatment and with fish flavor added. Does not address if the blinding was effective, but it is unlikely that the blinding has been broken.  Quote: “The supplements were contained in identical capsules. Four capsules were given three times a day for 3 months. To prevent identification of the coconut oil, fish flavor was added to the capsules.” | **Unclear**  Comment: Insufficient information. Does not mention blinding. | **Unclear**  Comment: Insufficient information. Does not mention blinding. | **Inadequate**  Comment: Twenty-eight randomized, 14 in each group. One dropout due to intolerance. Thirteeen from FO group and 14 from control are included in the analyses, i.e., CC-analyses were used.  Quote: “One patient who received fish oil discontinued the supplementation within 1 week of beginning treatment, because of intolerance of the size and number of capsules.” |
| Van der Tempel et al. 1990 | **Unclear**  Comment: Insufficient information. Not specified how many starts with intervention and control.  Quote: “were randomly allocated to receive each day either 12 capsules of fractionated fish oil or fractionated coconut oil” | **Unclear**  Comment: Not addressed | **Adequate**  Comment: Have used placebo with similar appearance as treatment. Does not address if the blinding was effective, but the blinding is unlikely to have been broken.  Quote: “either 12 capsules of fractionated fish oil or fractionated coconut oil, flavored with fish aroma, as placebo” | **Adequate**  Comment: Reports that blinding was done.  Quote: “Patients and doctors were blinded to treatment assignment during the entire study.” | **Adequate**  Comment: Reports that blinding was done, assuming that doctors also are outcome assessors.  Quote: “Patients and doctors were blinded to treatment assignment during the entire study.” | **Unclear**  Comment: Sixteen randomized, but not specified how many starts with intervention and control. Two dropouts. Does not specify if the dropouts were included in the analyses or not. |
| Espersen et al. 1992 | **Inadequate**  Comment: Insufficient information. Does not mention random allocation.  Quote: “were randomly included in the study; they were allowed a daily dietary supplement of either 3.6 g *n*-3 PUFA (18 patients) or placebo (14 patients) for 12 weeks.” | **Unclear**  Comment: Not addressed. | **Unclear**  Comment: Uses control treatment, but does not specify supplementation formulation. Does not address if the blinding was effective.  Quote: “double-blind study”. | **Unclear**  Comment: Insufficient information. Quote: “double-blind study”. | **Unclear**  Comment: Insufficient information.  Quote: “double-blind study”. | **Inadequate**  Comment: Does not mention any dropouts or excluded subjects, or how many was randomized. |
| Kjeldsen-Kragh et al. 1992 | **Unclear**  Comment: Insufficient information.  Quote: “double blind, placebo controlled trial.” | **Unclear**  Comment: Not addressed. | **Unclear**  Comment: Have used placebo, but does not mention anything about similar appearance or if the blinding was effective. | **Unclear**  Comment: Insufficient information.  Quote: “double blind, placebo controlled trial.” | **Unclear**  Comment: Insufficient information.  Quote: “double blind, placebo controlled trial.” | **Inadequate**  Comment: Seventy-nine randomized, 26 to stepwise FO, 25 to FO, and 28 to placebo. There were 3, 5 and 4 respectively withdrawn due to noncompliance, leaving 23, 20 and 24 respectively for the analysis. Seven dropouts due to side effects were kept in the data analyzed. PP-analyses were used. Quote: Criteria for compliance prespecified.  Quote: “Of the remaining 67 patients, all were included in the final analysis. Seven of these experienced side effects necessitating early termination.” |
| Magaró et al. 1992 | **Unclear**  Comment: Insufficient information.  Quote: “randomly divided into two groups.” | **Unclear**  Comment: Not addressed. | **Inadequate**  Comment: Not blinded, since no placebo treatment was used. | **Inadequate**  Comment: Insufficient information. Does not mention blinding. | **Inadequate**  Comment: Insufficient information. Does not mention blinding. | **Unclear**  Comment: Twenty randomized, 10 in each group. Does not state dropouts or withdrawals or number included in the analysis. It is unclear whether ITT-analyses were used. |
| Nielsen et al. 1992 | **Unclear**  Comment: Does not mention a random component.  Quote: “Patients were randomly allocated to either(…)” “To secure an even distribution of active and control capsules between the three hospitals the capsules were randomized in blocks of 10, two of these blocks were randomly assigned to each Department.” | **Unclear**  Comment: Not addressed. | **Unclear**  Comment: Have used placebo with similar appearance and flavor. Does not report if the blinding was effective, but it is unclear if it has been broken, since belching were reported in the FO group.  Quote: “All capsules were manufactured identically and flavoured with fish oil in an attempt to prevent patients from revealing what type of treatment they received.” “No patients reported any side effects apart from slight belching among a few of those receiving the fish oil capsules.” | **Unclear**  Comment: Insufficient information.  Quote: “A double blind, randomized design with a 12-week” | **Unclear**  Comment: Insufficient information.  Quote: “A double blind, randomized design with a 12-week” | **Unclear**  Comment: Fifty-seven randomized, 29 to FO and 28 to control. Exclusion criteria were prespecified, and there were six withdrawals, hence PP-analyses were used. However, they did reanalyze some of their data with ITT-analyses.  Quote: “patients were excluded if dosage of NSAID or glucocorticoids had been changed within the last month and in case of SAARD treatment within the last 4 months prior to entry of the study”, “Additionally we reanalyzed our data based on the intention to treat principle. For drop-outs unrelated to treatment we extrapolated the last observed values and for treatment-related dropouts we used minimum value extra polation [19].” |
| Sköldstam et al. 1992 | **Unclear**  Comment: Insufficient information.  Quote: “The patients were randomly allocated to (…)” | **Unclear**  Comment: Not addressed. | **Inadequate**  Comment: Have used placebo, however, the fish oil provided a taste of fish, i.e., the blinding is likely to have been broken.  Quote: “The patients and the investigators were all unaware of the treatment assignment during the entire study.”, “Twenty of the 22 patients on fish oil and two of the control patients identified a taste of fish, which they associated with the intake of capsules.” | **Adequate**  Comment: States that the investigators were blinded.  Quote: “The patients and the investigators were all unaware of the treatment assignment during the entire study.” | **Adequate**  Comment: States that the investigators were blinded.  Quote: “The patients and the investigators were all unaware of the treatment assignment during the entire study.”, “However, since the information about the taste experience was obtained after the conclusion of the study, the investigators were unaware of this when doing the patient assessment” | **Inadequate**  Comment: Forty-six randomized, 23 in each group. Exclusion criteria were prespecified resulting in 3 withdrawals, 2 from control and 1 from FO, hence it is likely that PP-analyses were used.  Quote: “They were free to adjust the dose of their NSAID medication from day to day, governed by their need of pain relief, but not to shift between different NSAID.” “After 3 months two control group patients and one patient taking fish oil showed more active disease and asked for more effective medication, and they were excluded from the study.” |
| Stammers et al. 1992 | **Unclear**  Comment: Insufficient information.  Quote: “were randomly allocated to receive” | **Unclear**  Comment: Not addressed. | **Inadequate**  Comment: Have used placebo. Does not address if the blinding was effective. The blinding is likely to have been broken since the patients did not receive the oil in capsules.  Quote: “double-blind, placebo controlled trial” “10 ml olive oil placebo or 10 ml cod liver oil”. | **Unclear**  Comment: Insufficient information.  Quote: “double-blind, placebo controlled trial”. | **Unclear**  Comment: Insufficient information.  Quote: “double-blind, placebo controlled trial”. | **Inadequate**  Comment: Eighty-six randomized, 44 to cod liver oil and 42 to control. Twenty-two dropouts and withdrawals excluded from analysis, 13 in cod liver oil group and 9 in control group. The analysis included 29 in each group. Does not address the remaining six. Reports same number of patients in each groups at all time points in Table 3, which seems unlikely. PP-analyses were used.  Quote: “Twenty two patients failed to complete the study - 13 in the group receiving fish oil and nine receiving olive oil. The reasons for withdrawal included: non-compliance (5 receiving cod liver oil, 3 olive oil); concomitant illness (5 cod liver oil, 2 olive oil); and gastrointestinal upset (1 cod liver oil, 3 olive oil). No reason for withdrawal was stated for two patients taking fish oil and one patient taking olive oil.” |
| Geusens et al. 1994 | **Unclear**  Comment: Insufficient information.  Quote: “were randomly assigned to one of the following three daily regimens: (…)”, “randomized study”. | **Unclear**  Comment: Not addressed. | **Unclear**  Comment: Have used placebo. Does not address if the blinding was effective.  Quote: “Six capsules containing 1 gm of olive oil each (placebo), or three capsules containing 1 gm of fish oil (1.3 gm ω3) each plus three placebo capsules, or six capsules containing 1 gm of fish oil each (2.6 gm of ω3).” | **Unclear**  Comment: Insufficient information.  Quote: “A 12-Month, Double-Blind, Controlled Study”. | **Unclear**  Comment: Insufficient information.  Quote: “A 12-Month, Double-Blind, Controlled Study”. | **Inadequate**  Comment: Ninety randomized, 30 in each group. Thirty withdrawals, 9 from high dose FO, 11 from high does FO, 10 from placebo. Noncompliance was among reasons for withdrawal. Included 21, 19 and 20 in the analyses respectively, i.e., PP-analyses were used.  Quote: “Compliance with the treatment protocol was monitored by capsule counts.” |
| Kremer et al. 1995 | **Unclear**  Comment: Insufficient information.  Quote: “Patients were randomized to receive ω3 fatty acid or corn oil supplements according to age, sex, disease duration, and three categories of disease severity (…).” | **Unclear**  Comment: Not addressed. | **Inadequate**  Comment: Have used placebo, but not in the same amount as the MOS treatment, so the patients may possibly not have been blinded effectively. Does not address if the blinding was effective.  Quote: “This was a double-blind, placebo controlled, prospective study.” “Patients took either 130 mg/kg/day of ω3 fatty acids or nine capsules/day of corn oil”. | **Inadequate**  Comment: They defined the dose of MOS by weight, but corn oil was given in a fixed number of capsules, so the personnel may possibly not have been blinded. Mentions only blinding with respect to NSAID usage.  Quote: “Half of the patients were switched at week 18 and the other half at week 22 so that the investigators would not be unblinded to NSAID usage (…)” “In addition, by substituting a visually identical placebo diclofenac for the active drug, both patients and investigators could remain blinded (…)” | **Unclear**  Comment: Insufficient information.  Quote: “This was a double-blind, placebo-controlled, prospective study.” | **Inadequate**  Comment: Sixty-six randomized. The distribution among the groups and number of dropouts are unclear due to contradictory information, and no reasons for dropouts are given. Fifteen and 14 were included in the analysis for MOS vs. placebo, i.e., non-ITT-analyses are used. |
| Vargová et al. 1998 | **Unclear**  Comment: Insufficient information.  Quote: “divided by the method of random numbers into two groups” | **Unclear**  Comment: Not addressed. | **Inadequate**  Comment: Does not mention any treatment given to the control group.  Quote: “The second group served as control.” | **Inadequate**  Comment: Does not mention blinding, however only the abstract was available in English. If no placebo were used, not all of the personnel can be blinded. | **Inadequate**  Comment: Does not mention blinding, however only the abstract was available in English. | **Unclear**  Comment: The reporting suggests that 23 patients were randomized, 13 to *n*-3 PUFA treatment and 10 to control. Does not mention any withdrawals or dropouts. It is, however, unclear if true ITT-analyses have been used. |
| Volker et al. 2000 | **Unclear**  Comment: Insufficient information.  Quote: “a 15 week, randomized, placebo controlled, double blind noncrossover study”. | **Unclear**  Comment: Not addressed. | **Unclear**  Comment: Have used control treatment. Does not address if the blinding was effective.  Quote: “placebo controlled, double blind” “The control capsule was 50/50 corn/olive oil”. | **Unclear**  Comment: Insufficient information.  Quote: “double blind”. | **Unclear**  Comment: Insufficient information.  Quote: “double blind”. | **Inadequate**  Comment: Fifty randomized, 25 to fish oil and 25 to control. Twelve excluded from the analysis in each group (48%) due to predefined criteria. PP-analyses used.  Quote: “Change in treatment regime was a predetermined criterion for withdrawal from the study” “Withdrawal reasons: (…) 5. Withdrawal caused by supplement noncompliance**” “**Noncompliance was verified by capsule count at 4, 8, and 15 weeks and by plasma lipid analysis at baseline and 15 weeks”. |
| Adam et al. 2003 | **Unclear**  Comment: Patients were randomized with respect to MOS treatment. No details on the sequence generation or how many are allocated to receiving FO or placebo first.  Quote: “Patients were matched to two comparable groups for allocation to the dietary regimen. (…) The subjects of each group were randomized for allocation to placebo or verum given in a crossover design for 3 months each, with a 2-month washout period between treatments (Figure 1).” | **Unclear**  Comment: Not addressed. | **Unclear**  Comment: Have used placebo. Does not address if the blinding was effective.  Quote: “Placebo (1 g of corn oil) and verum (1 g of menhaden oil) capsules” | **Unclear**  Comment: Does not specify with respect to fish oil treatment.  Quote: “double-blind crossover study” | **Unclear**  Comment: Does not specify with respect to fish oil treatment.  Quote: “double-blind crossover study” | **Inadequate**  Comment: Sixty-eight randomized, divided into two separate diet groups of 34 each, randomized to MOS or placebo in a cross-over random order study. Does not specify how many were allocated to receiving MOS or placebo first. Eight withdrawals including six dropouts. Predefined noncompliance criteria. Thirty in each group were included in the analysis. PP-analyses are used.  Quote: “Sixty-two of the original 68 subjects completed the 8-month study. The results of two patients were omitted because, after 3 months on fish oil, no increase of EPA in erythrocyte lipids was detected and adherence to the protocol was doubtful. |
| Lau et al. 2004 | **Unclear**  Comment: Insufficient information.  Quote: “double-blind randomized placebo-controlled study”, “randomly assigned to receive either”. | **Unclear**  Comment: Not addressed. | **Unclear**  Comment: Have used placebo. Does not address if the blinding was effective.  Quote: “Patients in the placebo treatment group received the same number and schedule of capsules containing olive oil.” | **Unclear**  Comment: Insufficient information.  Quote: “double-blind randomized placebo-controlled study”. | **Unclear**  Comment: Insufficient information.  Quote: “double-blind randomized placebo-controlled study”. | **Unclear**  Comment: Eighty randomized, 40 in each group. Twelve withdrawals including noncompliance (monitors compliance by capsule count). Unclear how many were included in the analysis. Probably PP-analyses were used. |
| Sundrarjun et al. 2004 | **Unclear**  Comment: Insufficient information.  Quote: “were randomized into the fish oil, placebo and control groups.” | **Unclear**  Comment: Not addressed. | **Unclear**  Comment: Have used placebo, but does not specify which kind. Does not address if the blinding was effective.  Quote: “double-blind, placebo-controlled study”. | **Unclear**  Comment: Insufficient information.  Quote: “double-blind, placebo-controlled study”. | **Unclear**  Comment: Insufficient information.  Quote: “double-blind, placebo-controlled study”. | **Unclear**  Comment: Sixty randomized, 23 in FO and 23 in control. Twenty-five withdrawn in total (41%), 10 in FO group and control group respectively, including non-compliance. All were included in the analysis and they report doing ITT-analyses, however it is assumed that imputations were done for non-compliance, i.e., mITT-analyses were probably used.  Quote: “Thirty-five patients completed the study. The remaining 25 patients were withdrawn from the study prior to completion due to unstable medication for other illnesses (*n =* 9), inability to attend regular appointments (*n =* 13), abnormal liver function (*n =* 1) or non-compliance (*n =* 2).” |
| Berbert et al. 2005 | **Unclear**  Comment: Insufficient information.  Quote: “In this randomized, parallel design, patients were assigned to one of three groups.”, “were investigated in a parallel randomized design. Patients were assigned to one of three groups.” | **Unclear**  Comment: Not addressed. | **Inadequate**  Comment: Have used placebo. The formulation type for the soy oil is unclear, but the olive oil was administered from a flask on salads, in contrast to MO that was administered in capsules.  Quote: “(…) the first group (G1) received placebo (soy oil), the second group (G2) received 3 g/d (20 capsules) of fish oil ω-3 fatty acids, and the third group (G3) received 3 g/d of fish oil ω-3 fatty acids and 6.8 g of oleic acid (9.6 mL of olive oil).” “The oleic acid was handled in a proper plastic flask filled with extra-virgin olive oil, which was added to salads.” | **Inadequate**  Comment: The personnel were not blinded.  Quote: “clinical investigators were not blinded.” | **Inadequate**  Comment: The personnel were not blinded. However, they argue that the non-blinding did not affect the outcome assessment of “some” of the measurements.  Quote: “clinical investigators were not blinded. However, the results of some clinical (right and left handgrip strength) and laboratory (rheumatoid factor) objective measurements of disease activity agreed with those of the other indicators.” | **Inadequate**  Comment: Fifty-five randomized, 18 to FO, 20 to olive oil and 17 to soy oil. Twelve withdrawals including noncompliance, i.e., PP-analyses were done.  Quote: “Compliance was monitored at each visit by questioning patients and counting capsules.” |
| Madland et al. 2006 | **Unclear**  Comment: Insufficient information.  Quote: “included and randomly allocated to treatment with either seal oil (*n =* 22) or soy oil (*n =* 21)”. | **Unclear**  Comment: Not addressed. | **Adequate**  Comment: Have used placebo with similar appearance as treatment. Authors conclude that the blinding was effective.  Quote: “double blind controlled trial”, “Soy oil was selected as control treatment based on its similarity to seal oil in consistency and appearance” “The fact that only slightly more than 50% of the patients were able to identify which treatment they had received indicates that there was no significant blinding bias.” | **Unclear**  Comment: Insufficient information.  Quote: “double blind controlled trial”. | **Unclear**  Comment: Insufficient information.  Quote: “double blind controlled trial”. | **Inadequate**  Comment: Forty-three randomized, 22 to seal oil and 21 to soy oil. Three dropouts, 2 in seal oil and 1 in soy oil. Does not report how many was included in the analysis. Does not mention compliance, so probably CC analyses were used.  Quote: “Three subjects did not complete the trial: one because of a sports injury (seal oil), one because of intolerance to the study oil (seal oil), and one gave no reason for dropping out (soy oil).” |
| Deutsch 2007 | **Adequate**  Comment: A random component in the sequence generation is described.  Quote: “Patients were randomly assigned by a computer-generated schedule into one of two groups” | **Unclear**  Comment: Insufficient information.  Quote: “were blindly randomized in their group” | **Adequate**  Quote: Have used placebo with similar appearance as treatment. Does not address if the blinding was effective, but the blinding is unlikely to be unbroken.  Quote: “Both the NKO™ and the placebo were administered in non-distinguishable glycerin softgels.” | **Unclear**  Comment: Insufficient information.  Quote: “prospective randomized double blind clinical trial”. | **Unclear**  Comment: Insufficient information.  Quote: “prospective randomized double blind clinical trial”. | **Unclear**  Comment: Ninety randomized, 45 in each group. Three withdrawals included noncompliance. Unclear how many were included in the analysis, since no sample sizes are given for the analyses and since measures such as WOMAC pain were only used for the patients with arthritic disease (12 had only atherosclerosis) and the withdrawals is not specified within diagnosis.  Quote: “Compliance was tested by a count of softgels at each visit after 7, 14 and 30 days” |
| Galarraga et al. 2008 | **Inadequate**  Comment: Reports that the random sequence was done “manually”, which is assumed not to include a random component.  Quote: “Randomization was done separately in each of the two study centers. The randomization code was generated manually in blocks of 10.” | **Unclear**  Comment: Not addressed. | **Inadequate**  Comment: Have used placebo with similar appearance as treatment. States that the blinding possibly may not have been truly effective.  Quote: “double-blind placebo-controlled study” “Patients were randomly allocated to receive either 10 g of SSMO1 a day (10 capsules) or identical air-filled placebo capsules for 9 months.”, “awareness that the capsules were empty and dislike of the fishy taste of the capsules.” “We may have compromised the double blinding of the study by using air-filled capsules as placebo.” | **Unclear**  Comment: Insufficient information.  Quote: “double-blind, placebo-controlled study” | **Unclear**  Comment: Insufficient information.  Quote: “double-blind, placebo-controlled study” | **Adequate**  Comment: Ninety-seven randomized, 49 in treatment group and 48 in control group. Thirty-nine dropouts. Compliance assessed but none excluded due to non-compliance. Ninety-seven included in the analysis. ITT-analyses were used.  Quote: “Analysis was performed by intention to treat. The missing data were completed as follows: for the primary outcome (relative reduction of daily NSAID requirement by > 30% after 9 months) a non-completer imputation, in which non-completers were assumed to have had no reduction in NSAID consumption, was done.(…)There were no statistically significant differences in the number of withdrawals from the active and placebo groups, or in the type of adverse events that were the cause of withdrawal (P¼0.304, chi-squared test; 95% CI for difference, 30.54, 8.26).” |
| Das Gupta et al. 2009 | **Unclear**  Comment: Insufficient information.  Quote: “were equally distributed into two groups using a random allocation procedure.” | **Unclear**  Comment: Not addressed. | **Inadequate**  Comment: Does not mention blinding or that a placebo for the omega-3 capsules was used. The two groups did not receive the same number of capsules.  Quote: “One group (*n =* 50) received 25 mg indomethacin capsule (Essential Drug Company Limited, Bangladesh) daily for 12 weeks. Another group (*n =* 50) received 25 mg indomethacin capsule plus 3 g omega-3 fatty acids (capsule Meg-3, Ocean Nutrition, Canada) daily for 12 weeks.” | **Inadequate**  Comment: Insufficient information. Does not mention blinding, but the treatments are distinguishable. | **Unclear**  Comment: Insufficient information. Does not mention blinding. | **Inadequate**  Comment: One hundred randomized, 50 in each group. Nineteen dropouts, 10 in FO group and 9 in control group. No reasons stated. There were 40 and 41 included in the analyses, i.e., CC-analyses were used. |
| Gruenwald et al. 2009 | **Adequate**  Comment: Describes a random component in the sequence generation process.  Quote: “Block randomization, with a block size of four, and the randomization code were created externally using the randomization scheme EDGAR (Experimental Design Generator And Randomizer).” | **Unclear**  Comment: Not addressed. | **Adequate**  Comment: Have used placebo with similar appearance as treatment. Does not address if the blinding was effective, but the blinding is unlikely to have been broken.  Quote: “The swallowed capsules of both products did not differ in color, size, smell, or taste.” | **Unclear**  Comment: Insufficient information.  Quote: “double-blind, two-center study”. | **Unclear**  Comment: Insufficient information.  Quote: “double-blind, two-center study” | **Unclear**  Comment: One hundred and seventy-seven randomized, 90 in FO group and 87 in control group. Thirteen withdrawals including 2 dropouts. Predefined cutoffs for compliance. Reports that they did both ITT-analyses and PP-analyses. It is unclear if true ITT-analyses or mITT-analyses were used, hence if data from non-compliant patients. were imputed.  Quote: “Patients were excluded from the study if (...) uncertain compliance due to language difficulties.” “Owing to violations of the study protocol, 164 patients (group A=80, group B=84) were included in the valid case analysis set (VCAS collective).” “During the clinical trial, 23 patients experienced AEs (12.9%); in only two cases it was documented that the patients terminated the study (one from each group).” |
| Park et al. 2013 | **Adequate**  Comment: Describes a random component.  Quote: “At the trials’ coordinating center, a computer-generated block sequence balanced by the participating center randomly assigned subjects in blocks of 2.” | **Inadequate**  Have used a block size of two, which introduces bias.  Quote: “At the trials’ coordinating center, a computer-generated block sequence balanced by the participating center randomly assigned subjects in blocks of 2.” | **Adequate**  Comment: States that the participants were blinded. Have used placebo, but does not mention if it was indistinguishable from the treatment.  Quote: “All the investigators, patients and research staff were blinded to the treatment codes.” “The study was designed as a double-blind, randomized, multicenter, placebo-controlled, parallel-group trial.” | **Adequate**  Comment: States that the investigators were blinded.  Quote: “All the investigators, patients and research staff were blinded to the treatment codes.” “The study was designed as a double-blind, randomized, multicenter, placebo-controlled, parallel-group trial.” | **Adequate**  Comment: States that the investigators were blinded.  Quote: “All the investigators, patients and research staff were blinded to the treatment codes.” “The study was designed as a double-blind, randomized, multicenter, placebo-controlled, parallel-group trial.” | **Inadequate**  Comment: One hundred and nine randomized, 55 to FO and 44 to control. There were 28 dropouts, 14 from each group, due to patients own request. Eighty-one included in the analysis, hence CC-analyses were used.  Quote: “Fourteen subjects in each group dropped out: 27 for patient’s request and 1 patient due to clinical trial.” |
| Araújo et al. 2014 | **Unclear**  Comment: Insufficient information.  Quote: “randomized controlled trial.” | **Unclear**  Comment: Not addressed. | **Inadequate**  Comment: Does not state if a placebo was used. Blinding is not mentioned.  Quote: “compared to a control group”. | **Inadequate**  Comment: Does not mention blinding. If the control group does not receive any placebo, not all personnel can be blinded. | **Inadequate**  Comment: Insufficient information. Does not mention blinding. | **Inadequate**  Comment: Thirty-seven randomized. Reports 11 in *n*-3 FA group, 15 in control group, and 8 in the Mediterranean diet group, which equals only 34. Does not state withdrawals. There were 11, 15 and 8 included in the analysis respectively. Must have used non-ITT-analyses. |
| Stebbings et al. 2014 | **Unclear**  Comment: Insufficient information.  Quote: “A double-blind, randomized, placebo-controlled study” | **Adequate**  Comment: Have used opaque envelopes.  Quote: “Trial allocation was performed with allocation concealment (opaque envelopes) (…)” | **Adequate**  Comment: Have used placebo with similar appearance and odor and reports that the blinding was effective.  Quote: “Both Biolex® GLM extract and the placebo (corn oil) were produced in the form of 150mg dark brown, opaque gel capsules of identical appearance and odour in blister packs of 25 capsules.” “No evidence was found to suggest that participants could deduce which arm of the study they were in (p=0.688), with the majority (48/66, 73%) of participants believing they were in the placebo arm of the study, (24/32 in the intervention arm and 24/32 in the control arm).” [Abstract]: “All participants, assessors and investigators were blinded to the randomization.” | **Adequate**  Comment: States that the personnel were blinded.  Quote: “Researchers screening potential participants were not aware of the block sizes used. The statistician was blinded as to treatment group allocation until all main analyses, including per-protocol and subgroup analyses, were completed.” [Abstract]: “All participants, assessors and investigators were blinded to the randomization.” | **Adequate**  Comment: States that the personnel were blinded.  Quote: [Abstract]: “All participants, assessors and investigators were blinded to the randomization.” | **Inadequate**  Comment: Eighty randomized, 39 to intervention and 41 to control. Thirteen dropouts until week 12, 5 from intervention and 8 from control. Compliance was monitored resulting in one excluded patient, but it is not clear if the noncompliant patient was included in the analyses. They state that they use mITT-analyses, however they tabulate population sizes less than 80 patients, i.e., CC- or PP-analyses were used.  Quote: “ A pill count was performed by the blinded assessor at 6 and 12 weeks to gauge adherence.” “The primary analyses were conducted according to modified intention to treat principles (using all available data).” |
| Fu et al. 2015 | **Adequate**  Comment: Describes a random component in the sequence generation process.  Quote: “randomly assigned 1:1 to two groups according to a computer-generated randomization list.” | **Adequate**  Comment: The allocation was concealed by keeping it at a third party.  Quote: “The allocation groups of the patients were saved by a third party (Ningbo College of Health Sciences) and all subjects, investigators, and outcome assessors will not know the allocation groups of the patients based on the appearance of the given capsules.” | **Inadequate**  Comment: Have used placebo with similar appearance as treatment. Does not address if the blinding was effective, however five (20%) in the MOS group dropped out due to the flavors of capsules after burp, hence some of the completers may have experienced this as well.  Quote: “Patients in the second group administered orally placebo capsules (400 mg corn oil per capsule, identical in appearance to HMLE capsules) according to the same schedule.” | **Adequate**  Comment: Have used blinding of personnel. Does not address if the blinding was effective, but unlikely that the blinding could have been broken.  Quote: “The allocation groups of the patients were saved by a third party (Ningbo College of Health Sciences) and all subjects, investigators, and outcome assessors will not know the allocation groups of the patients based on the appearance of the given capsules.” | **Adequate**  Comment: Have used blinding of personnel. Does not address if the blinding was effective, but unlikely that the blinding could have been broken.  Quote: “The allocation groups of the patients were saved by a third party (Ningbo College of Health Sciences) and all subjects, investigators, and outcome assessors will not know the allocation groups of the patients based on the appearance of the given capsules.” | **Inadequate**  Comment: Fifty patients randomized, 25 to HMLE and 25 to control. There were 7 and 1 excluded from the analysis including non-compliance, i.e., PP-analyses are used. ITT-analyses were used for other measures than pain and inflammation.  Quote: “Among the enrolled patients, eight patients did not complete the study: one male and four female patients in HMLE group who complained the flavors of capsules after burp and high transportation costs, rejected to visit our outpatient clinic at month 3 or month 6; two female patients in HMLE group and one male patient in placebo were considered withdrawn from the study as they reported that they suspended taking capsules for 4–6 weeks.” |
| **Trials with incomplete data or no data for pain** | | | | | | |
| Kremer et al. 1986 | **Inadequate**  Comment: Insufficient information. Does not mention randomization.  Quote: “Thirty-six patients (pts) were entered in a 26-week (wk) double-blinded crossover study.” | **Unclear**  Comment: Not addressed. | **Unclear**  Comment: Have used placebo, but does not specify which type. Does not address if the blinding was effective.  Quote: “double-blinded crossover study.” “placebo (P) capsules”. | **Unclear**  Comment: Insufficient information.  Quote: “double-blind crossover study” | **Unclear**  Comment Insufficient information.  Quote: “double-blind crossover study” | **Unclear**  Comment: Thirty-six randomized. Does not state the distribution or number of withdrawals. Measured compliance, so PP-analyses could possibly have been used.  Quote: “Compliance was monitored by pill counts and gas chromatographic analysis of plasma lipids.” |
| Darlington & Ramsey1987 | **Unclear**  Comment: Insufficient information.  Quote: “were randomly allocated to 12 weeks of treatment” | **Unclear**  Comment: Not addressed. | **Unclear**  Comment: Have used placebo, but does not specify supplementation formulation. Does not address if the blinding was effective.  Quote: “treatment with 18 g/dag of fish oil, (Max Epa containing 18% eicosapentaenoic acid, 20:5ω3, and 12% docosahexaenoic acid, 22:63), or 18g/day of olive oil, (controls).” | **Unclear**  Comment: Not addressed. | **Unclear**  Comment: Not addressed. | **Unclear**  Comment: Thirty-five randomized. Does not mention how many in each group or exclusions from analysis. Patients may have been excluded due to non-compliance.  Quote: “compliance was checked by gas/liquid chromatography”. |
| Hernández-Cruz et al. 1988 | **Inadequate**  Comment: Insufficient information, however, reports significant differences in age and gender distribution, but not in disease duration, between the groups. With 90 patients, it would be possible to make comparable groups.  Quote: “were randomized in two groups”, “Mean age was 38.3 (σ13.3) years for patients in the ω3FA group vs. 48.1 (σ 13.6) years for the patients in the placebo group (p=0.001). Most of them were female (89% vs. 97%, respectively) p=0.04. Mean disease duration was 4.1 (σ 0.7) vs. 2.7 (σ 0.7) years, respectively *p* = 0.2.” | **Unclear**  Comment: Not addressed. | **Unclear**  Comment: Have used placebo. Does not address if the blinding was effective.  Quote: “ωFA (12 capsules/day of 1.5g of icosa and 0.7g of docopentaenoic acid) or placebo (12 capsules/day of sunflower oil)”. | **Inadequate**  Comment: Does not specify and does not mention double-blinding.  Quote: “A blinded, randomized (…)” | **Inadequate**  Comment: does not specify and does not mention double-blinding.  Quote: “A blinded, randomized (…)” | **Unclear**  Comment: Ninety randomized, 45 in ω3FA group and 45 in control group. Does not mention dropouts and states that ITT-analyses were done. It is, however, likely that there have been dropouts during the trial duration of 1 year.  Quote: “Intention to treat analysis was done.” |
| Kremer et al. 1988 | **Unclear**  Comment: Insufficient information.  Quote: “were randomized into 3 groups” | **Unclear**  Comment: Not addressed. | **Unclear**  Comment: Have used placebo. Does not address if the blinding was effective.  Quote: “placebo (olive-oil)” | **Unclear**  Comment: Insufficient information.  Quote: “A prospective double blinded randomized study” | **Unclear**  Comment: Insufficient information.  Quote: “A prospective double blinded randomized study” | **Unclear**  Comment: Fifty-five randomized. Does not state the distribution, dropouts or withdrawals. |
| Kremer et al. 1993 | **Unclear**  Comment: Insufficient information.  Quote: “Fifty pts with active RA were randomized (…)” | **Unclear**  Comment: Not addressed. | **Unclear**  Comment: Have used placebo, but unclear if the blinding was effective.  Quote: “(…) *n*-3 fatty acid or corn oil (co).” | **Unclear**  Comment: Insufficient information.  Quote: “double-blind study”. | **Unclear**  Comment: Insufficient information.  Quote: “double-blind study”. | Unclear  Comment: Does only mention the total number of patients randomized. |
| Lau et al. 1993 | **Unclear**  Comment: Insufficient information.  Quote: “were randomized to receive”. | **Unclear**  Comment: Not addressed. | **Unclear**  Comment: Have used placebo. Does not address if the blinding was effective.  Quote: “receive either 10 Maxepa or air filled placebo capsules per day for 12 months, the double-blind placebo controlled treatment phase.” | **Unclear**  Comment: Insufficient information.  Quote: “double-blind, placebo controlled treatment phase.” | **Unclear**  Comment: Insufficient information.  Quote: “double-blind, placebo controlled treatment phase.” | **Inadequate**  Comment: Sixty-four randomized, 32 in each group. There were 25 dropouts, 9 in fish oil group and 16 in control group. Does not state how many is included in the analysis, but a CC analysis is probably done. Compliance was measured, but does not state any cut-off for withdrawal or that any patients were withdrawn because of noncompliance.  Quote: “Essential fatty acid content in red cell membranes were assayed using gas liquid chromatography at months 0, 6, 12 and 15 as an assessment of compliance. Additionally, a pill count was performed at all visits.” |
| Lau et al. 1995 | **Unclear**  Comment: Insufficient information.  Quote: “were randomized to receive” | **Unclear**  Comment: Not addressed. | **Unclear**  Comment: Have used placebo. Does not address if the blinding was effective. Quote: “10 Maxepa or (…) air-filled placebo capsules per day for 6 months in a double-blind manner”. | **Unclear**  Comment: Not addressed. | **Unclear**  Comment: Not addressed. | **Adequate**  Comment: There were 45 randomized, 25 to FO and 20 to control. Reports that all completed and none were withdrawn, i.e., have used ITT-analyses. Monitored compliance.  Quote: “All patients completed the study and there were no withdrawals.” “Drug compliance was monitored by a pill count and measurement of the red blood cell membrane content of essential fatty acids using gas-liquid chromatography upon entry and on completion of the study at month 6.” |
| Kolahi, A et al. 2010a | **Unclear**  Comment: Insufficient information.  Quote: “The patients were randomly allocated to two groups”. | **Unclear**  Comment: Not addressed. | **Adequate**  Comment: Have used placebo with similar appearance. Does not address if blinding was effective.  Quote: “The placebo capsules apparently were similar to the FO capsules but without ω-3 substance.” | **Unclear**  Comment: Insufficient information.  Quote: “We designed a double blind, placebo controlled study (…)”. | **Unclear**  Comment: Insufficient information.  Quote: “We designed a double blind, placebo controlled study (…)”. | **Inadequate**  Comment: Ninety randomized, 45 in each group. Eighty-three completed, 40 from FO group and 43 from placebo group. No reasons for the seven dropouts stated. For the laboratory outcomes, 40 and 43 respectively were included in the analyses, i.e., non-ITT-analyses were used, even though they mention ITT.  Quote: “This intention to treat study (…)”. |
| Kolahi,B et al. 2010b | **Unclear**  Comment: Insufficient information.  Quote: “In this randomized double blind trial (…)” | **Unclear**  Comment: Not addressed. | **Unclear**  Comment: Have used placebo, but does not specify which type. Does not address if the blinding was effective.  Quote: “placebo of omega-3 and vitamin E” | **Unclear**  Comment: Insufficient information.  Quote: “In this randomized double blind trial (…)” | **Unclear**  Comment: Insufficient information.  Quote: “In this randomized double blind trial (…)” | **Unclear**  Comment: Ninety recruited, does not state how many in each group or the number of dropouts. |
| Dawczynski et al. 2011 | **Unclear**  Comment: Does not describe a random component in the sequence generation.  Quote: “randomization list with a block size of eight.” “In this randomized double-blind controlled clinical trial, patients were randomized to 12 weeks of treatment with one of four different supplements (allocation ratio 1:1:1:1)” | **Adequate**  Comment: Have used sealed envelopes and the personnel for enrollment were blinded.  Quote: “receiving prefabricated sealed envelopes, generated by a randomization list with a block size of eight.” “Enrollment and assignment to interventions were done only by the trial physicians (RS, UH, MV). All personal (trial physicians, study nurse, secretary) was blinded.” | **Unclear**  Comment: Indistinguishable containers were used. Does not mention appearance, and since 3 dropped out due to smell and odor (in the GLA group), blinding may have been broken.  Quote: “Capsules were handed out in glass beakers marked only with the patient number. Enrollment and assignment to interventions were done only by the trial physicians (RS, UH, MV). All personal (trial physicians, study nurse, secretary) was blinded. Study medication was labeled only with patient numbers and manufactured outside the trial site.” “Thirteen patients, representing a drop out rate of 22% did not complete the study because of the following reasons: they claimed to experience smell and odor” | **Adequate**  Comment: States that the personnel were blinded, and that the containers were labeled by a third party.  Quote: “All personal (trial physicians, study nurse, secretary) was blinded. Study medication was labeled only with patient numbers and manufactured outside the trial site.” | **Adequate**  Comment: States that the personnel were blinded.  Quote: “All personal (trial physicians, study nurse, secretary) was blinded. Study medication was labeled only with patient numbers and manufactured outside the trial site.” | **Inadequate**  Comment: Sixty randomized, 15 in each group. Thirteen withdrawals including non-compliance, 1 from FO, 2 from FO+GLA, 7 from GLA, and 3 from olive oil. 47 included in the analyses, 14 from FO, 13 from FO+GLA, 8 from GLA, and 3 from olive oil. PP-analyses are stated.  Quote: “Thirteen patients, representing a drop out rate of 22% did not complete the study because of the following reasons: they claimed to experience smell and odor, felt an insufficient relief of their symptoms, or showed other reasons of non-compliance (Figure 2).” |
| Reed et al. 2014 | **Unclear**  Comment: Insufficient information.  Quote: “18-month randomized, double-blind comparison of borage seed oil, fish oil, and the combination of both oils” “Patients were randomized to receive either (…)”  (Olendzki et al. 2011): “Patients were stratified by site (thus randomized to group within each site), and all sites were combined, resulting in non-significant differences per group.” | **Unclear**  Comment: Not addressed | **Adequate**  Comment: Have used placebo and treatments with similar appearance and coded opaque containers. Does not address if the blinding was effective, but the blinding is unlikely to have been broken.  Quote: “18-month randomized, double-blind comparison of borage seed oil, fish oil, and the combination of both oils” “All capsules were identical in appearance and color and were purchased from Bioriginal Food and ServiceCorp., Saskatoon, Canada, who shipped the capsules in coded opaque plastic bottles” | **Unclear**  Comment: Insufficient information.  Quote: “18-month randomized, double-blind comparison of borage seed oil, fish oil, and the combination of both oils” | **Unclear**  Comment: Insufficient information.  Quote: “18-month randomized, double-blind comparison of borage seed oil, fish oil, and the combination of both oils” | **Inadequate**  Comment: There were 150 randomized, 53 to FO, 45 to FO and borage oil, and 52 to borage oil. 76 dropouts. Compliance was monitored, but it is not stated as a reason for withdrawal. No results are given for the final values of pain, function and inflammation, but they state that they only use ITT-analyses. This is however not in accordance with the measures tabulated.  Quote: “Compliance, assessed by capsule counts and patient report” (Olendzki et al. 2011): “All analyses were intention to treat”. |
| Yazdanpanah et al. 2014 | **Unclear**  Comment: Insufficient information.  Quote: “randomized clinical trial” | **Unclear**  Comment: Not addressed. | **Inadequate**  Comment: Did not use placebo for the fish oil in the suitable control groups.  Quote: “in 6 groups of 19 by acetaminophen, naproxen, naproxen+omega-3, acetaminophen+omega-3, acetaminophen+naproxen and acetaminophen+naproxen+omega-3 for 6 weeks.” | **Inadequate**  Comment: Does not mention blinding. | **Inadequate**  Comment: Does not mention blinding. | **Unclear**  Comment: There were 114 randomized, 19 in each of 6 groups. Does not mention withdrawals or dropouts, or how many were included in the analyses. |

AE = adverse event; CI = confidence interval; CC = completers-case; CRP = c-reactive protein; DHA = docosahexaenoic acid; EPA = eicosapentaenoic acid; EPO = evening primrose oil; ESR = erythrocyte sedimentation rate; FO = fish oil; GLA = gamma-linolenic acid; HAQ = health assessment questionnaire; HMLE = hard-shelled mussel lipid extract; ITT = intention-to-treat; MHAQ = modified health assessment questionnaire; mITT = modified intention-to-treat; MOS = marine oil supplement; NKO = Neptune Krill Oil; n.s. = non-significant; PP = per-protocol; pt(s) = patient(s); SJC = swollen joint count; TJC = tender joint count.

**Table S4.** Outcome reporting bias assessment

| **Reference** | **Conclusion from Outcome Matrix—Any Outcomes Suspiciously Missing?** | **Comments (Reasons for Classification, Incl. Publication Status, Study Text, Level of Suspicion, Answer from Author)** | **Pain, Classification SMN** | **Pain, Classification NKS** | **Pain, Classification consensus** | **RoORB** | **Function, Classification SMN** | **Function, Classification NKS** | **Function, Classification consensus** | **RoORB** | **Inflammation, Classification SMN** | **Inflammation, Classification NKS** | **Inflammation, Classification consensus** | **RoORB** |
| --- | --- | --- | --- | --- | --- | --- | --- | --- | --- | --- | --- | --- | --- | --- |
| Belch et al. 1986 | Function, and fully reported pain and inflammation, and a typical measure for pain. | Comment: All measures are only reported partially. Only NSAID-requirement is reported as a pain outcome, which is unusual and the result is given as number of patients who significantly reduced their NSAID requirement and only results at 6 months reported (treatment duration was 12 months). Reported no change in ESR and CRP. Reports other clinical outcomes, e.g., morning stiffness, it is therefore likely that function was measured as well.  Only an abstract available. | **C** | **G** | **D** | high | **G** | **H** | **G** | high | **A** | **A** | **A** | high |
| Belch et al. 1988 | No. |  | **-** | **-** | **-** | low | **-** | **-** | **-** | low | **-** | **-** | **-** | low |
| Cleland et al. 1988 | Fully reported ESR. | Comment: ESR only reported n.s. within and between groups.. Other markers of inflammation were fully reported and with significant results. | **-** | **-** | **-** | low | **-** | **-** | **-** | low | **A** | **A** | **A** | high |
| Stammers et al. 1989 | Inflammation. | Comment: Report an unusual measure of function, “interference with activities” on VAS but they have also used VAS on pain, so it does not raise suspicion. No inflammation measure, but no other laboratory measures reported, so it does not raise suspicion, since OA was previously not seen as an inflammatory disease.  Only an abstract available. | **-** | **-** | **-** | low | **-** | **-** | **-** | low | **H** | **H** | **H** | low |
| Kremer et al. 1990 | Full reported ESR. | Comment: Other markers of inflammation fully reported, some of which are significant. ESR is only reported n.s.  Quote: “No significant changes in hemoglobin levels, Westergren ESR, or rheumatoid factor titer were observed in any group.” | **-** | **-** | **-** | low | **-** | **-** | **-** | low | **A** | **A** | **A** | high |
| Tulleken et al. 1990 | No. |  | **-** | **-** | **-** | low | **-** | **-** | **-** | low | **-** | **-** | **-** | low |
| van der Tempel et al. 1990 | Fully reported function. | Comment: Only reported that grip strength did not change. | **-** | **-** | **-** | low | **A** | **A** | **A** | high | **-** | **-** | **-** | low |
| Espersen et al. 1992 | Patient’s own assessment of pain, function, CRP and ESR. | Comment: Report only Ritchie’s articular index, which is unusual, and only within-group differences are reported, which was significant in the FO group. Unclear if other pain outcomes were measured. CRP and ESR are normally used as markers of inflammation, so it is unlikely that CRP and ESR were not measured. Ritchie’s articular index is the only clinical outcome mentioned, but other may as well have been measured. | **G** | **G** | **G** | high | **G** | **G** | **G** | high | **G** | **G** | **G** | high |
| Kjeldsen-Kragh et al. 1992 | No. |  | **-** | **-** | **-** | low | **-** | **-** | **-** | low | **-** | **-** | **-** | low |
| Magaró et al. 1992 | No. |  | **-** | **-** | **-** | low | **-** | **-** | **-** | low | **-** | **-** | **-** | low |
| Nielsen et al. 1992 | No. |  | **-** | **-** | **-** | low | **-** | **-** | **-** | low | **-** | **-** | **-** | low |
| Sköldstam et al. 1992 | No. |  | **-** | **-** | **-** | low | **-** | **-** | **-** | low | **-** | **-** | **-** | low |
| Stammers et al. 1992 | Any fully reported marker of inflammation. | Comment: Only ESR reported for inflammation, and only partial reported.  Quote: “Analysis of blood taken at the outset and conclusion of the study showed that there were no significant changes in patients’ haematology, liver function, or renal function (…)” | **-** | **-** | **-** | low | **-** | **-** | **-** | low | **A** | **A** | **A** | high |
| Geusens et al. 1994 | Fully reported inflammation. | Comment: Only reported that ESR was n.s. within and between groups. | **-** | **-** | **-** | low | **-** | **-** | **-** | low | **A** | **A** | **A** | high |
| Kremer et al. 1995 | CRP and ESR. | Comment: CRP and ESR are normally used as markers of inflammation, and other studies often report CRP and ESR when TNF-alfa and IL-1 beta are reported. Furthermore, TNF-alfa and IL-1 beta are reported significant. It is unlikely that CRP and ESR have not been measured. | **-** | **-** | **-** | low | **-** | **-** | **-** | low | **G** | **G** | **G** | high |
| Vargová et al. 1998 | (Function, typical pain measure, maybe inflammation) | Comment: Unclear risk since only an abstract in English is available, and the article is in another language. | **-** | **-** | **-** | unclear | **-** | **-** | **-** | unclear | **-** | **-** | **-** | unclear |
| Volker et al. 2000 | No. |  | **-** | **-** | **-** | low | **-** | **-** | **-** | low | **-** | **-** | **-** | low |
| Adam et al. 2003 | Fully reported function. | Comment: Only reported no effect on grip strength. Does not report results for FO vs. corn oil.  Quote: “Fish oil treatment reduced CRP in AID patients but not in WD patients.” | **-** | **-** | **-** | low | **A** | **A** | **A** | high | **C** | **E** | **D** | high |
| Lau et al. 2004 | Typical function measure. | Comment: Reports an unusual measure for function, physical domain for “CAIMS-2SF” (Chinese Arthritis Impact Measurement Scale 2-Short Form), however since the study was conducted in Hong Kong, this does not raise suspicion. | **-** | **-** | **-** | low | **-** | **-** | **-** | low | **-** | **-** | **-** | low |
| Sundrarjun et al. 2004 | No. |  | **-** | **-** | **-** | low | **-** | **-** | **-** | low | **-** | **-** | **-** | low |
| Berbert et al. 2005 | No. |  | **-** | **-** | **-** | low | **-** | **-** | **-** | low | **-** | **-** | **-** | low |
| Madland et al. 2006 | CRP. | Comment: CRP omitted due to cut-off of < 10 mg/L, which most patients had. N.s. differences between groups in other markers of inflammation. | **-** | **-** | **-** | low | **-** | **-** | **-** | low | **F** | **F** | **F** | low |
| Deutsch 2007 | No. | Comment: Uses WOMAC for both OA and RA in their study. WOMAC is not suitable for RA, so an appropriate measure for RA function and pain is missing. However, no suspicion of ORB. The objective of the study was to examine patients with chronic inflammation and included patients with CVD and/or OA and/or RA. | **H** | **H** | **H** | low | **H** | **H** | **H** | low | **-** | **-** | **-** | low |
| Galarraga et al. 2008 | No. |  | **-** | **-** | **-** | low | **-** | **-** | **-** | low | **-** | **-** | **-** | low |
| Das Gupta et al. 2009 | No. | Comment: What cannot be seen from the ORB matrix is that HAQ were reported to be measured, but no results reported. Grip strength was significant within group for control. | **-** | **-** | **-** | low | **E** | **E** | **E** | high | **-** | **-** | **-** | low |
| Gruenwald et al. 2009 | Inflammation. | Comment: Other laboratory evaluations have been made, but it is not specified which laboratory evaluations were made in the method-section. In their introduction they mention that OA involves inflammation and that *n*-3 FA can reduce inflammation, i.e., unlikely that inflammation have not been measured. | **-** | **-** | **-** | low | **-** | **-** | **-** | low | **G** | **H** | **G** | high |
| Park et al. 2013 | No. |  | **-** | **-** | **-** | low | **-** | **-** | **-** | low | **-** | **-** | **-** | low |
| Araújo et al. 2014 | Function. | Comment: Only report TJC as pain outcome, however it was n.s., i.e., it does not raise suspicion of other outcomes having been measured. Other clinical outcomes measured, but none were significant, i.e., unlikely that function was measured and analyzed but not reported. Only an abstract available. | **-** | **-** | **-** | low | **H** | **H** | **H** | low | **-** | **-** | **-** | low |
| Stebbings et al. 2014 | No. |  |  |  |  | low | **-** | **-** | **-** | low | **-** | **-** | **-** | low |
| Fu et al. 2015 | Function. | Comment: Report other clinical outcomes, e.g., SJC, morning stiffness, in addition to patient’s and physician’s global assessment of arthritis activity, which includes function. No outcomes for function mentioned in the trial registration. None of these were significantly different between the groups. | **-** | **-** | **-** | low | **H** | **I** | **I** | low | **-** | **-** | **-** | low |
| Kremer et al. 1986 | Fully reported pain, typical function measure, and inflammation. | Comment: No outcomes fully reported, but only an abstract available. Reports PM fatigue, which is not a typical outcome to report. Reports estimates for tender joints, which are significant, but not for pt. perception of pain, which is n.s. Function reported n.s. at week 14, no results for combined treatment periods. Compliance was measured by plasma lipids, hence blood tests were taken, so it is unlikely that inflammation was not measured. | **A** | **A** | **A** | high | **A** | **A** | **A** | high | **H** | **G** | **G** | high |
| Darlington & Ramsey 1987 | Any fully reported. | Comment: Only results at week 3 and 24, and not at the end of the treatment reported or only reported for one of the groups. They report that patients were assessed every 3 weeks, i.e., it is clear that measures were taken at week 12, but unclear if they were analyzed.  Only an abstract available. | **B** | **A** | **E** | high | **G** | **A** | **E** | high | **G** | **A** | **E** | high |
| Hernández-Cruz et al. 1988 | Inflammation, and fully reported pain and function measure. | Comment: No outcomes fully reported, but only an abstract available. Combined results from the groups because they found no differences between them, but reports highly significant changes between baseline and endpoint for pain and function for the combined groups. Inflammation is likely to have been measured. | **C** | **D** | **D** | high | **D** | **D** | **D** | high | **H** | **G** | **G** | high |
| Kremer et al. 1988 | Function, typical inflammation measures, and fully reported pain. | Comment: Other clinical measures evaluated, so it is unlikely that function have not been measured. Untypical inflammation measures reported. Reports result for pain in an unusual way using a regression analysis and only for the treatment group, i.e., it is likely that there was no difference between groups. Author contacted, but no response.  Quote: “A regression analysis of visit number and treatment group showed a significant improvement for tender joint of -0.88*0.42 pr. visit for increasing doses of fish oil (*p* = 0.03)”.  Only an abstract available. | **D** | **E** | **D** | high | **H** | **G** | **G** | high | **G** | **G** | **G** | high |
| Kremer et al. 1993 | Function and inflammation, and fully reported pain. | Comment: Only an abstract available and all measures are partially reported. Several clinical parameters reported and no laboratory parameters mentioned, but inflammation and function measured in another trial by same author with similar design.  Only an abstract available. | **A** | **A** | **A** | **high** | **G** | **G** | **G** | high | **H** | **G** | **G** | **high** |
| Lau et al. 1993 | Full reported pain on VAS scale, Ritche’s articular index, grip strenght, and CRP. | Comment: NSAID-requirement and ESR fully reported, others partially reported. NSAID-requirement was significantly different between the groups, ESR, pain on VAS, Ritchie’s articular index, grip strength, and CRP were n.s. | **A** | **A** | **A** | high | **A** | **A** | **A** | high | **-** | **-** | **-** | low |
| Lau et al. 1995 | Any fully reported. | Comment: Outcomes of pain, function and inflammation only reported non-significant. | **A** | **A** | **A** | high | **A** | **A** | **A** | high | **A** | **A** | **A** | high |
| Kolahi et al. 2010b | Function, inflammation, and fully reported pain. | Comment: No outcomes fully reported, but only an abstract available. No separate pain or function measure reported, however other clinical measures evaluated and other laboratory measures have been measured. Inflammation is likely to have been measured.  Quote: “Also in this study supplementation with low doses of omega-3 or vitamin E did not improve the clinical symptoms such as number of tender or swollen joints, disease activity score or global assessment of pain by patients.” | **C** | **A** | **A** | high | **G** | **G** | **G** | high | **H** | **G** | **G** | high |
| Kolahi et al. 2010a | Pain final value, function. | Comment: Result reported for TJC at baseline. Clearly measured after 3 months since TJC is included in DAS28. It is likely that change in TJC have been analyzed, but was n.s.. Unclear if function was measured, but not mentioned in the trial registration.  Quote: “Disease Activity Score 28 (DAS28) was calculated using the numbers of swollen and tender joints, high-sensitivity C-reactive protein (hsCRP) and patient general health by the patient using visual analog scale (VAS).” | **E** | **E** | **E** | high | **G** | **H** | **H** | low | **-** | **-** | **-** | low |
| Dawczynski et al. 2011 | Function and fully reported pain and inflammation. | Comment: Only baseline values reported for pain and inflammation, but unlikely that they were not measured at end of the trial. Pain outcome clearly measured again. No function outcome mentioned in the article or in the trial registration.  Quote: “Study candidates were excluded from the study at any time after enrollment for the following reasons: (…) inadequate control of arthritic symptoms (≥50% increase of the number of swollen or tender joints).” | **E** | **E** | **E** | high | **G** | **H** | **H** | low | **E** | **E** | **E** | high |
| Reed et al. 2014 | Fully reported function, inflammation and typical pain outcome. | Comment: For pain, number of patients using NSAID reported fully, some of which were significant, but only baseline value for TJC, MHAQ, CRP and ESR given. Unlikely that these measures were only measured at baseline. Pain not stated to be measured in trial registration (NCT00072982), however, other outcomes, not stated in the registration, are reported in article. | **E** | **H** | **G** | high | **E** | **G** | **G** | high | **E** | **E** | **E** | high |
| Yazdanpanah et al. 2014 | Inflammation, and fully reported pain and function | Comment: Reports that function and pain were reduced but p>0.05. Inflammation measures or other laboratory measures are not mentioned.  Quote: “After treatment, the level of WOMAC (physical functions, joint stiffness, pain severity) visual analogue scale (pain severity) were reduced in all groups (*p <* 0.5).”  Only an abstract available. | **C** | **A** | **A** | high | **A** | **A** | **A** | high | **H** | **H** | **H** | low |

CRP = c-reactive protein; CVD = dardiovascular disease; ESR = erythrocyte sedimentation rate; HAQ = health assessment questionnaire; ITT = intention-to-treat; MHAQ = modified health assessment questionnaire; MOS = marine oil supplement; NKS = Ninna Karsbaek Senftleber; n.s. = non-significant; OA = osteoarthritis; pt(s) = patient(s); RoORB = risk of outcome reporting bias; SJC = swollen joint count; SMN = Sabrina Mai Nielsen; TJC = tender joint count; Classifications [1]: A = States outcome analysed but only reported that result not significant (typically stating *p*-value > 0.05); B = States outcome analysed but only reported that result significant (typically stating p-value < 0.05); C = States outcome analysed but insufficient data presented to be included in meta-analysis or to be considered to be fully tabulated; D = States outcome analysed but no results reported; E = Clear that outcome was measured but not necessarily analysed; F = Clear that outcome was measured but not necessarily analysed; G = Not mentioned but clinical judgment says likely to have been measured and analysed; H = Not mentioned but clinical judgment says unlikely to have been measured; I = Clear that outcome was not measured. [1] Dwan, K., et al. (2010). “Assessing the potential for outcome reporting bias in a review: a tutorial.” Trials 11: 52.

**Table S5.** Meta-regression analysis on pain for rheumatoid arthritis patients. Results of meta-regression analyses *.

| **Variable** | **Total Trials, *k*** | | **SMD for Pain** | | **95% CI** | **τ^2^** | ***I*^2^** | ***p*-Value for Interaction** |
| --- | --- | --- | --- | --- | --- | --- | --- | --- |
| **All trials** | 22 | | −0.20 | | (−0.37 to −0.04) ^†^ | 0.054 | 32% | n.a. |
| **Supplementation type** | | | | | | 0.052 | 32% | 0.027 ^†^ |
| Capsule | 19 | | −0.17 | | (−0.35 to −0.00) ^†^ |  |  |  |
| Bottle | 0 | | - | | - |  |  |  |
| Unclear | 3 | | −0.50 | | (−1.04 to 0.04) |  |  |  |
| **Type of control** | | | | | | 0.062 | 34% | 0.121 |
| PUFA w/o EPA and DHA | 9 | | −0.15 | | (−0.42 to 0.12) |  |  |  |
| Non-PUFA oils | 7 | | −0.36 | | (−0.67 to −0.06) ^†^ |  |  |  |
| Non-oils | 2 | | −0.31 | | (−0.77 to 0.14) |  |  |  |
| Unclear | 2 | | 0.02 | | (−0.52 to 0.56) |  |  |  |
| None | 2 | | 0.13 | | (−0.55 to 0.81) |  |  |  |
| **Duration** | | | | | | 0.061 | 35% | 0.066 |
| <12 weeks | 1 | | 0.41 | | (−0.60 to 1.42) |  |  |  |
| ≥12 weeks and <24 weeks | 11 | | −0.23 | | (−0.46 to 0.00) |  |  |  |
| ≥24 weeks | 10 | | −0.21 | | (−0.47 to 0.04) |  |  |  |
| Unspecified | 0 | | - | | - |  |  |  |
| **Ratio of EPA/DHA** | | | | | | 0.056 | 33% | 0.060 |
| Ratio of EPA/DHA≤1.5 | 9 | | −0.21 | | (−0.46 to 0.04) |  |  |  |
| Ratio of EPA/DHA of >1.5 | 10 | | −0.26 | | (−0.50 to −0.02) ^†^ |  |  |  |
| Unspecified | 3 | | 0.12 | | (−0.43 to 0.67) |  |  |  |
| **Dosage of EPA plus DHA** | | | | | | 0.027 | 19% | 0.008 ^†^ |
| <2.6 g/day | 5 | | −0.46 | | (−0.74 to −0.19) ^†^ |  |  |  |
| ≥2.6 g/day and <3.6 g/day | 11 | | −0.18 | | (−0.40 to 0.04) |  |  |  |
| ≥3.6 g/day | 3 | | 0.05 | | (−0.32 to 0.42) |  |  |  |
| Unspecified | 3 | | 0.13 | | (−0−38 to 0.65) |  |  |  |
| **Source of marine oil** | | | | | | 0.051 | 30% | 0.062 |
| Whole fish | 19 | | −0.16 | | (−0.33 to 0.02) |  |  |  |
| Mussel | 1 | | −0.76 | | (−1.54 to 0.01) |  |  |  |
| Other | 1 | | −0.45 | | (−0.04 to 0.15) |  |  |  |
| Unspecified | 1 | | −0.11 | | (−1.00 to 0.79) |  |  |  |
| **Bias domains** | | | | | | | | |
| **Random sequence generation (selection bias)** | | | | | | 0.049 | 29% | 0.029 ^†^ |
| Adequate | 2 | | | −0.12 | (−0.60 to 0.36) |  |  |  |
| Unclear | 17 | | | −0.15 | (−0.34 to 0.03) |  |  |  |
| Inadequate | 3 | | | −0.57 | (−1.01 to −0.12) ^†^ |  |  |  |
| **Allocation concealment (selection bias)** | | | | | | 0.025 | 17% | 0.006 ^†^ |
| Adequate | 2 | | | −0.75 | (−1.29 to −0.21) ^†^ |  |  |  |
| Unclear | 18 | | | −0.19 | (−0.35 to −0.02) ^†^ |  |  |  |
| Inadequate | 2 | | | 0.09 | (−0.39 to 0.57) |  |  |  |
| **Blinding of participants (performance bias)** | | | | | | 0.037 | 24% | 0.020 ^†^ |
| Adequate | 5 | | | −0.06 | (−0.28 to 0.40) |  |  |  |
| Unclear | 8 | | | −0.33 | (−0.58 to −0.08) ^†^ |  |  |  |
| Inadequate | 9 | | | −0.22 | (−0.46 to 0.02) |  |  |  |
| **Blinding of personnel (performance bias)** | | | | | | 0.065 | 36% | 0.078 |
| Adequate | 4 | −0.22 | | | (−0.63 to 0.18) |  |  |  |
| Unclear | 12 | −0.27 | | | (−0.49 to −0.05) ^†^ |  |  |  |
| Inadequate | 6 | −0.04 | | | (−.038 to 0.30) |  |  |  |
| **Incomplete outcome data (attrition bias)** | | | | | | 0.076 | 39% | 0.114 |
| Adequate | 2 | 0.00 | | | (−0.54 to 0.55) |  |  |  |
| Unclear | 5 | −0.22 | | | (−0.64 to 0.20) |  |  |  |
| Inadequate | 15 | −0.23 | | | (−0.44 to −0.03) |  |  |  |
| **Outcome reporting (outcome reporting bias for pain)** | | | | | | 0.0461 | 30% | 0.013 |
| Adequate | 20 | −0.17 | | | (−0.34 to −0.01) ^†^ |  |  |  |
| Unclear | 0 | - | | | - |  |  |  |
| Inadequate | 2 | −0.72 | | | (−1.39 to −0.05) ^†^ |  |  |  |
| **Funding source** | | | | | | 0.059 | 33% | 0.047 |
| Industry only | 5 | −0.25 | | | (−0.56 to 0.07) |  |  |  |
| Mixed | 6 | 0.00 | | | (−0.32 to 0.33) |  |  |  |
| Nonprofit only | 4 | −0.36 | | | (−0.73 to 0.01) |  |  |  |
| Not reported | 5 | −0.07 | | | (−0.46 to 0.31) |  |  |  |
| Unclear | 2 | −0.73 | | | (−1.36 to −0.10) ^†^ |  |  |  |

* Analyzed using a random-effects restricted maximum of likelihood (REML)- based meta-regression model; †: *p* < 0.05; 95% CI = 95% confidence interval; *I^2^* = heterogeneity; PUFA = polyunsaturated fatty acid; SMD = standardized mean difference; k = number of trials; *τ^2^* = estimated between-study variance.

**Table S6.** Meta-regression analysis on function. Results of meta-regression analyses *.

| **Variable** | **Total Trials, *k*** | **SMD for Function** | **95% CI** | **τ^2^** | ***I*^2^** | ***p*-Value for Interaction** |
| --- | --- | --- | --- | --- | --- | --- |
| **All trials** | 23 | −0.01 | (−0.19 to 0.18) | 0.128 | 60% | n.a. |
| **Diagnoses** | | | | 0.018 | 17% | <0.001 |
| RA | 16 | 0.05 | (−0.11 to 0.21) |  |  |  |
| OA | 5 | 0.11 | (−0.13 to 0.35) |  |  |  |
| Other | 2 | −1.01 | (−1.43 to −0.59) ^†^ |  |  |  |
| Unclear |  |  |  |  |  |  |
| **Supplementation type** | | |  | 0.132 | 61% | 0.715 |
| Capsule | 20 | 0.02 | (−0.18 to 0.23) |  |  |  |
| Bottle | 3 | −0.22 | (−0.77 to 0.33) |  |  |  |
| Unclear | 0 |  |  |  |  |  |
| **Type of control** | | | | 0.144 | 63% | 0.792 |
| PUFA w/o EPA and DHA | 8 | 0.04 | (−0.31 to 0.38) |  |  |  |
| Non-PUFA oils | 8 | 0.13 | (−0.18 to 0.44) |  |  |  |
| Non-oils | 3 | −0.30 | (−0.80 to 0.19) |  |  |  |
| Unclear | 3 | −0.15 | (−0.72 to 0.42) |  |  |  |
| None | 1 | −0.03 | (−1.18 to 1.12) |  |  |  |
| **Duration** | | |  | 0.026 | 23% | <0.001 |
| <12 weeks | 3 | −0.83 | (−1.22 to −0.43) |  |  |  |
| ≥12 weeks and <24 weeks | 9 | −0.01 | (−0.22 to 0.21) |  |  |  |
| ≥24 weeks | 11 | 0.14 | (−0.05 to 0.33) |  |  |  |
| Unspecified | 0 |  |  |  |  |  |
| **Ratio of EPA/DHA** | | |  | 0.144 | 62% | 0.955 |
| Ratio of EPA/DHA≤1.5 | 10 | 0.03 | (−0.27 to 0.33) |  |  |  |
| Ratio of EPA/DHA of >1.5 | 9 | −0.07 | (−0.37 to 0.23) |  |  |  |
| Unspecified | 4 | 0.07 | (−0.40 to 0.53) |  |  |  |
| **Dosage of EPA plus DHA** | | |  | 0.157 | 64% | 0.993 |
| <2.6 g/day | 6 | 0.01 | (−0.36 to 0.38) |  |  |  |
| ≥2.6 g/day and <3.6 g/day | 9 | −0.07 | (−0.40 to 0.27) |  |  |  |
| ≥3.6 g/day | 4 | 0.03 | (−0.43 to 0.50) |  |  |  |
| Unspecified | 4 | 0.07 | (−0.41 to 0.55) |  |  |  |
| **Bias domains** | | | | | | |
| **Random sequence generation (selection bias)** | | |  | 0.137 | 60% | 0.62 |
| Adequate | 3 | −0.28 | (−0.76 to 0.20) |  |  |  |
| Unclear | 18 | 0.03 | (−0.19 to 0.25) |  |  |  |
| Inadequate | 2 | 0.20 | (−0.45 to 0.86) |  |  |  |
| **Allocation concealment (selection bias)** | | |  | 0.150 | 63% | 1.00 |
| Adequate | 2 | 0.13 | (−0.56 to 0.82) |  |  |  |
| Unclear | 20 | −0.01 | (−0.22 to 0.20) |  |  |  |
| Inadequate | 1 | 0.13 | (−1.00 to 0.75) |  |  |  |
| **Blinding of participants (performance bias)** | | |  | 0.129 | 60% | 0.42 |
| Adequate | 8 | −0.22 | (−0.53 to 0.09) |  |  |  |
| Unclear | 7 | 0.11 | (−0.22 to 0.46) |  |  |  |
| Inadequate | 8 | 0.11 | (−0.21 to 0.44) |  |  |  |
| **Blinding of personnel (performance bias)** | | |  | 0.119 | 58% | 0.29 |
| Adequate | 3 | 0.21 | (−0.70 to 0.28) |  |  |  |
| Unclear | 14 | −0.08 | (−0.31 to 0.15) |  |  |  |
| Inadequate | 6 | 0.32 | (−0.07 to 0.70) |  |  |  |
| **Blinding of outcome assessment (detection bias)** | | |  | 0.134 | 62% | 0.75 |
| Adequate | 4 | −0.01 | (−0.44 to 0.43) |  |  |  |
| Unclear | 16 | −0.05 | (−0.27 to 0.18) |  |  |  |
| Inadequate | 3 | 0.33 | (−0.30 to 0.95) |  |  |  |
| **Incomplete outcome data (attrition bias)** | | |  | 0.132 | 60% | 0.48 |
| Adequate | 2 | −0.12 | (−0.75 to 0.52) |  |  |  |
| Unclear | 7 | −0.21 | (−0.55 to 0.13) |  |  |  |
| Inadequate | 14 | 0.12 | (−0.13 to 0.36) |  |  |  |
| **Outcome reporting (outcome reporting bias for function)** | | | | 0.140 | 62% | 0.99 |
| Adequate | 22 | −0.01 | (−0.20 to 0.19) |  |  |  |
| Inadequate | 1 | 0.05 | (−0.80 to 0.90) |  |  |  |
| **Funding source** | | |  | 0.115 | 57% | 0.35 |
| Industry only | 8 | 0.23 | (−0.05 to 0.52) |  |  |  |
| Mixed | 8 | −0.23 | (−0.55 to 0.10) |  |  |  |
| Non-profit only | 1 | −0.12 | (−0.92 to 0.67) |  |  |  |
| Not reported | 5 | −0.15 | (−0.57 to 0.26) |  |  |  |
| Unclear | 1 | 0.31 | (−0.70 to 1.32) |  |  |  |

†: *p* < 0.05; 95% CI = 95% confidence interval; I^2^ = heterogeneity; n.a. = not applicable; SMD = standardized mean difference; τ^2^ = estimated between-study variance.

**Table S7.** Meta-regression analysis on inflammation. Results of meta-regression analyses *.

| **Variable** | **Total Trials, *k*** | **SMD for Inflammation** | **95% CI** | **τ^2^** | ***I*^2^** | ***p*-Value for Interaction** |
| --- | --- | --- | --- | --- | --- | --- |
| **All** **trials** | 25 | −0.28 | (−0.51 to −0.06) | 0.214 | 70% | n.a. |
| **Diagnoses** | | |  | 0.152 | 61% | 0.00 |
| RA | 21 | −0.20 | (−0.42 to 0.03) |  |  |  |
| OA | 2 | −0.23 | (−0.87 to 0.41) |  |  |  |
| Other | 2 | −1.11 | (−1.78 to −0.44) ^†^ |  |  |  |
| **Supplementation type** | | |  | 0.23 | 72% | 0.10 |
| Capsule | 22 | −0.26 | (−0.50 to −0.02) ^†^ |  |  |  |
| Bottle | 1 | −0.30 | (−1.43 to 0.84) |  |  |  |
| Unclear | 2 | −0.60 | (−1.52 to 0.32) |  |  |  |
| **Type of control** | | |  | 0.244 | 72% | 0.19 |
| PUFA w/o EPA and DHA | 10 | −0.24 | (−0.61 to 0.13) |  |  |  |
| Non-PUFA oils | 7 | −0.27 | (−0.71 to 0.17) |  |  |  |
| Non-oils | 4 | −0.51 | (−1.06 to 0.03) |  |  |  |
| Unclear | 2 | 0.07 | (−0.73 to 0.87) |  |  |  |
| None | 2 |  |  |  |  |  |
| **Duration** | | |  | 0.161 | 63% | 0.01 |
| <12 weeks | 3 | −0.87 | (−1.46 to −0.28) ^†^ |  |  |  |
| ≥12 weeks and <24 weeks | 13 | −0.10 | (−0.38 to 0.17) |  |  |  |
| ≥24 weeks | 9 | −0.35 | (−0.69 to −0.00) ^†^ |  |  |  |
| Unspecified | 0 |  |  |  |  |  |
| **Ratio of EPA/DHA** | | |  | 0.210 | 69% | 0.02 |
| Ratio of EPA/DHA ≤ 1.5 | 12 | −0.16 | (−0.48 to 0.15) |  |  |  |
| Ratio of EPA/DHA of >1.5 | 10 | −0.28 | (−0.63 to 0.07) |  |  |  |
| Unspecified | 3 | −0.85 | (−1.53 to −0.16) ^†^ |  |  |  |
| **Dosage of EPA plus DHA** | | |  | 0.198 | 68% | 0.02 |
| <2.6 g/day | 7 | −0.46 | (−0.84 to −0.07) ^†^ |  |  |  |
| ≥2.6 g/day and <3.6 g/day | 11 | −0.12 | (−0.45 to 0.21) |  |  |  |
| ≥3.6 g/day | 4 | −0.02 | (−0.57 to 0.53) |  |  |  |
| Unspecified | 3 | −0.84 | (−1.51 to −0.17) ^†^ |  |  |  |
| **Bias** **domains** | | | | | | |
| **Random sequence generation (selection bias)** | | |  | 0.184 | 65% | 0.01 |
| Adequate | 3 | −0.82 | (−1.40 to −0.25) ^†^ |  |  |  |
| Unclear | 20 | −0.20 | (−0.44 to 0.04) |  |  |  |
| Inadequate | 2 | −0.15 | (−0.86 to 0.56) |  |  |  |
| **Allocation concealment (selection bias)** | | |  | 0.244 | 72% | 0.12 |
| Adequate | 3 | −0.27 | (−0.93 to 0.40) |  |  |  |
| Unclear | 21 | −0.29 | (−0.55 to −0.04) ^†^ |  |  |  |
| Inadequate | 1 | −0.18 | (−1.24 to 0.88) |  |  |  |
| **Blinding of participants (performance bias)** | | |  | 0.190 | 67% | 0.01 |
| Adequate | 9 | −0.55 | (−0.91 to −0.20) ^†^ |  |  |  |
| Unclear | 8 | −0.03 | (−0.41 to 0.33) |  |  |  |
| Inadequate | 8 | −0.21 | (−0.60 to 0.17) |  |  |  |
| **Blinding of personnel (performance bias)** | | |  | 0.240 | 72% | 0.12 |
| Adequate | 5 | −0.23 | (−0.75 to 0.28) |  |  |  |
| Unclear | 15 | −0.31 | (−0.60 to −0.02) ^†^ |  |  |  |
| Inadequate | 5 | −0.24 | (−0.79 to 0.31) |  |  |  |
| **Incomplete outcome data (attrition bias)** | | |  | 0.227 | 70% | 0.06 |
| Adequate | 2 | −0.60 | (−1.38 to 0.19) |  |  |  |
| Unclear | 6 | −0.42 | (−0.89 to 0.05) |  |  |  |
| Inadequate | 17 | −0.20 | (−0.47 to 0.08) |  |  |  |
| **Outcome reporting (outcome reporting bias for inflammation)** | | | | 0.215 | 70% | 0.03 |
| Adequate | 3 | −0.24 | (−0.48 to 0.00) |  |  |  |
| Inadequate | 4 | −0.52 | (−1.10 to 0.06) |  |  |  |
| **Funding source** | | |  | 0.177 | 65% | 0.02 |
| Industry only | 5 | 0.18 | (−0.27 to 0.63) |  |  |  |
| Mixed | 9 | −0.29 | (−0.63 to 0.04) |  |  |  |
| Non-profit only | 4 | −0.40 | (−0.93 to 0.14) |  |  |  |
| Not reported | 5 | −0.69 | (−1.17 to −0.21) ^†^ |  |  |  |
| Unclear | 2 | −0.30 | (−1.08 to 0.48) |  |  |  |

†: *p* < 0.05; 95% CI = 95% confidence interval; *I*^2^ = heterogeneity; n.a. = not applicable; SMD = standardized mean difference; τ^2^ = estimated between-study variance.


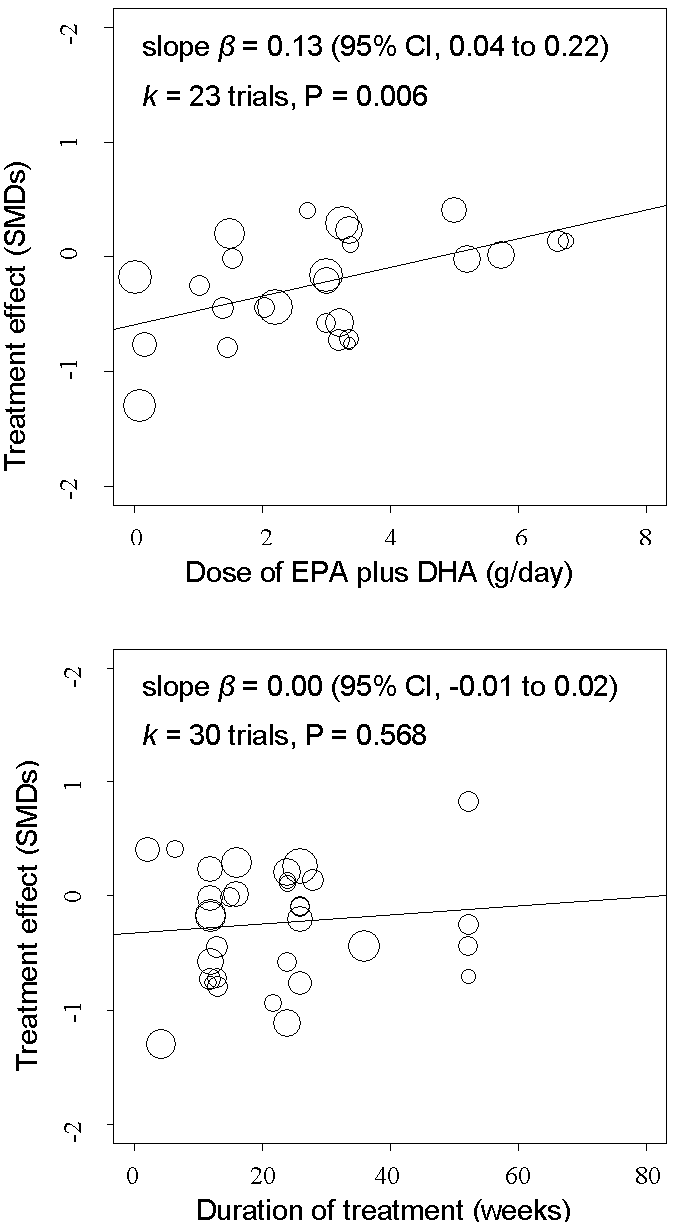


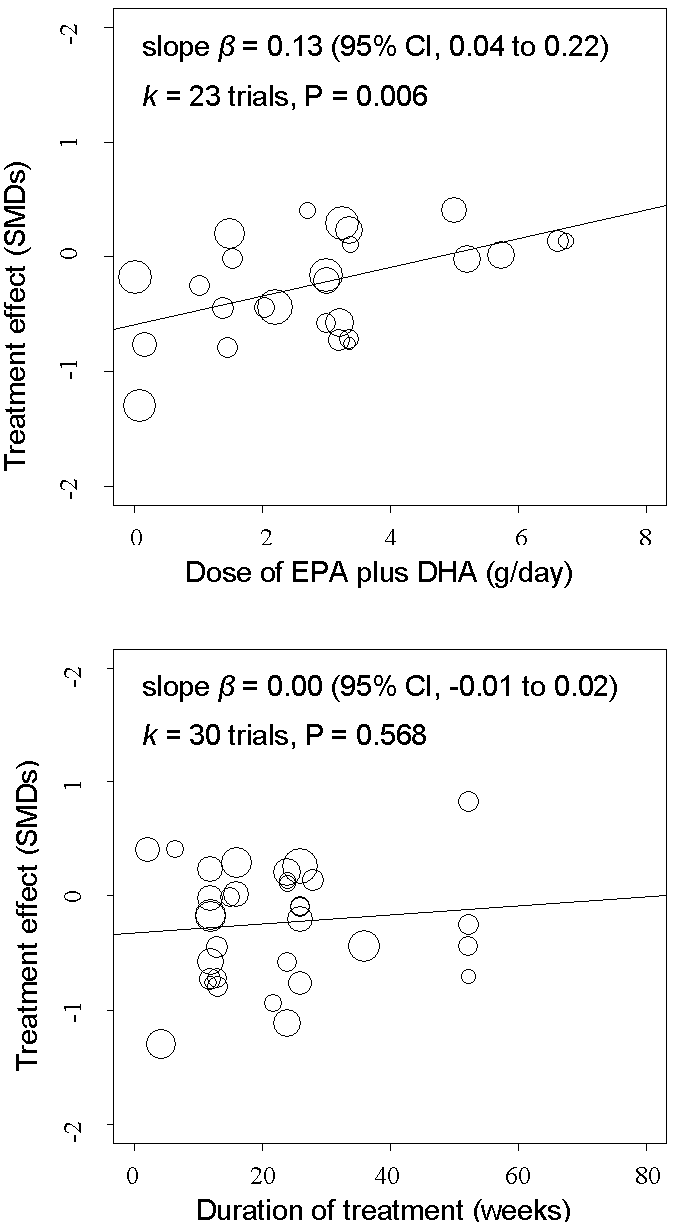


**Figure S1.** Meta-regression analysis (SMDs plotted against EPA plus DHA and duration of treatment). SMDs for pain on the vertical axis are plotted against the dose of EPA plus DHA (upper) and duration of treatment (lower). The size of the circles is proportional to the precision of each efficacy estimate. The solid line indicates the predicted effect (regression line) using a random-effects REML-based meta-regression model. DHA = docosahexaenoic acid; EPA = eicosapentaenoic acid; SMDs = standardized mean differences.


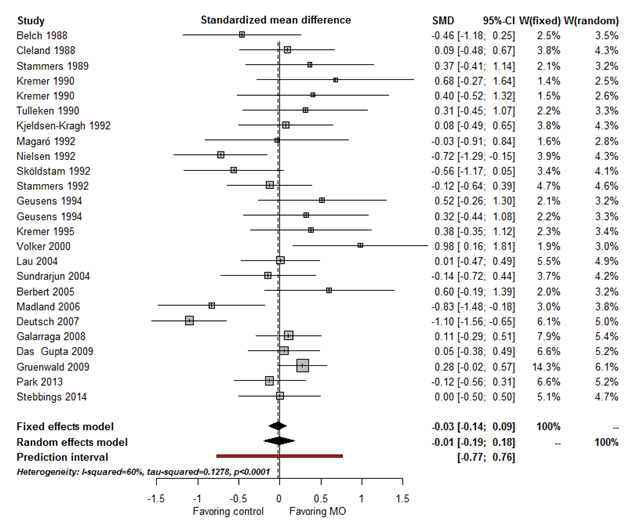


**Figure S2.** Forest plot for function. Forest plot for function, including trials with complete data. 95%-CI = 95% confidence interval; MO = marine oil; SMD = standardized mean difference; W = weight.


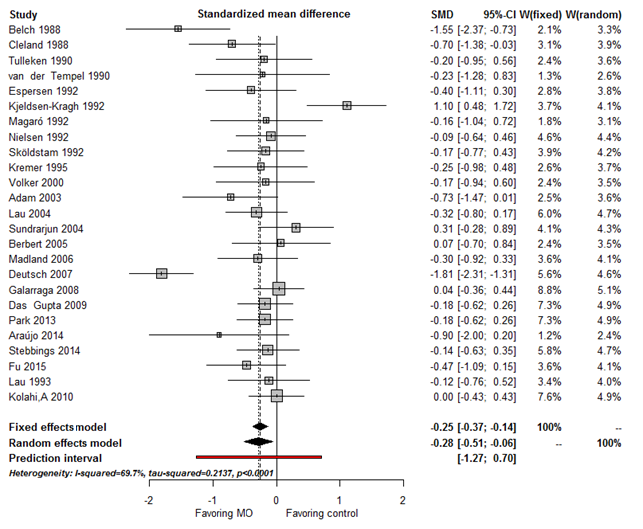


**Figure S3.** Forest plot for inflammation. Forest plot for function, including trials with complete data. 95%-CI = 95% confidence interval; MO = marine oil; SMD = standardized mean difference; W = weight.


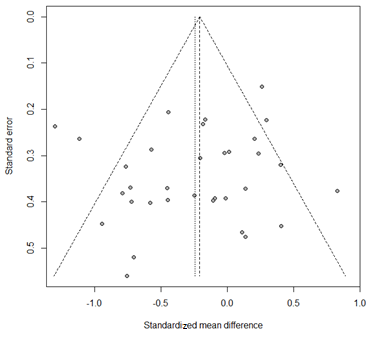


**Figure S4.** Funnel plot for pain.


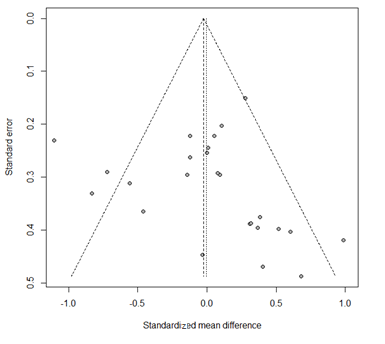


**Figure S5.** Funnel plot for function.


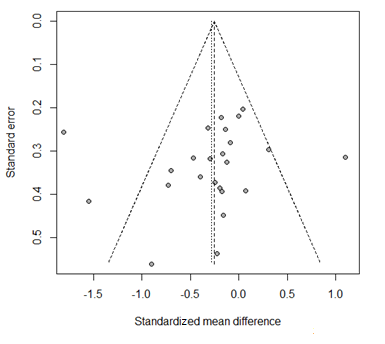


**Figure S6.** Funnel plot for inflammation.


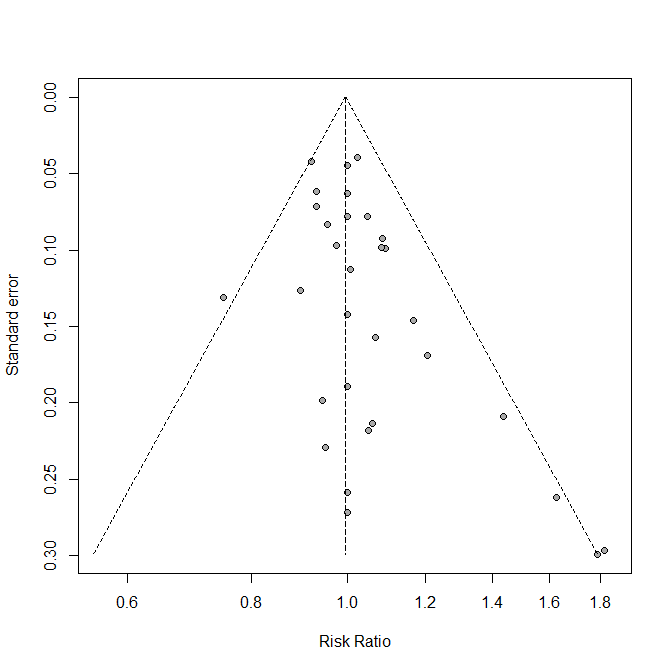


**Figure S7.** Funnel plot for tolerance.


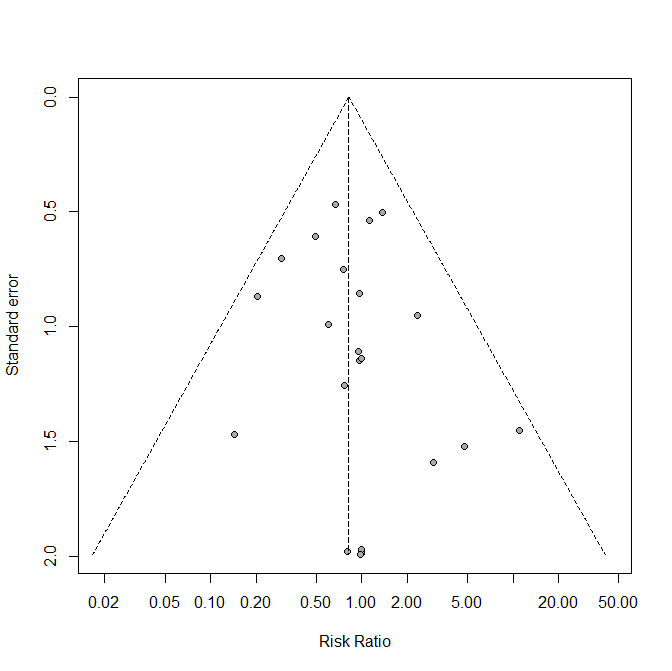


**Figure S8.** Funnel plot for withdrawals due to adverse events.


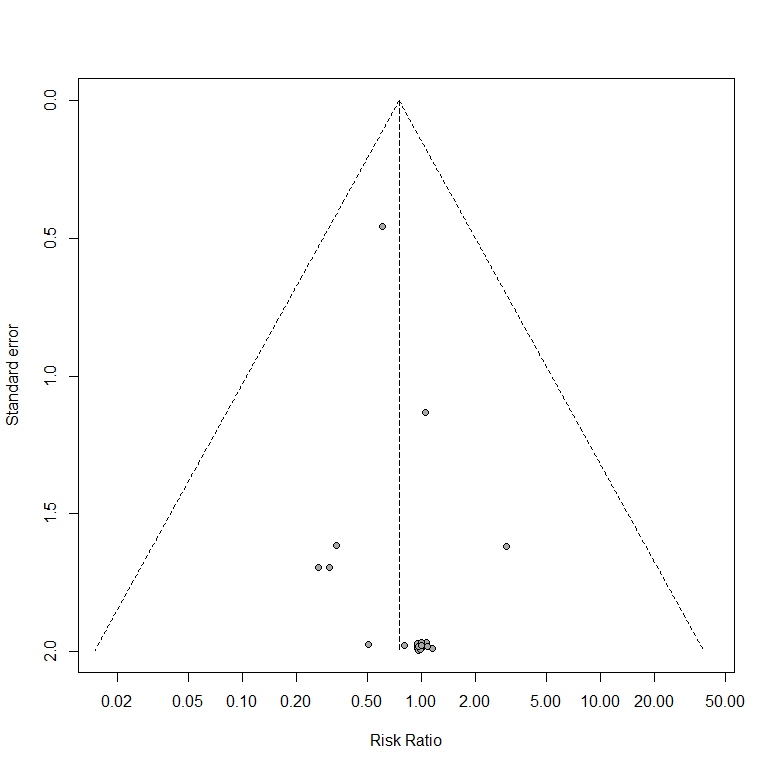


**Figure S9.** Funnel plot for serious adverse events.


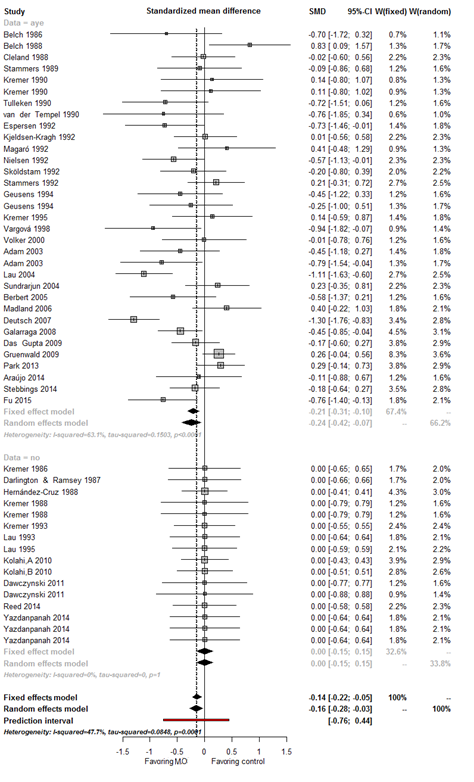


**Figure S10.** Forest plot for pain including trials with incomplete data. Trials, for which null-imputations have been used, are placed in the bottom part (“Data = no”). One trial appears two times (“Adam 2003”) because two parallel cross-over trials were performed. 95%-CI = 95% confidence interval; MO = marine oil; SMD = standardized mean difference; W = weight.


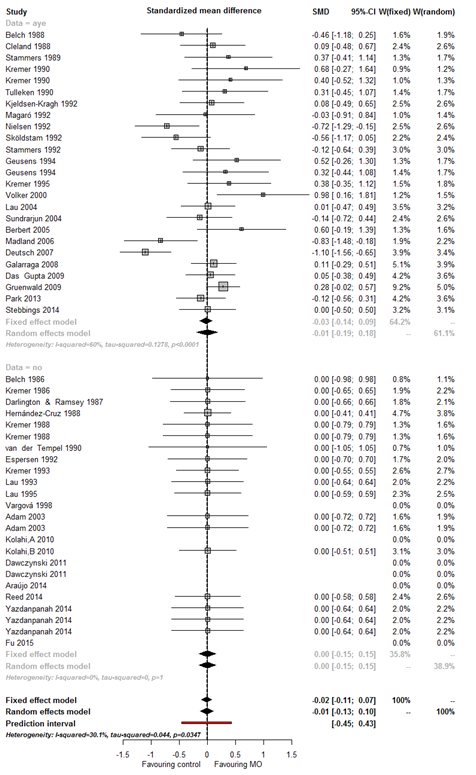


**Figure S11.** Forest plot for function including trials with incomplete data. Trials, for which null-imputations have been used, are placed in the bottom part (“Data = no”), together with trials given no effect estimate due to with incomplete data and low risk of bias. One trial appears two times (“Adam 2003”) because two parallel cross-over trials were performed. 95%-CI = 95% confidence interval; MO = marine oil; SMD = standardized mean difference; W = weight.


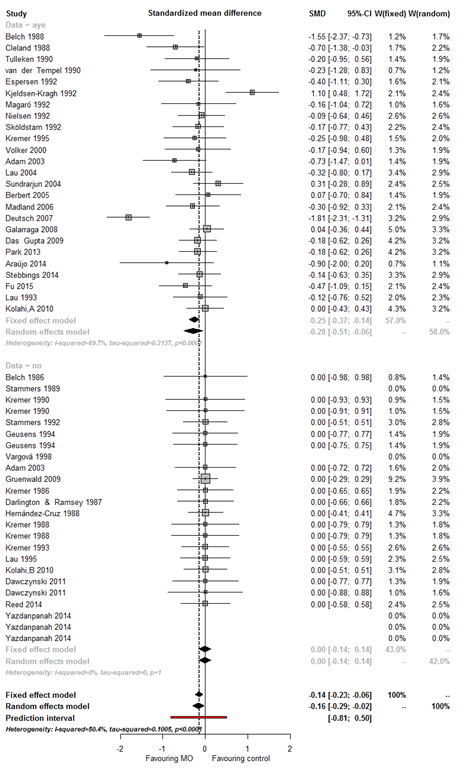


**Figure S12.** Forest plot for inflammation including trials with incomplete data. Trials, for which null-imputations have been used, are placed in the bottom part (“Data = no”), together with trials given no effect estimate due to with incomplete data and low risk of bias. One trial appears two times (“Adam 2003”) because two parallel cross-over trials were performed. 95%-CI = 95% confidence interval; MO = marine oil; SMD = standardized mean difference; W = weight.
